# The volume and characteristics of research on gastrointestinal symptoms in ‘natural’ peri- and postmenopause: A scoping review

**DOI:** 10.1177/17455057251387470

**Published:** 2025-10-27

**Authors:** Naomi Shaw, Rebecca Abbott, Clare Pettinger

**Affiliations:** 1 School of Health Professions, Intercity Place, University of Plymouth, UK; 2 Evidence Synthesis Team, University of Exeter Medical School, St Luke’s Campus, University of Exeter, UK

**Keywords:** menopause, perimenopause, postmenopause, gastrointestinal symptoms, gut health, scoping review

## Abstract

**Background:**

Menopause has been linked to an array of symptoms, often with adverse effects on quality of life, work and relationships. Despite evidence of economic and social impacts, and a growing population of menopausal individuals, there are significant gaps in knowledge regarding menopause. Gastrointestinal (GI) symptoms in peri- and postmenopause are areas of uncertainty that warrant further investigation.

**Objectives:**

Following JBI guidance, this scoping review aimed to systematically map research on GI symptoms in ‘natural’ peri- and postmenopause, exploring the volume and conduct of research, and variables investigated that could influence symptom experience.

**Eligibility criteria:**

Studies assessing GI symptoms (constipation, diarrhoea, vomiting, nausea, abdominal pain, heartburn, faecal incontinence and bloating) in ‘natural’ menopause from all publication dates were included. Studies of medical/surgical menopause, with participants on hormone therapies, or not published in English were excluded.

**Sources of evidence:**

Results of comprehensive searches of databases (MEDLINE, Embase, PsycINFO, CINAHL, AMED, Scopus, Web of Science, CENTRAL, ProQuest Dissertations and Theses), trial registries, citation and web-searches were screened against eligibility criteria.

**Charting methods:**

Data were charted in a standardised template, including study designs, populations, countries, GI symptoms studied, methods used for their assessment, and variables investigated. Analyses included frequency counts and percentages, with findings presented in visual and tabular formats.

**Results:**

Overall, 122 studies were included, published between 1981 and 2024. Studies were predominantly quantitative in design, with constipation the most frequently investigated symptom (*n* = 58), in contrast to vomiting (*n* = 4). Results highlighted limited studies from South American and African regions, and insufficient reporting of the criteria used to determine menopausal stages and assess GI symptoms.

**Conclusions:**

While many studies were identified, findings indicate evidence gaps and methodological considerations pertinent to researchers and funders, regarding study designs, assessment and reporting of menopausal stages and GI symptoms, and the variables investigated. Recommendations are made for future research.

## Introduction

In the UK, 13 million people are estimated to be peri- or postmenopausal,^1^ with 80%–90% of women thought to be negatively impacted by menopausal symptoms.^2^ ‘Natural’ menopause is reached when an individual has not had a menstrual cycle in the prior 12 months, due to a decline in ovarian function associated with ageing, rather than from surgical or medical intervention.^3^ Menopause commonly occurs between 44 and 54 years of age;^4^ however, it is proposed to be a ‘process’ rather than a single event.^5^ The Stages of Reproductive Ageing (STRAW+10) outlines the stages of menopause, with the transition from reproductive life to one year following the final menstrual period (FMP) defined as the ‘perimenopause’.^6^ The period leading up to the FMP is further categorised into the early and late menopausal transition (MT). Each stage is characterised by variability in menstrual cycle length and regularity, and significant fluctuations in levels of sex hormones including oestrogen, progesterone, luteinising hormone, and follicle-stimulating hormone.^7^ This ‘reproductive hormonal milieu’ and the gradual decline in sex hormones following the FMP have been associated with an array of symptoms,^8^ including hot flushes, night sweats, sleep disturbances, joint aches, vaginal dryness, brain fog, depression and anxiety.^7^ Despite a growing global population of menopausal individuals,^3^ and evidence of economic and social impacts associated with persistent menopausal symptoms,^1,8,9^ researchers argue that there are significant gaps in our understanding of menopause and the factors that influence the experience of symptoms.^10,11^

Gastrointestinal (GI) symptoms in peri- and postmenopause are one such area of uncertainty. GI symptoms as outlined by the National Institutes of Health (NIH) Patient Reported GI Symptom Scale^12^ include diarrhoea, constipation, nausea, vomiting, abdominal pain, bloating, heartburn and faecal incontinence. These symptoms are highly prevalent in the general population, placing a significant burden on health systems.^13,14^ Recent news coverage and sources of patient information have suggested an association between menopause and heightened risk of GI symptoms.^15,16^ However, GI symptoms are not acknowledged as key menopausal symptoms in international and UK guidelines.^17,18^ This may be due to the omission of GI symptoms from menopausal symptom assessment scales commonly used in research,^19–23^ or the limited availability of high-quality evidence from human studies to underpin recommendations.

Exploratory searches were conducted using MEDLINE (Ovid), JBI Evidence Synthesis, Open Science Framework (OSF) and Epistemonikos (February 2024) to gauge available evidence on GI symptoms in the peri- and postmenopause. Searches identified primary studies, but few evidence syntheses, with one scoping review exploring swallowing difficulties and symptoms of the oral cavity only,^24^ and one systematic review^25^ examining the relationship between menopausal stage and irritable bowel syndrome (IBS), a condition with symptoms including abdominal pain and dysfunctional bowel habits.^26^ While the systematic review by Adeyemo et al.^25^ found limited evidence for an association, searches were restricted to the PubMed database, therefore potentially missing eligible studies, and focused on GI symptoms linked to IBS only. Furthermore, new research has been published since the searches by Adeyemo et al.^25^ were completed in 2010. These new studies highlight conflicting findings regarding the relationship between menopausal stage and the frequency or severity of GI symptoms, including those common to IBS. For example, higher rates of constipation^27^ and faecal incontinence^28^ have been reported in postmenopausal compared with premenopausal individuals, while in contrast, other studies found no significant differences in the prevalence or severity of constipation,^29–31^ faecal incontinence,^32^ bloating^33^ or abdominal pain^34^ across menopausal stages. Indeed, some studies have found that premenopausal women were more likely to be affected by GI symptoms, such as chronic constipation^35^ and abdominal pain^30^, than women in later stages. These contradictory findings might be explained by the heterogeneity of definitions and methods used in these studies and indicate the need for an up-to-date review of research in this area.

**Figure 1.**
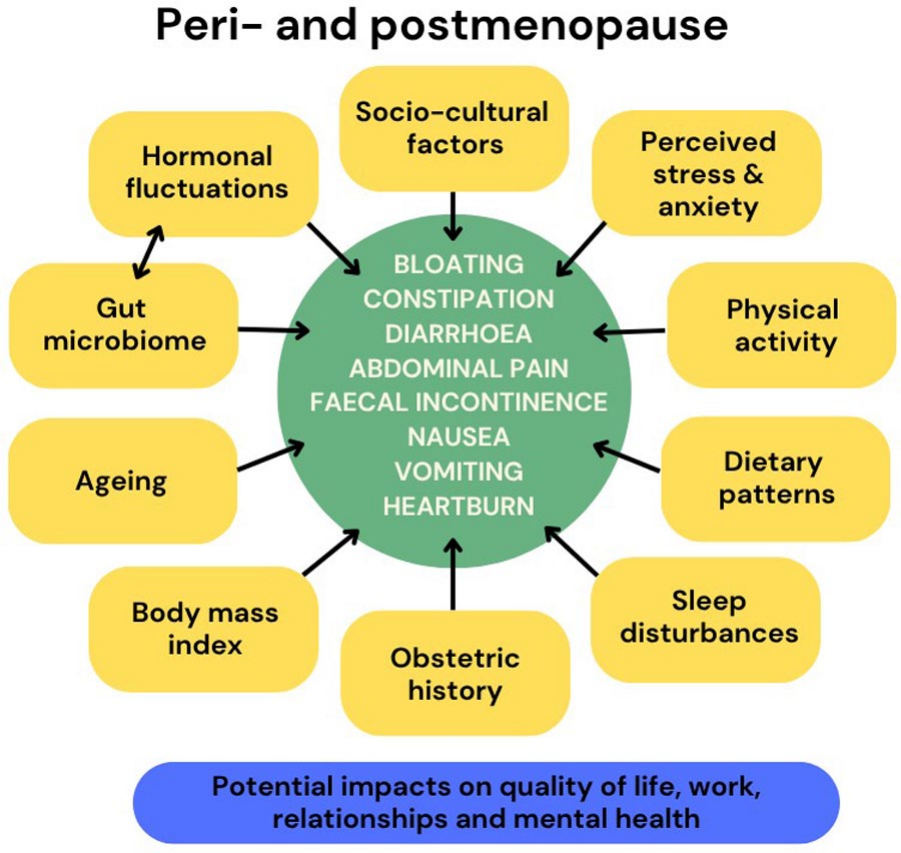
Possible correlates and risk factors associated with increased risk of GI symptoms in peri- and postmenopause. Sources: Nie et al.,^37^ Coquoz et al.,^39^ Zia and Heitkemper,^41^ Shieh et al.,^42^ Drossman,^46^ Thomas et al.,^49 Meleine and Matricon,^50^ Mulak et al.,^51^ Hogan et al.^52^^ GI: gastrointestinal.

Evidence suggests there may be ethnic and geographic variability in menopausal symptom prevalence and severity,^36^ and that an individual’s experience of menopause can be influenced by many hormonal, psychological and sociocultural factors (see Figure 1). Sex hormones such as oestrogen and progesterone, are thought to play an important role in GI health.^37–39^ Fluctuations in these sex hormones associated with the MT could lead to GI symptoms, through altered perceptions of pain,^40^ changes in gut motility,^41^ intestinal mucosa barrier functions,^42^ and immune and inflammatory processes.^37^ The composition of the gut microbiome may be impacted by hormonal changes, while also contributing to sex hormone metabolism.^43,44^ Such changes within the gut could result in the onset of new GI symptoms^45,46^ or exacerbations in individuals diagnosed with IBS or inflammatory bowel diseases.^47^ However, Yang et al. ^48^ suggest there is insufficient evidence to determine whether microbial changes triggered by hormonal fluctuations contribute to the experience of GI symptoms in the menopause.

Psychological and social variables may also contribute to GI and menopausal symptoms. The timing of the MT may co-occur with multiple stressors, such as additional caretaking responsibilities for elderly parents or children, bereavement, alongside frustrations with work roles and changing personal identities.^53^ Such sociocultural factors could contribute to increased susceptibility to psychological distress,^49^ or negatively impact sleep quality and dietary patterns, which in turn could initiate or intensify existing GI symptoms.^46,48^

A lack of funding for women’s health research has resulted in knowledge gaps related to menopause.^10,11,54^ Menopause is growing in visibility in the UK media^55^ and the recent Women’s Health Strategy for England^11^ established menopause as a key priority for future research investment. In consequence, this is an opportune time to identify evidence gaps and key areas for further study. With no recent or ongoing evidence syntheses exploring GI symptoms in peri- and postmenopause, and sufficient studies for a scoping review, these are areas of uncertainty that warrant further investigation. In consequence, this scoping review aimed to systematically search for, select and map research on GI symptoms in ‘natural’ peri- and postmenopause to identify gaps in the evidence to inform future research. The review had the following three objectives:

- To gauge the *volume* of evidence from published and grey literature examining patient-reported GI symptoms (nausea, vomiting, bloating, constipation, diarrhoea, heartburn, abdominal pain and faecal incontinence) in ‘natural’ peri- and postmenopause.
- To consider how research on GI symptoms in ‘natural’ peri- and postmenopause has been *conducted* (including study designs; measures of GI symptoms; timeframes of symptom recall; menopausal stages studied, and the criteria used to determine stages).
- To identify the *key variables* that have been measured in research on GI symptoms in ‘natural’ peri- and postmenopause (for e.g. correlates of GI symptoms: sex hormone levels, body mass index, perceived stress, ethnicity).

## Methods

A scoping review methodology was selected to address the research objectives as it is deemed appropriate for broad questions with the goal of exploring the volume, type and characteristics of available evidence.^56^

Methods for this review aligned with JBI guidance for the conduct of scoping reviews^57,58^ using systematic and reproducible methods to search for, select and map relevant studies, thus minimizing bias. Review reporting was guided by the Preferred Reporting Items for Systematic Reviews and Meta-Analyses Scoping Review (PRISMA-ScR) extension^59^ for transparency, with the completed checklist available in Supplemental Appendix 1.

As this is a review of existing literature, ethical approval was not necessary.

### Protocol

A protocol was registered on OSF (https://osf.io/4w5xc/). Deviations from methods outlined in this protocol are reported with justifications in Supplemental Appendix 2.

### Eligibility criteria

Eligibility criteria were pre-specified, defining the scope of this review, supporting development of search strategies, and facilitating decision-making regarding the relevance of records identified through comprehensive searches.^57^ The JBI Participant, Concept, Context framework^57^ was used to categorise eligibility criteria, as outlined in Table 1.

**Table 1.** Eligibility criteria for the identification and selection of studies.

Eligibility domain	Inclusion criteria	Exclusion criteria
Population	Studies of individuals described as experiencing: • ‘Natural’ perimenopause or • Menopausal transition or menopause • Postmenopause	Studies of individuals: • Experiencing hysterectomy, surgical, radiotherapy-induced or drug-induced menopause, or undergoing treatment for cancer • With chronic illness that influences menstrual cycles (e.g. polycystic ovary syndrome) • With primary ovarian insufficiency or premature menopause • Taking hormone therapies or hormonal contraceptives • Focusing only on pre-menopausal individuals (<40 years + experiencing regular periods) • With a mix of participants experiencing ‘natural’ and surgical, radiotherapy or drug-induced menopause, or taking hormone therapies, will be excluded if separate data is not provided • Describing older or midlife individuals that do not refer to menopausal status (i.e. the terms perimenopausal, menopausal or postmenopausal are not used)
Concept	Studies focusing on *patient-reported* GI symptoms^12^ including: • Bloating, abdominal pain, constipation, diarrhoea, nausea, vomiting, heartburn, faecal incontinence or describing non-specific GI or digestive symptoms • The above GI symptoms associated with disorders of gut-brain interaction (e.g. irritable bowel syndrome) • The above GI symptoms associated with organic GI disorders with an underlying diagnostic pathology (e.g. inflammatory bowel disorders, ulcerative colitis and Crohn’s disease) • Non-hormonal interventions for **treating** GI symptoms in perimenopausal or postmenopausal individuals	Studies that focus only: • On menopausal symptoms not related to the GI system (e.g. vasomotor, skin, genitourinary, mood symptoms) • On oral symptoms (dry mouth, swallowing difficulties), or symptoms not covered by the NIH PROMIS GI Scale (e.g. disordered eating)^12^ • On non-patient reported measures (e.g. electrophysiological measures, gut motility, visceral pain sensitivity, structural changes to the gut) • On GI symptoms as an adverse effect of: ○ Hormone replacement therapy or interventions for managing non-GI menopausal symptoms (e.g. selected oestrogen receptor modulators) ○ Interventions for other conditions (e.g. bisphosphonates for osteoporosis)
Context	All countries	–
Types of evidence	Ongoing/published primary research studies (including epidemiological, interventional, qualitative or mixed methods approaches) and evidence syntheses (systematic, scoping or rapid reviews) published in journals, dissertations, reports, grey literature, or clinical trial registries	Animal studies, laboratory studies that do not consider patient-reported GI symptoms, editorials and commentary, case studies, case reports, narrative and literature reviews, conference abstracts
Language	English-language publications	Non-English language publications
Publication date	All years	No restriction on publication date

#### Population

This review focuses on GI symptoms in ‘natural’ menopause, which is characterised by fluctuations in sex hormones during perimenopause, followed by a gradual decline postmenopause.^6^ Studies focusing on medically induced or surgical menopause were excluded, as these may cause an abrupt fall in oestrogen and progesterone,^36^ and could contribute to more severe menopausal symptoms.^60^ As hormone replacement therapies (HRT) may make it difficult to discern menopausal stage^61^ and could influence the experience of GI symptoms,^62^ studies recruiting individuals on HRT were excluded. Furthermore, as the menopausal stage of individuals with polycystic ovary syndrome, or those who have undergone hysterectomy, cannot be determined using menstrual cycle criteria,^6^ studies focusing on these populations were excluded.

Variable nomenclature has been used to classify menopausal stages,^61^ with the term ‘pre-menopausal’ inconsistently applied, sometimes being used to describe both the entire reproductive period, and also the 1–2 years prior to the cessation of menstruation. For clarity, studies of ‘pre-menopausal women’ only (aged <40 years with regular menstrual cycles) were excluded.

#### Concept

This scoping review focused on GI symptoms based on eight key categories described in the NIH Patient Reported GI Symptom Scale.^12^ To avoid duplication with a recent scoping review,^24^ studies on swallowing difficulties and oral symptoms were excluded. *Patient-reported* GI symptoms were the focus of this review, so studies reporting *only* laboratory or electrophysiological outcomes (such as GI motility or structural changes to the gut in menopause) were excluded.

#### Context

Studies from any country or region were included to facilitate identification of all *variables* (including ethnicity) studied that could impact on the experience of GI symptoms in peri- and postmenopause.

#### Language

Non-English publications were ineligible for inclusion due to translation costs, and the potential for language bias was noted.^63^ Language limits were not applied to searches, but instead, non-English publications were excluded during screening, as recommended by Pieper and Puljak.^64^

#### Publication dates

Studies from database inception to the search date were included, as this review aims to determine the *volume* of available evidence.

#### Types of evidence

To address objectives regarding the *volume* and *conduct* of available research, published and ongoing quantitative and qualitative primary research studies, and evidence syntheses were included. The following evidence sources were excluded: animal studies, laboratory studies (that did not assess *patient-reported* GI symptoms), editorials, case reports, literature reviews (that did not use systematic methods for the identification and selection of studies), and conference abstracts due to limited details of study methods and measures.

### Sources of evidence

As recommended by JBI guidance,^57^ searches were performed following a three-step process as outlined below:

#### Initial scoping searches

Text analysis of relevant studies from exploratory searches supported the identification of controlled vocabulary (e.g. Medical Subject Headings (MeSH)) and synonyms for the population (i.e. perimenopause) and the concept (i.e. named GI symptoms). An Information Specialist with expertise in evidence synthesis (NS) developed all search strategies. The Ovid MEDLINE strategy is presented in Box 1, with all other strategies reported in Supplemental Appendix 3.

**Box 1.** Search strategy for Ovid MEDLINE.

1 Exp menopause/ 2 (Menopaus* or perimenopaus* or peri-menopaus* or postmenopaus* or post-menopaus* or postreproductive or post-reproductive or climacteric).ti,ab. 3 1 or 2 4 Irritable bowel syndrome/ 5 Exp inflammatory bowel diseases/ 6 Exp “signs and symptoms, digestive”/ 7 ((Digestive or bowel or gut or gastro* or colonic) adj2 (symptom* or habit* or issue* or issue* or problem* or dysfunction* or complaint*)).ti,ab. 8 ((Digestive or gastro*) adj3 symptom*).ti,ab. 9 (GI adj symptom*).ti,ab. 10 IBS.ti,ab. 11 (Inflammatory adj bowel).ti,ab. 12 (Ulcerative adj colitis).ti,ab. 13 Crohn*.ti,ab. 14 (Irritable adj bowel).ti,ab. 15 (Diarrh* or constipat*).ti,ab. 16 ((Loose or watery) adj stool*).ti,ab. 17 ((Bowel* or defecat*) adj2 (frequen* or urgen* or infrequen*)).ti,ab. 18 (Incomplete adj evacuation).ti,ab. 19 (Bloating or bloated or gassiness or gaseousness or flatulence or flatulent or flatus or (abdom* adj disten*) or (swollen adj abdom*) or (swelling adj2 abdom*) or (postprandial adj fullness) or (post-prandial adj fullness)).ti,ab. 20 ((Gurgling or rumbling) adj2 (abdom* or stomach or gastro*)).ti,ab. 21 ((Abdom* or stomach or epigastric or rectal or rectum or belly) adj2 (pain* or cramp* or ache* or colic or discomfort)).ti,ab. 22 (Reflux or GERD or dyspepsia or indigestion or heartburn or regurgitat*).ti,ab. 23 (Belch* or burp* or eructation or hiccup*).ti,ab. 24 (Nausea* or vomit* or emesis or retching).ti,ab. 25 ((Faecal or fecal or anal or bowel) adj2 (incontinen* or leak* or soiling)).ti,ab. 26 ((Bowel* adj control*) or encopresis).ti,ab. 27 ((Gut or digestive or gastro* or bowel* or colon*) adj2 health*).ti. 28 Or/4-27 29 3 and 28 30 Exp animals/ not humans/ 31 29 not 30

#### Full database searches

The following bibliographic databases were searched on 4 March 2024 without date or publication type restrictions: MEDLINE, Embase, APA PsycINFO (Ovid); CINAHL, AMED (EBSCO); Cochrane Database of Systematic Reviews, Cochrane Central Register of Controlled Trials (Wiley); Web of Science Core Collection (Clarivate Analytics); Scopus (Elsevier) and ProQuest Dissertations and Theses Global. Search strategies were tailored for each database using appropriate syntax and controlled vocabulary.

#### Grey literature and supplementary searches

Searches of professional associations and key websites (e.g. British Menopause Society), Google/Google Scholar and clinical trials registries (ClinicalTrials.gov and the World Health Organization International Clinical Trial Registry Platform) were completed from April to June 2024 to identify grey literature.^65^ Simplified search strategies were used for Google/Google Scholar, with the first ten pages of each search screened by one reviewer. Full details are provided in Supplemental Appendix 3. Forwards and backwards citation-searching of included studies from database searches was completed using CitationChaser in May 2024,^66^ and bibliographies of key review articles were checked.

### Selection of sources of evidence

All records from searches were imported into EndNote X9.3.3 (Clarivate Analytics) and duplicates removed using manual checks and EndNote functionality.

#### Pilot screening

Pre-specified eligibility criteria (Table 1) informed the selection of studies. Three reviewers (NS, CP, KT) independently reviewed titles/abstracts of a random sample of 100 records in Rayyan^67^ to pilot eligibility criteria. Disagreements were discussed, and screening documentation was refined to ensure clarity.

#### Title/abstract and full-text screening

Screening by two independent reviewers is recommended to reduce the risk of bias or human error;^68^ however, due to time constraints, one reviewer screened 100% of records (NS), with a second reviewer (KT) screening 50% at both stages of screening. Firstly, titles/abstracts were assessed against eligibility criteria, followed by the full text of selected articles. Articles not available through University Library services were excluded, though citations are listed in Supplemental Appendix 4. Disagreements at both screening stages were resolved through consensus.

#### Data charting methods

A Microsoft Excel template was developed to chart data items relevant to review objectives. The following data items were charted: citation; study design; populations studied; GI symptom(s) investigated, as well as the methods (e.g. Menopause-Specific Quality of Life (MENQOL) checklist)^69^ and recall timeframe used in their assessment; definitions of menopausal stage (e.g. STRAW + 10^6^); countries; and variables studied (i.e. predictors, correlates or interventions). Additional items (e.g. new variables) were added to the template as the reviewer became familiar with the included literature. As this review is exploratory and does not aim to synthesise findings, study results were not extracted. This is consistent with guidance for the conduct and appropriate use of scoping review methods.^57^

The template was piloted by one reviewer (NS) on ten studies covering a range of evidence types. Independent data charting by two reviewers is recommended to minimise bias and human error;^58^ however, resource limitations required that one reviewer charted all data.

### Quality assessment

Quality assessment of included studies is not a mandatory step in scoping reviews^70^ and was not completed for this review.

### Data analysis and synthesis

Descriptive analyses focused on frequency counts of charted data, with percentages calculated using Excel (Microsoft; Version 2402) and presented in tabular, graphical or visual formats aligning with review objectives, as indicated in Table 2.

**Table 2.** Analyses by review objective.

Review objective/question	Frequency counts
What is the *volume* of evidence examining eight key categories of GI symptom in peri- and postmenopause?	Overall number of included studies
	Number of studies by year of publication
	Number of studies by publication type (i.e. journal article, dissertation, report)
	Number of studies by geographic location, with countries categorised into regions using WorldinData definitions^71^
	Number of studies by GI symptom (mapped to NIH PROMIS^12^ domains: diarrhoea, constipation, nausea, vomiting, bloating, faecal incontinence, heartburn and abdominal pain)
	Number of studies with a primary objective to investigate GI symptoms in ‘natural’ peri- or postmenopause
How has research on GI symptoms in natural peri- and postmenopause been *conducted*?	Number of studies by design (i.e. case-control, cross-sectional, longitudinal, randomised control trials, qualitative, systematic reviews)
	Number of studies by menopausal stage (i.e. perimenopausal, early menopausal transition, late menopausal transition, postmenopausal individuals)
	Number of studies by criteria used to assess menopausal stage (e.g. STRAW + 10^6^)
	Number of studies by measure used to assess GI symptoms (e.g. validated symptom scales)
	Number of studies by timeframe used for recall of GI symptoms (e.g. previous week; previous 12 months)
What *key variables* have been measured in research on GI symptoms in natural peri- and postmenopause?	Number of studies by variable (e.g. age, levels of sex hormones, perceived stress, ethnicity) to investigate the relationship between the variable and GI symptom frequency, severity, or prevalence Variables were grouped into demographics; lifestyle factors; clinical markers and comorbidities; biomarkers; menopause-related factors and interventions

## Results

**Figure 2.**
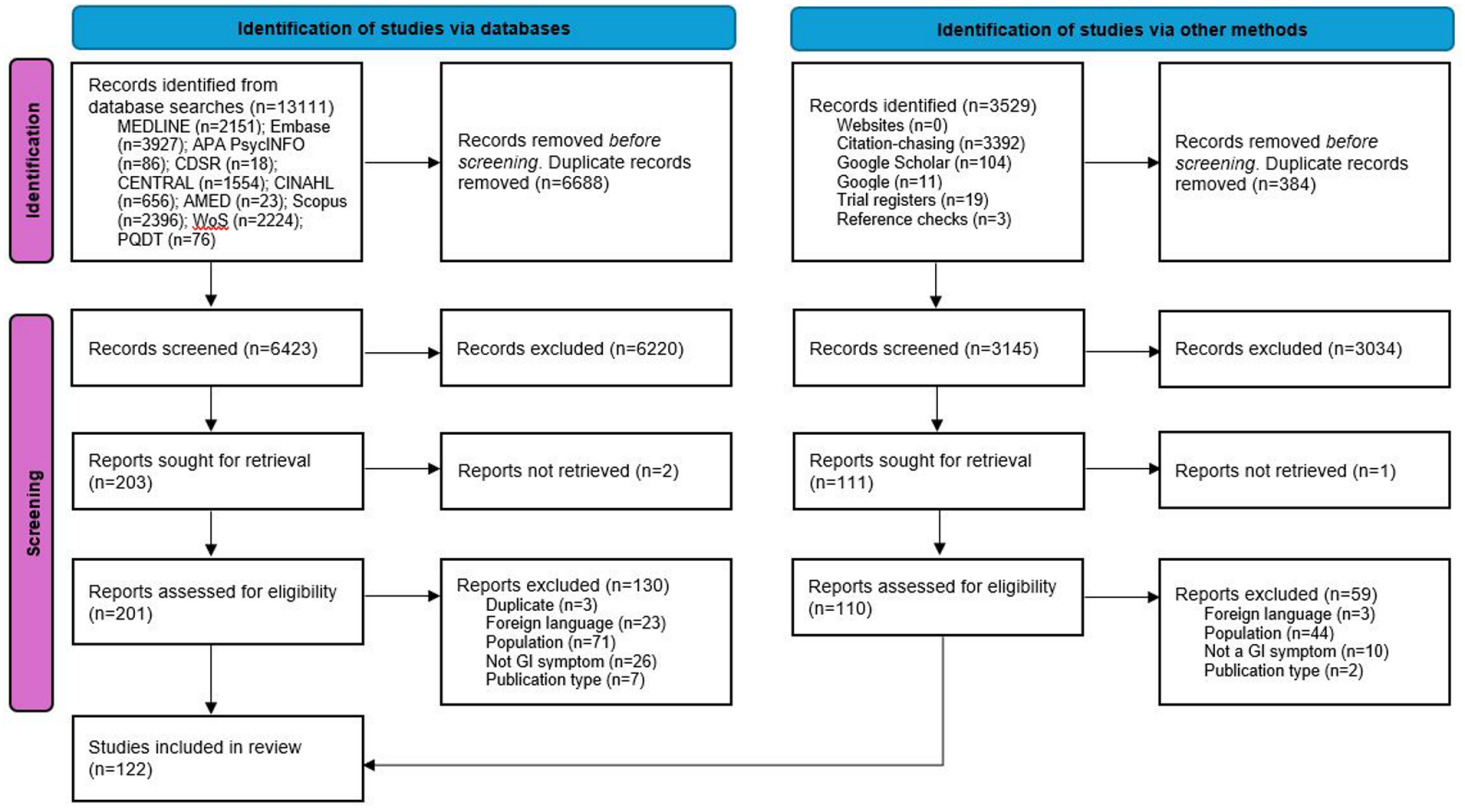
PRISMA flow diagram: study identification and selection.

Database and supplementary searches retrieved 13,111 and 3529 records, respectively. After duplicates were removed, 9569 records were assessed for eligibility from titles/abstracts. A total of 311 articles were retrieved for full-text screening, with 122 studies identified for inclusion. Figure 2 (PRISMA flow diagram) indicates the process of study selection. Details of excluded studies with reasons are available in Supplemental Appendix 4.

### Characteristics of included studies

Results are reported in alignment with review objectives, considering:

- The volume of evidence on GI symptoms in ‘natural’ peri- and postmenopause.
- The conduct of research.
- The variables investigated.

Study characteristics for 122 included studies outlining brief citation details, populations, GI symptom(s) assessed, study designs and variables investigated are provided in Supplemental Appendix 5.

#### Volume of evidence

**Figure 3.**
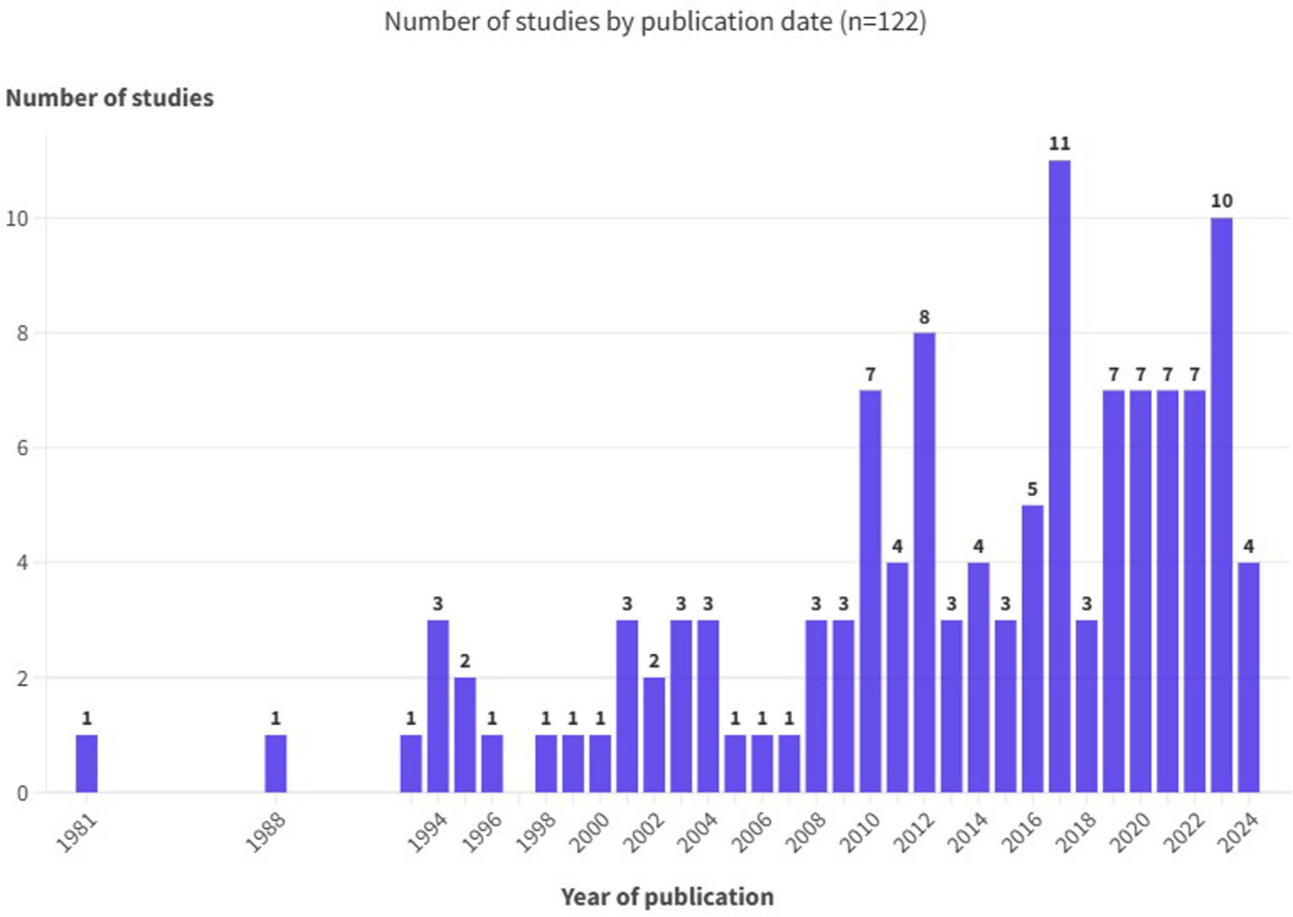
Number of included studies by publication date.

Figure 3 shows the timeline of included studies, with 34% (*n* = 42) published within the last 5 years.

Table 3 provides summary characteristics for included studies relating to the volume of evidence. This shows that most studies (*n* = 116; 95.1%) were published as journal articles, with one dissertation,^72^ two preprint publications,^73,74^ and three clinical trial registrations^75–77^ identified as ‘grey literature’. Studies were conducted in 35 countries represented by six geographic regions,^71^ ranging from only 0.8% (*n* = 1) in South America, compared with 44.3% (*n* = 54) carried out in Asia. From 122 included studies, only 18% (*n* = 22) stated a primary objective that related to the study of GI symptoms in ‘natural’ peri- or postmenopause, while 82% (*n* = 100) either investigated a GI symptom as one of a range of menopausal symptoms or assessed menopause as one potential risk factor for a GI symptom. Constipation was the most studied GI symptom (*n* = 58; 47.5%), while only 4 (3.3%) studies investigated vomiting in ‘natural’ peri- or postmenopause.

**Table 3.** Summary of study characteristics relating to the volume of available evidence (*n* = 122 included studies).

Studies by publication type: n/122 (%)			Studies with a primary objective relating to GI symptom(s) in peri- or postmenopause: *n*/122 (%)		
Journal article	116 (95.1)	^25,27–35,78–183^	Yes	22 (18)	^25,31,33,34,82,90–92,97,98,106,110,117–119,125,128,131,139,167,170,180^
Dissertation	1 (0.8)	^72^	No	100 (82)	^27–30,32,35,72–81,83–89,93–96,99–105,107–109,111–116,120–124,126,127,129,130,132–138,140–166,168,169,171–179,181–183^
Preprint	2 (1.6)	^73,74^			
Clinical trial registration	3 (2.4)	^75–77^			
Studies by geographical region: *n*/122 (%)			Studies by GI symptom: n/122 (%); (included studies investigating ⩾1 GI symptom)		
Asia	54 (44.3)	^27,28,33,35,73,76,77,81,84-87,90,96,99,106,108,110,121–131,133,135,136,138,144,146,148,158,159,161,162,164–166,168,169,171–173,177–179,181–183^	Constipation	58 (47.5)	^25,27,29–33,35,73–75,77–79,83,85,91,92,94,96,98,100–102,104,106,108,109,117–119,121,123,128,131–133,135,136,138,139,143–146,148,149,154,158,161,162,167,170–172,179,180,183^
North America	31 (25.4)	^29–31,34,72,75,83,93,95,101,107,111,113–120,132,134,139,143,145,149,152,167,170,175,176^	Bloating or flatulence	46 (37.7)	^25,33,72,74–76,78,79,84–87,89,95,98–102,105,109,111,112,115,117–120,124,126,127,129,130,132,134,137,141,142,158,166,169,173,175,178,181,182^
Europe	20 (16.4)	^80,88,89,91,92,97,98,105,109,140–142,147,150,151,157,160,163,174,180^	Diarrhoea	32 (26.2)	^25,27,29,31,33,73–75,77,83,85,92,98,100,101,104,108,115,117–119,131,132,136,139,146,149,162,170–172,180^
Oceania	8 (6.6)	^78,94,102–104,154–156^	Abdominal pain	27 (22.1)	^25,29,30,33,34,73,74,78,89,98,100,101,115–119,132,136,141,142,151,160,164,168,176,179^
Africa	4 (3.3)	^79,82,137,153^	Nausea	19 (15.6)	^25,29,74,78,93,100,101,107,117–120,127,132,136,137,141,152,177^
South America	1 (0.8)	^32^	Faecal incontinence	19 (15.5)	^28,32,81,83,88,90,96,110,122,125,135,138,140,148–150,157,163,165^
Multiple regions	3 (2.5)	^25,100,112^	Heartburn	18 (14.8)	^27,29,73,82,85,97,100,101,117–120,132,147,152,153,155,168^
Not stated	1 (0.8)	^74^	Vomiting	4 (3.3)	^74,117–119^
			Digestive symptoms – unspecified	8 (6.6)	^80,103,113,114,150,156,159,174^

#### Conduct of studies

Table 4 presents summary data on the conduct of studies on GI symptoms in ‘natural’ peri- and postmenopause.

**Table 4.** Summary of study characteristics relating to the conduct of research.

Studies by design: *n*/122 (%)			Studies by menopausal stage of recruited participants: *n*/122 (%)		
Systematic review	1 (0.8)	^25^	Peri	57 (46.7)	^30,31,34,72,74,76,77,82,84–86,89,92–94,99–105,107–109,115–120,123,126,130,131,133–136,138,141–144,153–156,164,166,172,174–178,182^
Quantitative	118 (96.7)					Randomised controlled trial	5 (4.1)	^82,89,106,139,170^	Peri only	9 (7.4)	^72,76,82,99,133,134,143,144,153^
Non-randomised trial	3 (2.4)	^97,98,131^	Post	107 (87.7)	^25,27–35,73–75,77–81,83–89,91,92,94–98,102–107,109–132,135–142,145–152,154–165,167–183^
Clinical trial registration	3 (2.4)	^75–77^	Post only	33 (27)	^73,75,87,91,97,98,106,110–112,121,122,124,127–129,137,139,140,146,150,151,158,159,161,162,167–171,173,181,183^
Cross-sectional	89 (73)	^27–30,32,33,35,72–74,79–81,83–87,90,92,93,95,96,99–102,104,105,107–120,122–130,132–138,141–144,146–148,150–153,158,159,161,164–166,168,169,171–175,177–182^	Criteria used for determining menopausal stage: *n*/122 (%)		
Longitudinal	14 (11.5)	^31,34,78,88,94,103,121,145,149,154–156,160,167^	Not stated	44 (36)	^25,28,29,32,33,35,76–78,80–82,88,90,95–97,99,107,108,110,121,122,124,129,132,138–140,145,147–149,152,157,159,161,163,165,166,168,170,171,179^
Case-control	3 (2.4)	^91,157,183^	Age-range	9 (7.4)	^83,89,109,126,143,144,153,167,176^
Retrospective	1 (0.8)	^140^			
Mixed method	1 (0.8)	^162^	STRAW/STRAW+10	13 (10.7)	^31,34,84,92,100,101,103,105,123,130,133,164,182^
Qualitative	2 (1.6)	163,176	Other definition	56 (45.9)	^27,30,72–75,79,85–87,91,93,94,98,102,104,106,111–120,125,127,128,131,134–137,141,142,146,150,151,154–156,158,160,162,169,172–175,177,178,180,181,183^
Measures used to assess GI symptoms: *n*/122 (%)			Timeframe reported for recall of GI symptoms: *n*/122 (%)			Not stated	9 (7.4)	^25,76,79,133,138,145,161,162,179^
Not stated	84 (68.8)	^25,27,28,32,72,73,76–79,81–83,86–89,91–93,96–99,101–103,108–121,123–128,130,131,133,135,137,138,140–147,149–152,157–159,161–164,166,168,169,171,173–176,178–180,182^	Validated measure	66 (54.1)	^28,33,35,72,73,75,77,81–84,86–89,92,95,103,106,110,112,115–132,134–136,139–143,146–149,151,156–158,160,169,171–175,177,178,181,182^
⩽1 week	6 (4.9)	^31,34,74,107,139,170^			
>1 week, ⩽1 month	14 (11.5)	^29,33,84,95,104–106,129,132,136,156,167,177,181^	Study-specific tool	47 (38.5)	^27,29–32,34,74,78,80,85,90,91,93,94,96–102,104,105,107–109,111,113,114,137,144,150,152–155,159,163–168,170,176,180,183^
>1 month, ⩽3 months	5 (4.1)	^30,75,80,122,148^			
>3 months–1 year	13 (10.7)	^35,85,90,94,100,134,153–155,160,165,172,183^

##### Study designs

Study designs were predominantly quantitative (*n* = 118; 96.7%), with only two (1.6%) qualitative,^163,176^ and one mixed-methods study (0.8%) included.^162^ One systematic review^25^ met eligibility criteria, and 73% of included studies were cross-sectional (*n* = 89), with other studies of longitudinal design (*n* = 14; 11.5%), randomised controlled trials (*n* = 5, 4%), non-randomised studies (*n* = 3; 2.5%), case-control (*n* = 3; 2.5%), and one retrospective study (0.8%).

##### Populations

Most included studies recruited individuals described as postmenopausal (*n* = 107; 87.7%), with 57 studies (46.7%) including those in perimenopause. Further analysis indicated that just over a third of included studies (*n* = 42; 34.4%) focused on individuals of *one menopausal stage only* (perimenopausal only: *n* = 9; postmenopausal: *n* = 33). Criteria or definitions used to determine menopausal stage were not stated in 36% of studies (*n* = 44), with age ranges used to identify individuals for inclusion in a further nine studies (*n* = 7.4%). STRAW/STRAW + 106,^184^ were cited as the criteria used for categorising menopausal stages in 13 studies (10.7%), while 56 (45.9%) provided alternative definitions (e.g. studies cited an adapted version of the WHO classification,^185^ or classified individuals as perimenopausal if they had experienced menstrual bleeding in the prior 12 months, but not in the last 3 months).

##### Measures of GI symptoms

Studies used a range of measures to assess GI symptoms, with 66 studies (54.1%) citing a validated menopausal or GI symptom screening tool (e.g. MENQOL,^186^ Wexner Incontinence Scale^187^). The remaining studies developed new assessment tools (*n* = 47; 38.5%) or did not describe the method used to assess GI symptoms (*n* = 9; 7.4%). The timeframe for recall of GI symptoms was not reported in 84 studies (68.8%), with other studies using varied recall periods ranging from less than or equal to a week (*n* = 6; 4.9%) to 3–12 months (*n* = 13; 10.7%).

#### Variables investigated

**Figure 4.**
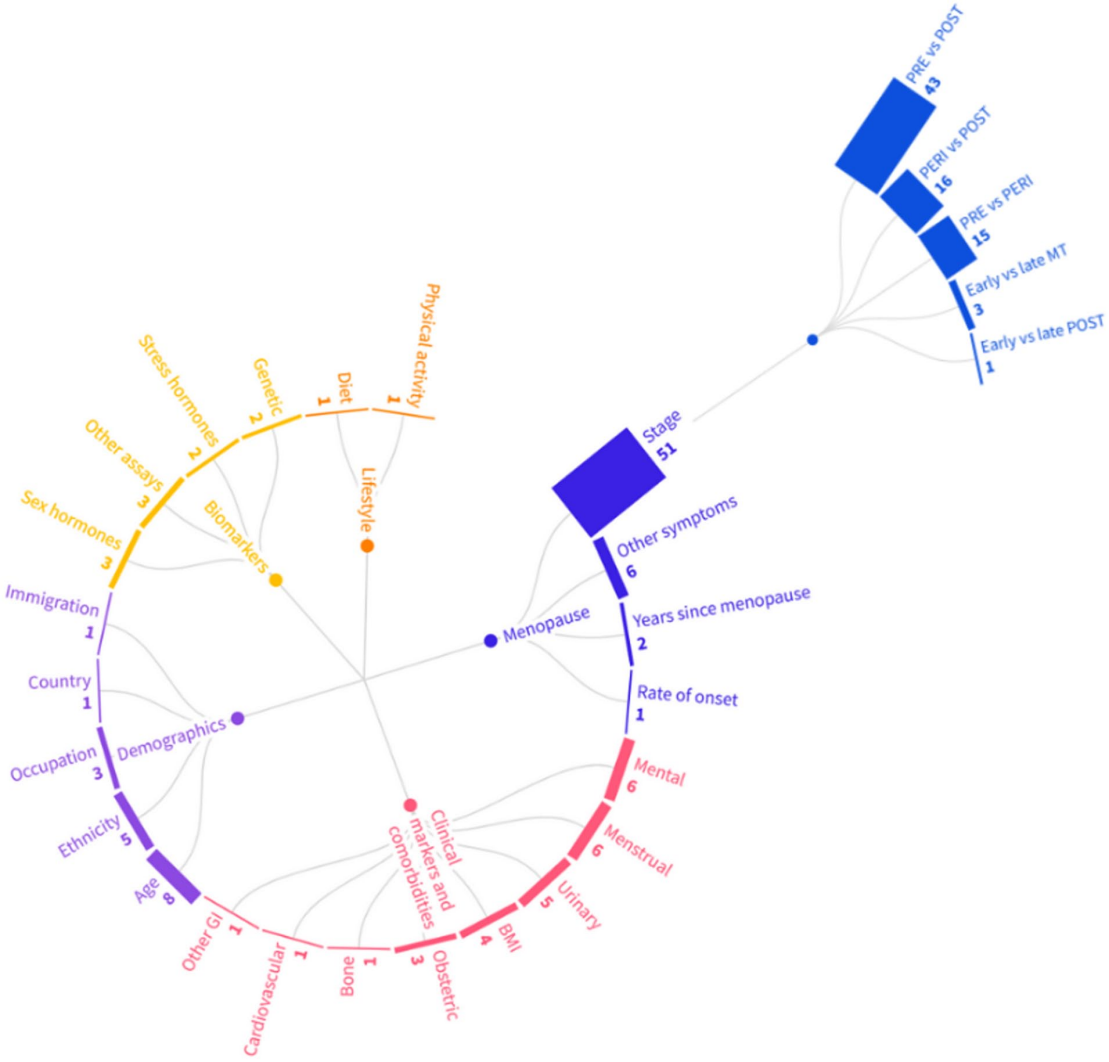
Number of studies by variables investigated in research on GI symptoms in peri- and postmenopause, including comparisons by menopausal stage. Created with Flourish. Available from: https://flourish.studio/. NB. Studies may include more than one comparison of menopausal stage. PRE: premenopausal stage; PERI: perimenopausal stage; POST: postmenopausal stage; MT: menopausal transition.

Included studies investigated a range of variables in association with GI symptoms in peri- and postmenopause (see Figure 4). These were categorised into: demographics, lifestyle factors, biomarkers, clinical markers and comorbidities, interventions, and menopause-related variables.

Menopause-related variables were the most studied category (*n* = 60; 49.2%). This category included associations of GI symptoms with other menopausal symptoms (such as hot flushes; *n* = 6; 4.9%),^74,92,105,107,144,155^ years since the FMP (*n* = 2; 1.6%)^88,122^ and rate of onset of menopause (*n* = 1; 0.8%).^137^ Comparisons of GI symptom frequency or severity by menopausal stage were observed in less than half of the included studies (*n* = 51, 41.8%). Symptom experience was compared in premenopausal with postmenopausal individuals in 43 studies (35.2%), while only three studies^31,34,182^ (2.4%) made a comparison between perimenopausal individuals in the early and late MT.

Demographic variables were assessed in 18 studies (14.8%), with 8 studies (6.6%) considering the impact of age^31,34,86,91,110,146,168,171^ on the experience of GI symptoms in menopause. Ethnicity or race,^115–117,119,120^ type of occupation,^86,111,158^ immigration status^118^ and country of residence^112^ were also considered in the included studies.

With regard to studies investigating comorbidities and clinical markers (*n* = 27; 22.1%), mental health including perceived stress and anxiety,^31,34,93,133,144,177^ and menstrual symptoms (such as dysmenorrhoea and premenstrual syndrome)^78,99,108,156,160,166^ were both assessed in six studies, while other included studies considered associations with body mass index,^79,91,130,150^ obstetric history or complications during childbirth,^125,157,163^ urinary disorders,^28,135,138,161,183^ other GI symptoms,^117^ cardiovascular disease,^120,167^ and bone health.^73^

Few studies examined lifestyle factors, with only one study each examining the relationship between GI symptoms and dietary intakes (tryptophan)^91^ and physical activity.^72^

Included studies assessed biomarkers, including assays of sex hormones (*n* = 3; 2.4%),^31,34,91^ stress hormones (*n* = 2; 1.6%),^31,34^ genetic markers (*n* = 2; 1.6%)^141,142^ and other assays such as urinary tryptophan, melatonin metabolites and serum allopregnalone (*n* = 3, 2.4%).^91,174,180^

## Discussion

This is the first scoping review to systematically map research on GI symptoms in ‘natural’ peri- and postmenopause. The most significant and most unexpected finding was the large volume (*n* = 122) of eligible studies identified, with a notable increase in publications in recent years. However, only 22 studies had a primary objective relating to GI symptoms in menopause. Furthermore, review findings highlight several methodological considerations and evidence gaps of interest to researchers and funders. These relate to the volume and conduct of research, and variables investigated, and are discussed further below. A summary of recommendations for future research is provided in Box 2.

**Box 2.** Recommendations for future research.

Future studies should: • Investigate a range of GI symptoms in diverse sub-populations of individuals experiencing menopause (i.e. different races or ethnicities, and varied geographic locations) • Utilise standardised terminology and clearly report criteria used to determine menopausal stage of participants (e.g. STRAW + 10^6^). • Assess GI symptoms with validated measures, with transparent reporting using appropriate timeframe and tools (e.g. mobile applications or digital diaries) to minimise recall bias and participant burden • Clearly report use of hormone replacement therapies, oral contraceptive use, hysterectomies, medical-induced menopause, providing separate GI symptom data for each group • Utilise longitudinal designs to assess the trajectory of GI symptoms across the menopausal transition and into postmenopause, facilitating comparison of GI symptom experience by menopausal stage • Conduct qualitative research to understand lived experiences of GI symptoms during peri- and postmenopause • Consider a range of correlates of GI symptoms (including psychological health and assays of sex hormones), controlling for potential confounding variables (e.g. age) • Evaluate the effectiveness of interventions for the prevention or management of GI symptoms during peri- and postmenopause

### Volume of evidence

This review mapped studies to at least one of eight GI symptoms,^12^ with constipation being the most investigated symptom. While this review did not summarise study results, conflicting research findings^27,29–31,35^ combined with the large number of studies identified, suggest that constipation is a potential area for synthesis in a systematic review. In contrast, only four studies assessed vomiting in peri- or postmenopause. This may reflect the low prevalence of this symptom, or its omission from menopausal symptom scales commonly used in research.^19–23,69,188–191^ This is in contrast to symptoms such as constipation, diarrhoea, bloating, and abdominal pain, which feature in several instruments.^69,188–191^ Alternatively, symptom reporting may be influenced by an individual’s stereotype of ‘the menopausal woman’, with symptoms only reported that are considered to be linked to the menopause (e.g. hot flushes), rather than all those experienced (i.e. vomiting).^192^ These menopausal stereotypes could vary across cultures, with Im et al.^119^ finding that while nausea and vomiting was documented in 18.9% of a sample of midlife women, reports of symptom frequency and severity varied by ethnicity. Interestingly, a recent study^193^ excluded from this review, found women placed high value on symptom relief from vomiting during menopause. However, it is not clear whether this study included individuals taking HRT, which could be a contributory factor in the aetiology of vomiting.^194^ Further research in diverse populations (i.e. varied ethnicities; HRT users vs non-users) and in consultation with menopausal individuals, may be warranted to determine if vomiting (and other GI symptoms) are indeed related to ‘natural’ menopause.

An important finding is the paucity of studies identified from South American and African countries, compared with Asian, North American and European regions. While this may be partly due to the exclusion of foreign language articles, an assessment of excluded publications indicates only a few non-English language studies were conducted in these regions. With evidence that there is ‘no universal menopausal syndrome’,^195^ and cultural or ethnic differences in menopausal symptom reporting,^196^ it is important that future research is conducted in varied locations to capture diverse experiences across cultures and geographies. This is consistent with findings of the recent Menopause Priority Setting Partnership^197^ which highlighted the need for research to explore menopausal symptoms across countries, cultures and ethnic backgrounds.

### Conduct of studies

This scoping review revealed that qualitative research was notably underrepresented in this area, with only two articles identified.^163,176^ This could be due to a focus in menopausal research on ‘known’ or clinically defined symptoms, rather than women’s actual experience of menopause.^198^ Qualitative research is important for capturing insights into lived experiences at different stages of menopause, the impacts of symptoms (e.g. on quality of life) beyond those on ‘established’ symptom checklists as well as the acceptability of different interventions for symptom management.^199–201^ Furthermore, studies predominantly utilised quantitative cross-sectional designs (*n* = 89), as is consistent with findings of evidence syntheses assessing a diverse range of symptoms in menopausal research.^202–205^ While cross-sectional study designs provide a convenient methodological approach for investigating symptom prevalence, it is difficult to draw causal inferences due to the measurement of exposure (i.e. menopausal stage) and outcome (i.e. GI symptoms) at a single timepoint.^206,207^ Longitudinal studies are needed to assess the trajectory of symptoms across pre-, peri- and postmenopausal stages,^208,209^ to differentiate pre-existing and new onset symptoms^210,211^ and obtain accurate estimates of symptom durations.^212^ However, this review identified only 14 longitudinal studies, and less than half of all included studies made a comparison of GI symptom experience across different menopausal stages. Only three studies compared GI symptoms in early versus late MT, despite evidence of differing menopausal symptomatology across these stages.^6,213^ These findings highlight a need for further longitudinal research to effectively answer questions about the relationship between menopause and GI symptoms, as well as qualitative studies to better understand menopausal lived experiences.

Consistency in use of menopausal terminology and application of validated criteria for the classification of menopausal stages are key to understanding the impact of the MT on symptom experience, and to support comparisons of results from different studies.^61,214^ However, the present review findings indicated that criteria for determining menopausal stage were not reported in over a third of studies, with only 13 studies citing STRAW or updated STRAW + 10 criteria.^6,184^ Failure to document criteria for menopausal staging has been reported in previous research.^61^ An alternative approach using age ranges as a proxy for menopausal stage was applied in nine studies; however, this strategy has been criticised^40^ due to the variability in age of MT and FMP.^215^ To strengthen the reliability and validity of future research, and minimise the risk of misclassification of menopausal stages, studies should utilise and clearly report validated criteria such as STRAW + 10^6^.

Similarly, review findings revealed that many studies did not use a validated tool to assess GI symptoms, with nine studies not reporting the measure used and 47 studies developing a study-specific tool. This issue has been noted in other reviews of menopausal symptoms more widely.^216–218^ While it may be considered necessary to adapt measures for cultural appropriateness, it has been argued that validated tools are required to standardise data collection, and to facilitate rigorous comparisons between studies.^219^ Variation in the timeframe reported for symptom recall was also considerable: from not being described, to immediate capture on an app, or up to 12 months. This too is consistent with findings of evidence syntheses assessing other menopausal symptoms.^216,217,220,221^ Furthermore, 6-to 12-month recall periods could be too lengthy, limiting the accuracy of reporting of menopausal symptoms,^192,222^ whereas daily symptom diaries may be burdensome for study participants.^223^ Future studies should use validated measures to assess GI symptoms, identify appropriate tools (e.g. digital diaries or mobile applications) and timeframes for their recall, and clearly report details of measures used, as is also recommended by the Strengthening the Reporting of Observational Studies in Epidemiology (STROBE) reporting guidance.^224^ This will support the synthesis of findings from multiple studies that could inform policy and practice.

### Variables investigated

The diversity of factors and variables studied suggests there is limited agreement on those thought to be most important in influencing menopausal symptom presentation, as is also described in a scoping review of correlates of palpitations during menopause.^225^ Multiple factors may influence ovarian function, hormonal levels and GI symptom experience, including ageing, dietary patterns, smoking, body mass index, psychological and social variables, and it is argued these should all be considered in menopause research.^117,213,222^ The influence of ageing was only considered in eight included studies, despite evidence demonstrating the prevalence of GI symptoms such as constipation increases with age,^226^ and psychological health was only considered in six studies. Furthermore, evidence gaps were identified, with no included studies assessing the impacts of smoking, sleep disturbances, or gut microbiome composition on GI symptoms in menopause. Interestingly, two ongoing studies^75,77^ are investigating probiotic interventions in menopausal individuals with constipation and diarrhoea, implying that researchers believe there could be a role for changes to gut microbial composition in managing GI and other menopausal symptoms. With menopause occurring at a time of significant biological, psychological and sociocultural change, it is important that future research considers potential confounding variables and correlates to understand their impacts on GI symptoms. This may support the development of targeted lifestyle, behavioural or hormonal interventions for effective GI symptom management in peri- and postmenopause.

A summary of recommendations and methodological considerations for future research on GI symptoms in peri- and postmenopause is outlined in Box 2.

### Strengths and limitations

This review employed comprehensive search methods to identify existing and ongoing research, with a good-practice systematic approach to the selection and mapping of evidence. However, several limitations should be noted. GI symptoms are often not well described in article titles/abstracts, and in consequence, relevant studies may not have been retrieved by database searches. That said, the use of supplementary searches, including citation-searching may have helped mitigate this issue.^227^ With regard to review methods, a second reviewer screened 50% of records, and data charting was completed by a single author, and the potential for bias and human error is noted. Quality assessment of included studies is not a mandatory step in scoping reviews,^70^ and was not completed. While this may limit the review’s capacity to guide policy and practice, the review aim was exploratory, to identify gaps in the literature and inform future research.^70,228^ In addition, due to time limitations and the large number of included studies, research assessing the role of HRT as a cause of, or as an intervention for, GI symptoms was excluded. With evidence that HRT use may influence the presentation of GI symptoms in menopausal women,^62,229–231^ this is a key area that requires investigation in future reviews. As observed in other research on menopausal symptoms,^220,232^ surgical menopause, HRT or oral contraceptive use was often poorly reported, or separate data not provided, making it challenging to identify studies of ‘natural’ menopause. Furthermore, it was often difficult to discern whether symptoms were of GI origin, for example, chest pain was excluded but may have been associated with heartburn. Similarly, abdominal pain may have either a GI or gynaecological aetiology (i.e. menstrual cramping). In order to discern the origin of symptoms, patient-reported outcome measures could gather additional data, for example, about the location of pain using visual tools,^233^ temporal patterns (i.e. after eating, exertion or associated with menstruation), and co-occurring symptoms.^234^ Finally, as the results of studies were not extracted, it is not possible to draw conclusions about the prevalence of GI symptoms in ‘natural’ peri- and postmenopause. This is an important area for future study.

## Conclusion

This review mapped the available evidence on GI symptoms in ‘natural’ peri- and postmenopause to inform future research. While many studies were identified, findings highlighted few longitudinal and qualitative studies, insufficient reporting of criteria used to determine menopausal stage and data collection methods used to assess GI symptoms, and no studies investigating key variables that could influence symptom prevalence. In consequence, this review has identified evidence gaps and methodological considerations with implications for researchers, funders and priority setting partnerships.^10^ Findings may help facilitate the prioritisation of future research and the design of new primary studies to best answer questions about GI symptoms throughout menopause, disentangling the interacting factors that contribute to their lived experience. Evidence syntheses relating to GI symptoms in those taking HRT, and in surgical or medical-induced menopause, would be a valuable addition to the findings presented here.

## Supplemental Material

sj-docx-1-whe-10.1177_17455057251387470 – Supplemental material for The volume and characteristics of research on gastrointestinal symptoms in ‘natural’ peri- and postmenopause: A scoping review Supplemental material, sj-docx-1-whe-10.1177_17455057251387470 for The volume and characteristics of research on gastrointestinal symptoms in ‘natural’ peri- and postmenopause: A scoping review by Naomi Shaw, Rebecca Abbott and Clare Pettinger in Women's Health

sj-docx-2-whe-10.1177_17455057251387470 – Supplemental material for The volume and characteristics of research on gastrointestinal symptoms in ‘natural’ peri- and postmenopause: A scoping review Supplemental material, sj-docx-2-whe-10.1177_17455057251387470 for The volume and characteristics of research on gastrointestinal symptoms in ‘natural’ peri- and postmenopause: A scoping review by Naomi Shaw, Rebecca Abbott and Clare Pettinger in Women's Health

sj-docx-3-whe-10.1177_17455057251387470 – Supplemental material for The volume and characteristics of research on gastrointestinal symptoms in ‘natural’ peri- and postmenopause: A scoping review Supplemental material, sj-docx-3-whe-10.1177_17455057251387470 for The volume and characteristics of research on gastrointestinal symptoms in ‘natural’ peri- and postmenopause: A scoping review by Naomi Shaw, Rebecca Abbott and Clare Pettinger in Women's Health

sj-docx-4-whe-10.1177_17455057251387470 – Supplemental material for The volume and characteristics of research on gastrointestinal symptoms in ‘natural’ peri- and postmenopause: A scoping review Supplemental material, sj-docx-4-whe-10.1177_17455057251387470 for The volume and characteristics of research on gastrointestinal symptoms in ‘natural’ peri- and postmenopause: A scoping review by Naomi Shaw, Rebecca Abbott and Clare Pettinger in Women's Health

sj-docx-5-whe-10.1177_17455057251387470 – Supplemental material for The volume and characteristics of research on gastrointestinal symptoms in ‘natural’ peri- and postmenopause: A scoping review Supplemental material, sj-docx-5-whe-10.1177_17455057251387470 for The volume and characteristics of research on gastrointestinal symptoms in ‘natural’ peri- and postmenopause: A scoping review by Naomi Shaw, Rebecca Abbott and Clare Pettinger in Women's Health

## References

[1] Harper JC Phillips S Biswakarma R , et al. An online survey of perimenopausal women to determine their attitudes and knowledge of the menopause. Womens Health 2022; 18: 1–18. 10.1177/17455057221106890 PMC9244939 35758176

[2] British Menopause Society. National survey: the results, https://thebms.org.uk/wp-content/uploads/2023/01/BMS-Infographics-JANUARY-2023-NationalSurveyResults.pdf (2022, accessed 12 November 2023).

[3] World Health Organization. Menopause. Factsheet, https://www.who.int/news-room/fact-sheets/detail/menopause (2022, accessed 29 July 2024).

[4] Laisk T Tšuiko O Jatsenko T , et al. Demographic and evolutionary trends in ovarian function and aging. Hum Reprod Update 2019; 25: 34–50. 30346539 10.1093/humupd/dmy031

[5] Fraser A Johnman C Whitley E , et al. The evolutionary ecology of age at natural menopause: implications for public health. Evol Hum Sci 2020; 2: e57. 10.1017/ehs.2020.59 PMC7612003 34796315

[6] Harlow SD Gass M Hall JE , et al. Executive summary of the Stages of Reproductive Aging Workshop + 10: addressing the unfinished agenda of staging reproductive aging. Menopause 2012; 19: 387–395. 22343510 10.1097/gme.0b013e31824d8f40 PMC3340903

[7] Monteleone P Mascagni G Giannini A , et al. Symptoms of menopause - global prevalence, physiology and implications. Nat Rev Endocrinol 2018; 14: 199–215. 29393299 10.1038/nrendo.2017.180

[8] Santoro N Roeca C Peters BA , et al. The menopause transition: signs, symptoms, and management options. J Clin Endocrinol Metab 2021; 106: 1–15. 33095879 10.1210/clinem/dgaa764

[9] Brewis J Beck V Davies A , et al. The effects of menopause transition on women’s economic participation in the UK, https://www.gov.uk/government/publications/menopause-transition-effects-on-womens-economic-participation (2017, accessed 12 December 2023).

[10] Menopause Priority Setting Partnership. Protocol Version 1.1, https://obgyn.uchicago.edu/research/menopause-priority-setting-partnership (2023, accessed 28 November 2023).

[11] Department of Health and Social Care. Women’s Health Strategy for England, https://www.gov.uk/government/publications/womens-health-strategy-for-england/womens-health-strategy-for-england (2022, accessed 4 November 2023).

[12] Spiegel BMR Hays RD Bolus R , et al. Development of the NIH Patient-Reported Outcomes Measurement Information System (PROMIS) gastrointestinal symptom scales. Am J Gastroenterol 2014; 109: 1804–1811. 25199473 10.1038/ajg.2014.237 PMC4285435

[13] Almario CV Ballal ML Chey WD , et al. Burden of gastrointestinal symptoms in the United States: results of a nationally representative survey of over 71,000 Americans. Am J Gastroenterol 2018; 113: 1701–1710. 30323268 10.1038/s41395-018-0256-8 PMC6453579

[14] Bosman M Weerts Z Snijkers JTW , et al. The socioeconomic impact of irritable bowel syndrome: an analysis of direct and indirect health care costs. Clin Gastroenterol Hepatol 2023; 21: 2660–2669. 36731587 10.1016/j.cgh.2023.01.017

[15] Denby N. Digestive health and the menopause – is it a thing?, https://professionals.symprove.com/blogs/symprove-for-professionals/digestive-health-and-the-menopause-is-it-a-thing (2023, accessed 12 December 2023).

[16] Valdesolo F. One terrible, no-good (and often overlooked) side effect of menopause, https://www.vogue.co.uk/article/menoapuse-gut-issues (2023, accessed 12 December 2023).

[17] North American Menopause Society. The 2023 nonhormone therapy position statement of the North American Menopause Society. Menopause 2023; 30: 573–590. 37252752 10.1097/GME.0000000000002200

[18] National Institute for Health and Care Excellence. Menopause: diagnosis and management (Clinical guideline [NG23]), https://www.nice.org.uk/guidance/ng23 (2024, accessed 25 November 2024). 33141539

[19] Heinemann LA DoMinh T Strelow F , et al. The Menopause Rating Scale (MRS) as outcome measure for hormone treatment? A validation study. Health Qual Life Outcomes 2004; 2: 67. 15555079 10.1186/1477-7525-2-67 PMC534786

[20] Greene JG. A factor analytic study of climacteric symptoms. J Psychosom Res 1976; 20: 425–430. 1003364 10.1016/0022-3999(76)90005-2

[21] Blatt MHG Wiesbader H Kupperman HS. Vitamin E and climacteric syndrome: failure of effective control as measured by menopausal index. Arch Intern Med 1953; 91: 792–799. 10.1001/archinte.1953.00240180101012 13050216

[22] Holte A Mikkelsen A. The menopausal syndrome: a factor analytic replication. Maturitas 1991; 13: 193–203. 1943827 10.1016/0378-5122(91)90194-u

[23] Pérez-López FR Fernández-Alonso AM Pérez-Roncero G , et al. Assessment of menopause-related symptoms in mid-aged women with the 10-item Cervantes Scale. Maturitas 2013; 76: 151–154. 23916081 10.1016/j.maturitas.2013.07.002

[24] Lenell C Pena-Chavez R Burdick RJ , et al. The relationship between menopause and dysphagia: a scoping review. Womens Health Reports 2022; 3: 990–997. 10.1089/whr.2022.0078 PMC9811845 36636319

[25] Adeyemo MA Spiegel BM Chang L. Meta-analysis: do irritable bowel syndrome symptoms vary between men and women? Aliment Pharmacol Ther 2010; 32: 738–755. 20662786 10.1111/j.1365-2036.2010.04409.x PMC2932820

[26] National Institute for Health and Care Excellence. Irritable bowel syndrome in adults: diagnosis and management (Clinical guideline [CG61]), https://www.nice.org.uk/guidance/cg61 (2017, accessed 10 December 2023). 31825575

[27] Geeta K Khanna K Mahna R. Physical health and morbidity profile of women in reproductive and post menopausal years. Indian J Nutr Diet 2011; 48: 490–498.

[28] Selcuk S Cam C Asoglu MR , et al. The effect of concealed concomitant anal incontinence symptoms in patients with urinary incontinence on their quality of life. Int Urogynecol J 2012; 23: 1781–1784. 22584923 10.1007/s00192-012-1808-x

[29] Chen LJ Burr R Cain K , et al. Age differences in upper gastrointestinal symptoms and vagal modulation in women with irritable bowel syndrome. Biol Res Nurs 2024; 26: 46–55. 37353474 10.1177/10998004231186188 PMC10850873

[30] Huerta-Franco MR Vargas-Luna M Somoza X , et al. Gastric responses to acute psychological stress in climacteric women: a pilot study. Menopause 2019; 26: 469–475. 30586006 10.1097/GME.0000000000001274

[31] Callan NGL Mitchell ES Heitkemper MM , et al. Constipation and diarrhea during the menopause transition and early postmenopause: observations from the Seattle Midlife Women’s Health Study. Menopause 2018; 25: 615–624. 29381667 10.1097/GME.0000000000001057 PMC8080720

[32] Bezerra LRPS Neto JAV Vasconcelos CTM , et al. Prevalence of unreported bowel symptoms in women with pelvic floor dysfunction and the impact on their quality of life. Int Urogynecol J 2014; 25: 927–933. 24562788 10.1007/s00192-013-2317-2

[33] Choghakhori R Abbasnezhad A Amani R , et al. Sex-related differences in clinical symptoms, quality of life, and biochemical factors in irritable bowel syndrome. Dig Dis Sci 2017; 62: 1550–1560. 28374085 10.1007/s10620-017-4554-6

[34] Callan NGL Mitchell ES Heitkemper MM , et al. Abdominal pain during the menopause transition and early postmenopause: observations from the Seattle Midlife Women’s Health Study. Womens Midlife Health 2019; 5: 2. 31388434 10.1186/s40695-019-0046-5 PMC6679532

[35] Huang L Jiang H Zhu M , et al. Prevalence and risk factors of chronic constipation among women aged 50 years and older in Shanghai, China. Med Sci Monit 2017; 23: 2660–2667. 28562581 10.12659/MSM.904040 PMC5462481

[36] Crandall CJ Mehta JM Manson JE. Management of menopausal symptoms: a review. JAMA 2023; 329: 405–420. 36749328 10.1001/jama.2022.24140

[37] Nie X Xie R Tuo B. Effects of estrogen on the gastrointestinal tract. Dig Dis Sci 2018; 63: 583–596. 29387989 10.1007/s10620-018-4939-1

[38] Jiang Y Greenwood-Van Meerveld B Johnson AC , et al. Role of estrogen and stress on the brain-gut axis. Am J Physiol Gastrointest Liver Physiol 2019; 317: G203–G209. 10.1152/ajpgi.00144.2019 PMC6734369 31241977

[39] Coquoz A Regli D Stute P. Impact of progesterone on the gastrointestinal tract: a comprehensive literature review. Climacteric 2022; 25: 337–361. 35253565 10.1080/13697137.2022.2033203

[40] Heitkemper MM Chang L. Do fluctuations in ovarian hormones affect gastrointestinal symptoms in women with irritable bowel syndrome? Gend Med 2009; 6: 152–167. 19406367 10.1016/j.genm.2009.03.004 PMC3322543

[41] Zia JK Heitkemper MM. Upper gastrointestinal tract motility disorders in women, gastroparesis, and gastroesophageal reflux disease. Gastroenterol Clin North Am 2016; 45: 239–251. 27261896 10.1016/j.gtc.2016.02.003

[42] Shieh A Epeldegui M Karlamangla AS , et al. Gut permeability, inflammation, and bone density across the menopause transition. JCI Insight 2020; 5: 30. 10.1172/jci.insight.134092 PMC7098720 31830000

[43] Yoon K Kim N. Roles of sex hormones and gender in the gut microbiota. J Neurogastroenterol Motil 2021; 27: 314–325. 33762473 10.5056/jnm20208 PMC8266488

[44] d’Afflitto M Upadhyaya A Green A , et al. Association between sex hormone levels and gut microbiota composition and diversity-a systematic review. J Clin Gastroenterol 2022; 56: 384–392. 35283442 10.1097/MCG.0000000000001676 PMC7612624

[45] Collins SM. A role for the gut microbiota in IBS. Nat Rev Gastroenterol Hepatol 2014; 11: 497–505. 24751910 10.1038/nrgastro.2014.40

[46] Drossman DA. Functional gastrointestinal disorders: history, pathophysiology, clinical features, and Rome IV. Gastroenterology 2016; 150: 1262–1279.e1262. 10.1053/j.gastro.2016.02.032 27144617

[47] Khan I Ullah N Zha L , et al. Alteration of gut microbiota in Inflammatory Bowel Disease (IBD): cause or consequence? IBD treatment targeting the gut microbiome. Pathogens 2019; 8: 126. 31412603 10.3390/pathogens8030126 PMC6789542

[48] Yang PL Heitkemper MM Kamp KJ. Irritable bowel syndrome in midlife women: a narrative review. Womens Midlife Health 2021; 7: 4. 34059117 10.1186/s40695-021-00064-5 PMC8166071

[49] Thomas AJ Mitchell ES Woods NF. Undesirable stressful life events, impact, and correlates during midlife: observations from the Seattle Midlife Women’s Health study. Womens Midlife Health 2019; 5: 1. 30766725 10.1186/s40695-018-0045-y PMC6318955

[50] Meleine M Matricon J. Gender-related differences in irritable bowel syndrome: potential mechanisms of sex hormones. World J Gastroenterol 2014; 20: 6725–6743. 24944465 10.3748/wjg.v20.i22.6725 PMC4051914

[51] Mulak A Taché Y Larauche M. Sex hormones in the modulation of irritable bowel syndrome. World J Gastroenterol 2014; 20: 2433–2448. 24627581 10.3748/wjg.v20.i10.2433 PMC3949254

[52] Hogan AM Collins D Baird AW , et al. Estrogen and its role in gastrointestinal health and disease. Int J Colorectal Dis 2009; 24: 1367–1375. 19655153 10.1007/s00384-009-0785-0

[53] Dare JS. Transitions in midlife women’s lives: contemporary experiences. Health Care Women Int 2011; 32: 111–133. 21229427 10.1080/07399332.2010.500753

[54] Mirin AA. Gender disparity in the funding of diseases by the U.S. National Institutes of Health. J Womens Health 2021; 30: 956–963. 10.1089/jwh.2020.8682 PMC8290307 33232627

[55] Orgad S Rottenberg C. The menopause moment: the rising visibility of ‘the change’ in UK news coverage. Eur J Cult Stud 2023; 27: 519–539.

[56] Munn Z Peters MDJ Stern C , et al. Systematic review or scoping review? Guidance for authors when choosing between a systematic or scoping review approach. BMC Med Res Methodol 2018; 18: 143. 30453902 10.1186/s12874-018-0611-x PMC6245623

[57] Peters MDJ Godfrey C McInerney P , et al. Chapter 10: scoping reviews. JBI manual for evidence synthesis, https://jbi-global-wiki.refined.site/space/MANUAL/355862497/10.+Scoping+reviews (2024, accessed 10 November 2024).

[58] Pollock D Peters MDJ Khalil H , et al. Recommendations for the extraction, analysis, and presentation of results in scoping reviews. JBI Evid Synth 2023; 21: 520–532. 36081365 10.11124/JBIES-22-00123

[59] Tricco AC Lillie E Zarin W , et al. PRISMA Extension for Scoping Reviews (PRISMA-ScR): checklist and explanation. Ann Intern Med 2018; 169: 467–473. 30178033 10.7326/M18-0850

[60] British Menopause Society. Surgical menopause: a toolkit for healthcare professionals, https://thebms.org.uk/wp-content/uploads/2021/06/13-BMS-TfC-Surgical-Menopause-JUNE202102B.pdf (2021, accessed 11 December 2023).

[61] Ambikairajah A Walsh E Cherbuin N. A review of menopause nomenclature. Reprod Health 2022; 19: 29. 35101087 10.1186/s12978-022-01336-7 PMC8805414

[62] Aldhaleei WA Bhagavathula AS Wallace MB , et al. The association between menopausal hormone therapy and gastroesophageal reflux disease: a systematic review and meta-analysis. Menopause 2023; 30: 867–872. 37369078 10.1097/GME.0000000000002214

[63] Higgins JPT Thomas J Chandler J , et al. Cochrane handbook for systematic reviews of interventions, version 6.4 (updated August 2023), https://www.training.cochrane.org/handbook (2023, accessed 10 November 2023).

[64] Pieper D Puljak L. Language restrictions in systematic reviews should not be imposed in the search strategy but in the eligibility criteria if necessary. J Clin Epidemiol 2021; 132: 146–147. 33383129 10.1016/j.jclinepi.2020.12.027

[65] Ross-White A Lieggi M Palacio FBL , et al. Search methodology for JBI Evidence Syntheses. Methodological considerations. JBI Manual for Evidence Synthesis, https://jbi-global-wiki.refined.site/space/MANUAL/355827873/2.4+Search+Methodology+for+JBI+Evidence+Syntheses (2024, accessed 3 January 2025).

[66] Haddaway NR Grainger MJ Gray CT. Citationchaser: a tool for transparent and efficient forward and backward citation chasing in systematic searching. Res Synth Methods 2022; 13: 533–545. 35472127 10.1002/jrsm.1563

[67] Ouzzani M Hammady H Fedorowicz Z , et al. Rayyan—a web and mobile app for systematic reviews. Syst Rev 2016; 5: 210. 27919275 10.1186/s13643-016-0384-4 PMC5139140

[68] Waffenschmidt S Knelangen M Sieben W , et al. Single screening versus conventional double screening for study selection in systematic reviews: a methodological systematic review. BMC Med Res Methodol 2019; 19: 132. 31253092 10.1186/s12874-019-0782-0 PMC6599339

[69] Hilditch JR Lewis J Peter A , et al. A menopause-specific quality of life questionnaire: development and psychometric properties. Maturitas 1996; 24: 161–175. 8844630 10.1016/s0378-5122(96)82006-8

[70] Khalil H Peters MDJ Tricco AC , et al. Conducting high quality scoping reviews-challenges and solutions. J Clin Epidemiol 2021; 130: 156–160. 33122034 10.1016/j.jclinepi.2020.10.009

[71] OurWorldinData. Definitions of world regions, https://ourworldindata.org/world-region-map-definitions (2018, accessed 4 July 2024).

[72] Luque MD. Physical activity and quality of life through the menopausal transition. PhD Thesis, Trident University International, USA, 2012.

[73] Wang H Jiang Q Yan J , et al. Gastrointestinal health and serum proteins are associated with BMD in postmenopausal women: a cross-sectional study. Nutr Metab (Lond) 2024; 21(1): 86. 39506776 10.1186/s12986-024-00865-1 PMC11539781

[74] Aras SG Grant AD Konhilas JP. Clustering of >145,000 symptom logs reveals distinct pre, peri, and post menopausal phenotypes. medRxiv 2023; 14: 1–27. DOI: 10.1101/2023.12.12.23299821. PMC11699220 39753725

[75] Maneuver Marketing. A randomized-controlled trial to examine the effects of a daily probiotic supplement on common symptoms of menopause, https://clinicaltrials.gov/study/NCT06148714 (2024, accessed 2 July 2024).

[76] Suman S. Ayurvedic treatment for symptoms which occur around the time of actual menopause, https://trialsearch.who.int/Trial2.aspx?TrialID=CTRI/2023/09/057443 (2023, accessed 2 July 2024).

[77] Yoneda S. Examination study of the effect of lactic acid bacteria on the imbalance and discomfort of mental and physical conditions in healthy peri/post-menopause females, https://trialsearch.who.int/Trial2.aspx?TrialID=JPRN-UMIN000045547 (2022, accessed 2 July 2024).

[78] Abraham S Llewellyn-Jones D Perz J. Changes in Australian women’s perception of the menopause and menopausal symptoms before and after the climacteric. Maturitas 1994; 20: 121–128. 7715463 10.1016/0378-5122(94)90007-8

[79] Adeniji AO Ogunleye EA Olawuyi YO. Nutritional status and menopausal complications among adult women in Ogbomoso, Southwest, Nigeria. Afr J Biomed Res 2023; 26: 53–58.

[80] Agreus L Svardsudd K Nyren O , et al. The epidemiology of abdominal symptoms - prevalence and demographic characteristics in a Swedish adult-population - a report from the abdominal symptom study. Scand J Gastroenterol 1994; 29: 102–109. 8171277 10.3109/00365529409090447

[81] Al-Badr A Saleem Z Kaddour O , et al. Prevalence of pelvic floor dysfunction: a Saudi national survey. BMC Womens Health 2022; 22: 27. 35120501 10.1186/s12905-022-01609-0 PMC8815131

[82] Ali Ismail AM Saad AE Fouad Abd-Elrahman NA , et al. Effect of Benson’s relaxation therapy alone or combined with aerobic exercise on cortisol, sleeping quality, estrogen, and severity of dyspeptic symptoms in perimenopausal women with functional dyspepsia. Eur Rev Med Pharmacol Sci 2022; 26: 8342–8350. 36459044 10.26355/eurrev_202211_30367

[83] Alshiek J Jalalizadeh M Wei Q , et al. Ultrasongraphic age-related changes of the pelvic floor muscles in nulliparous women and their association with pelvic floor symptoms: a pilot study. Neurourol Urodyn 2019; 38: 1305–1312. 30927314 10.1002/nau.23979

[84] Alwi SARS Lee PY Awi I , et al. The menopausal experience among indigenous women of Sarawak, Malaysia. Climacteric 2009; 12: 548–556. 19905907 10.3109/13697130902919519

[85] Ayranci U Orsal O Orsal O , et al. Menopause status and attitudes in a Turkish midlife female population: an epidemiological study. BMC Womens Health 2010; 10: 1–14. 20064263 10.1186/1472-6874-10-1 PMC2822813

[86] Bapayeva G Terzic M Semenova Y , et al. Unveiling the role of the work environment in the quality of life of menopausal physicians and nurses. Int J Environ Res Public Health 2023; 20: 12. 10.3390/ijerph20186744 PMC10531365 37754604

[87] Barati M Akbari-Heidari H Samadi-Yaghin E , et al. The factors associated with the quality of life among postmenopausal women. BMC Womens Health 2021; 21: 208–208. 34006264 10.1186/s12905-021-01361-x PMC8130393

[88] Barbosa M Glavind-Kristensen M Soerensen MM , et al. Secondary sphincter repair for anal incontinence following obstetric sphincter injury: functional outcome and quality of life at 18 years of follow-up. Colorectal Dis 2019; 22: 71–79. 31347749 10.1111/codi.14792

[89] Belcaro G Cesarone MR Cornelli U , et al. Afragil^®^ in the treatment of 34 menopause symptoms: a pilot study. Panminerva Med 2010; 52: 49–54. 20657535

[90] Bener A Saleh N Burgut FT. Prevalence and determinants of fecal incontinence in premenopausal women in an Arabian community. Climacteric 2008; 11: 429–435. 18781489 10.1080/13697130802241519

[91] Blonska A Chojnacki M Macieja A , et al. Tryptophan metabolism in postmenopausal women with functional constipation. Int J Mol Sci 2023; 25: 24. 38203444 10.3390/ijms25010273 PMC10778582

[92] Brimiene I Siaudinyte M Burokas A , et al. Exploration of the association between menopausal symptoms, gastrointestinal symptoms, and perceived stress: survey-based analysis. Menopause 2023; 30: 1124–1131. 37788428 10.1097/GME.0000000000002259

[93] Brown JP Gallicchio L Flaws JA , et al. Relations among menopausal symptoms, sleep disturbance and depressive symptoms in midlife. Maturitas 2009; 62: 184–189. 19128903 10.1016/j.maturitas.2008.11.019 PMC2693910

[94] Brown WJ Mishra GD Dobson AJ. Changes in physical symptoms during the menopause transition. Int J Behav Med 2002; 9: 53–67. 12112996 10.1207/s15327558ijbm0901_04

[95] Chang L Lee OY Naliboff BD , et al. Sensation of bloating and visible abdominal distension in patients with irritable bowel syndrome. Am J Gastroenterol 2001; 96: 3341–3347. 11774947 10.1111/j.1572-0241.2001.05336.x

[96] Chen GD Hu SW Chen YC , et al. Prevalence and correlations of anal incontinence and constipation in Taiwanese women. Neurourol Urodyn 2003; 22: 664–669. 14595611 10.1002/nau.10067

[97] Chojnacki C Medrek-Socha M Konrad P , et al. The value of melatonin supplementation in postmenopausal women with Helicobacter pylori-associated dyspepsia. BMC Womens Health 2020; 20: 262. 33243209 10.1186/s12905-020-01117-z PMC7691069

[98] Chojnacki C Walecka-Kapica E Lokiec K , et al. Influence of melatonin on symptoms of irritable bowel syndrome in postmenopausal women. Endokrynol Pol 2013; 64: 114–120. 23653274

[99] Chung SH Kim TH Lee HH , et al. Premenstrual syndrome and premenstrual dysphoric disorder in perimenopausal women. J Menopausal Med 2014; 20: 69–74. 25371896 10.6118/jmm.2014.20.2.69 PMC4207004

[100] Coslov N Richardson MK Woods NF. Symptom experience during the late reproductive stage and the menopausal transition: observations from the Women Living Better survey. Menopause 2021; 28: 1012–1025. 34313615 10.1097/GME.0000000000001805 PMC8549458

[101] Coslov N Richardson MK Woods NF. “Not feeling like myself” in perimenopause—what does it mean? Observations from the women living better survey. Menopause 2023; 31(5): 390–398. 10.1097/GME.0000000000002339 PMC11465791 38531011

[102] Deeks A Zoungas S Teede H. Risk perception in women: a focus on menopause. Menopause 2008; 15: 304–309. 18090036 10.1097/gme.0b013e31812f7b65

[103] Dennerstein L Dudley EC Guthrie JR. Predictors of declining self-rated health during the transition to menopause. J Psychosom Res 2003; 54: 147–153. 12573736 10.1016/s0022-3999(02)00415-4

[104] Dennerstein L Smith AMA Morse C , et al. Menopausal symptoms in Australian women. Med J Aust 1993; 159: 232–236. 8412889 10.5694/j.1326-5377.1993.tb137821.x

[105] Duffy OK Iversen L Hannaford PC. Factors associated with reporting classic menopausal symptoms differ. Climacteric 2013; 16: 240–251. 22992029 10.3109/13697137.2012.697227

[106] Eliasvandi P Khodaie L Mohammad Alizadeh Charandabi S , et al. Effect of an herbal capsule on chronic constipation among menopausal women: a randomized controlled clinical trial. Avicenna J Phytomed 2019; 9: 517–529. 31763211 10.22038/AJP.2019.13109 PMC6823525

[107] Fisher WI Thurston RC. Measuring hot flash phenomenonology using ambulatory prospective digital diaries. Menopause 2016; 23: 1222–1227. 27404030 10.1097/GME.0000000000000685 PMC5079773

[108] Fukuda S Matsuzaka M Takahashi P , et al. Bowel habits before and during menses in Japanese women of climacteric age: a population based study. Tohoku J Exp Med 2005; 206: 99–104. 15888965 10.1620/tjem.206.99

[109] Hajdini M Osmani V. Prevalence of symptoms and attitudes towards menopause in midlife female population in Albania. Int J Ecosyst Ecol Sci 2017; 7: 613–618.

[110] Hakimi S Aminian E Mohammadi M , et al. Prevalence and risk factors of urinary/anal incontinence and pelvic organ prolapse in healthy middle-aged Iranian women. J Menopausal Med 2020; 26: 24–28. 32307947 10.6118/jmm.19201 PMC7160590

[111] High RV Marcellino PA. Menopausal women and the work environment. Soc Behav Pers 1994; 22: 347–353.

[112] Hilditch JR Chen S Norton PG , et al. Experience of menopausal symptoms by Chinese and Canadian women. Climacteric 1999; 2: 164–173. 11910593 10.3109/13697139909038058

[113] Huerta R Mena A Malacara JM , et al. Symptoms at perimenopausal period: its association with attitudes toward sexuality, life-style, family function, and FSH levels. Psychoneuroendocrinology 1995; 20: 135–148. 7899534 10.1016/0306-4530(94)00046-d

[114] Huerta R Mena A Malacara JM , et al. Symptoms at the menopausal and premenopausal years: their relationship with insulin, glucose, cortisol, FSH, prolactin, obesity and attitudes towards sexuality. Psychoneuroendocrinology 1995; 20: 851–864. 8834092 10.1016/0306-4530(95)00030-5

[115] Im EO Lee B Chee W , et al. Menopausal symptoms among four major ethnic groups in the United States. West J Nurs Res 2010; 32: 540–565. 20685910 10.1177/0193945909354343 PMC3033753

[116] Im EO Lee SH Chee W. Subethnic differences in the menopausal symptom experience of Asian American midlife women. J Transcult Nurs 2010; 21: 123–133. 20220032 10.1177/1043659609357639 PMC2838208

[117] Im EO Choi MY Jin R , et al. Cluster analysis on gastrointestinal symptoms during menopausal transition. West J Nurs Res 2023; 45: 133–143. 35801285 10.1177/01939459221109810 PMC13573981

[118] Im EO Choi MY Kim G , et al. Immigration transition and gastrointestinal symptoms during menopausal transition: midlife women in the US. Menopause 2022; 29: 840–849. 35796555 10.1097/GME.0000000000001989 PMC9273015

[119] Im EO Kim G Choi M , et al. Gastrointestinal symptoms in four major racial/ethnic groups of midlife women: race/ethnicity and menopausal status. Menopause 2021; 29: 156–163. 34873105 10.1097/GME.0000000000001898 PMC8795485

[120] Im EO Ko Y Chee E , et al. Midlife women’s cardiovascular symptoms: a cluster analysis. Health Care Women Int 2017; 38: 1275–1288. 28532290 10.1080/07399332.2017.1332626 PMC6095467

[121] Jia M Kluwe L Liu HC , et al. Efficacy and side-effects of a semi-individualized Chinese herb mixture “Tiao Geng Tang” for menopausal syndrome in China. In Vivo 2015; 29: 109–115. 25600538

[122] Joh HK Seong MK Oh SW. Fecal incontinence in elderly Koreans. J Am Geriatr Soc 2009; 58: 116–121. 20002514 10.1111/j.1532-5415.2009.02613.x

[123] Joshi M Nair S. Epidemiological study to assess the menopausal problems during menopausal transition in middle age women of Vadodara, Gujarat, India. Indian J Obstet Gynecol Res 2015; 2: 163–168.

[124] Kang HK Kaur A Dhiman A. Menopause-specific quality of life of rural women. Indian J Community Med 2021; 46: 273–276. 34321740 10.4103/ijcm.IJCM_665_20 PMC8281871

[125] Kargin S Cifci S Kaynak A , et al. The relationship between fecal incontinence and vaginal delivery in the postmenopausal stage. Turk J Obstet Gynecol 2017; 14: 37–44. 28913133 10.4274/tjod.56650 PMC5558316

[126] Karmakar N Majumdar S Dasgupta A , et al. Quality of life among menopausal women: a community-based study in a rural area of West Bengal. J Midlife Health 2017; 8: 21–27. 28458476 10.4103/jmh.JMH_78_16 PMC5367220

[127] Kaur M. Post-menopausal symptoms: reports from urban women. Indian J Contin Nurs Educ 2021; 22: 53–56.

[128] Kirka AS Oksuz SK Gul DK , et al. Constipation and its effects on quality of life in menopausal women. Int J Caring Sci 2021; 14: 270–275.

[129] Koirala D Thapa N Shrestha S. Quality of life of postmenopausal women of Kaski district. Nepal J Obstet Gynaecol 2020; 15: 43–49.

[130] Koo S Ahn Y Lim J-Y , et al. Obesity associates with vasomotor symptoms in postmenopause but with physical symptoms in perimenopause: a cross-sectional study. BMC Womens Health 2017; 17: 1–8. 29216853 10.1186/s12905-017-0487-7 PMC5721621

[131] Kumar SK Mrinal NR. The effects of dietetic therapies on stress, waist measurements, triglyceride levels, diarrhea and constipation with reference to menopause. Indian J Health Wellbeing 2017; 8: 972–976.

[132] Lee OY Mayer EA Schmulson M , et al. Gender-related differences in IBS symptoms. Am J Gastroenterol 2001; 96: 2184–2193. 11467651 10.1111/j.1572-0241.2001.03961.x

[133] Li RX Ma M Xiao XR , et al. Perimenopausal syndrome and mood disorders in perimenopause: prevalence, severity, relationships, and risk factors. Medicine 2016; 95: e4466. 10.1097/MD.0000000000004466 PMC4985318 27512863

[134] Li S Holm K Gulanick M , et al. Perimenopause and the quality of life. Clin Nurs Res 2000; 9: 6–23. 11271048 10.1177/105477380000900102

[135] Li T Zhang YJ Zhang HL , et al. Prevalence and risk factors of stress urinary incontinence among perimenopausal women and its influence on daily life in women with sexual desire problem. Curr Med Sci 2019; 39: 615–621. 31346999 10.1007/s11596-019-2082-7

[136] Lock M Kaufert PA Gilbert P. Cultural construction of the menopausal syndrome: the Japanese case. Maturitas 1988; 10: 317–332. 3265758 10.1016/0378-5122(88)90067-9

[137] Loutfy I Aziz FA Dabbous NI , et al. Women’s perception and experience of menopause: a community-based study in Alexandria, Egypt. East Mediterr Health Journal 2006; 12: S93–S106. 17361681

[138] Lu S Zhang HL Zhang YJ , et al. Prevalence and risk factors of urinary incontinence among perimenopausal women in Wuhan. J Huazhong Univ Sci Technolog Med Sci 2016; 36: 723–726. 27752911 10.1007/s11596-016-1651-2

[139] Lucas EA Hammond LJ Mocanu V , et al. Daily consumption of dried plum by postmenopausal women does not cause undesirable changes in bowel function. J Appl Res 2004; 4: 37–43.

[140] Luciano L Bouvier M Baumstarck K , et al. Is the extent of obstetric anal sphincter injury correlated with the severity of fecal incontinence in the long term? Tech Coloproctol 2020; 24: 49–55. 31820190 10.1007/s10151-019-02128-1

[141] Luptáková L Siváková D Cernanová V , et al. Menopausal complaints in Slovak midlife women and the impact of CYP1B1 polymorphism on their incidence. Anthropol Anz 2012; 69: 399–415. 23350153

[142] Luptakova L Sivakova D Sramekova D , et al. The association of cytochrome P450 1B1 Leu432Val polymorphism with biological markers of health and menopausal symptoms in Slovak midlife women. Menopause 2012; 19: 216–224. 22011756 10.1097/gme.0b013e3182281b54

[143] Lyndaker C Hulton L. The influence of age on symptoms of perimenopause. J Obstet Gynecol Neonatal Nurs 2004; 33: 340–347. 10.1177/0884217504264872 15180197

[144] Ma M Li RX Xiao XR , et al. A health survey of perimenopausal syndrome and mood disorders in perimenopause: a cross-sectional study in Shanghai. Int J Clin Exp Med 2017; 10: 12382–12403.

[145] Ma W Li Y Heianza Y , et al. Associations of bowel movement frequency with risk of cardiovascular disease and mortality among US women. Sci Rep 2016; 6: 33005. 27596972 10.1038/srep33005 PMC5011651

[146] Mahajan N Aggarwal M Bagga A. Health issues of menopausal women in North India. J Midlife Health 2012; 3: 84–87. 23372325 10.4103/0976-7800.104467 PMC3555032

[147] Maiello M Zito A Cecere A , et al. Chest pain and palpitations in postmenopausal women with mitral valve prolapse: is there a gastro-oesophageal origin? Intern Med J 2022; 52: 848–852. 33347741 10.1111/imj.15174

[148] Manonai J Wattanayingcharoenchai R Sarit-Apirak S , et al. Prevalence and risk factors of anorectal dysfunction in women with urinary incontinence. Arch Gynecol Obstet 2009; 281: 1003–1007. 19756674 10.1007/s00404-009-1223-9

[149] Markland AD Jelovsek JE Rahn DD , et al. Irritable bowel syndrome and quality of life in women with fecal incontinence. Urogynecology 2017; 23: 179–183. 10.1097/SPV.0000000000000358 PMC5404998 27918339

[150] Martinez-Vazquez S Hernandez-Martinez A Peinado-Molina RA , et al. Impact of overweight and obesity in postmenopausal women. Climacteric 2023; 26: 577–582. 37477988 10.1080/13697137.2023.2228692

[151] Meriggiola MC Nanni M Bachiocco V , et al. Menopause affects pain depending on pain type and characteristics. Menopause 2012; 19: 517–523. 22334057 10.1097/gme.0b013e318240fe3d

[152] Methot J Hamelin BA Bogaty P , et al. Does hormonal status influence the clinical presentation of acute coronary syndromes in women? J Womens Health 2004; 13: 695–702. 10.1089/jwh.2004.13.695 15333284

[153] Mikhail BI Ragheb MS. Health-related concerns and experiences of employed perimenopausal women in Alexandria, Egypt. Health Care Women Int 1996; 17: 173–186. 8852219 10.1080/07399339609516231

[154] Mishra G Lee C Brown W , et al. Menopausal transitions, symptoms and country of birth: the Australian Longitudinal Study on Women’s Health. Aust N Z J Public Health 2002; 26: 563–570. 12530802 10.1111/j.1467-842x.2002.tb00367.x

[155] Mishra GD Dobson AJ. Using longitudinal profiles to characterize women’s symptoms through midlife: results from a large prospective study. Menopause 2012; 19: 549–555. 22198658 10.1097/gme.0b013e3182358d7c

[156] Morse CA Dudley E Guthrie J , et al. Relationships between premenstrual complaints and perimenopausal experiences. J Psychosom Obstet Gynaecol 1998; 19: 182–191. 9929844 10.3109/01674829809025696

[157] Mous M Muller SA de Leeuw JW. Long-term effects of anal sphincter rupture during vaginal delivery: faecal incontinence and sexual complaints. BJOG 2008; 115: 234–238. 17999696 10.1111/j.1471-0528.2007.01502.x

[158] Oğurlu N Küçük M Aksu H. Influence of employment status on menopausal symptoms. Int J Gynaecol Obstet 2011; 112: 204–207. 21247563 10.1016/j.ijgo.2010.10.010

[159] Ojha J Khatoon F Vashistha P , et al. To study association of post-menopausal symptoms and clinico-demographic profile: a descriptive study. Eras J Med Res 2022; 9: 31–38.

[160] Olafsdottir LB Gudjonsson H Jonsdottir HH , et al. Natural history of irritable bowel syndrome in women and dysmenorrhea: a 10-year follow-up study. Gastroenterol Res Pract 2012; 2012: 534204. 22474441 10.1155/2012/534204 PMC3312222

[161] Özcan H Beji NK. Relationship between lower urinary system complaints and healthy life behaviors among women aged 50 and over. Cukurova Med J 2019; 44: 1392–1399.

[162] Parandavar N Mosalanejad L Ramezanli S , et al. Menopause and crisis? Fake or real: comprehensive search to the depth of crisis experienced: a mixed-method study. Glob J Health Sci 2014; 6: 246–255. 24576387 10.5539/gjhs.v6n2p246 PMC4825262

[163] Parsons J Eccles A Bick D , et al. Women’s experiences of anal incontinence following vaginal birth: a qualitative study of missed opportunities in routine care contacts. PLoS One 2023; 18: e0287779. 10.1371/journal.pone.0287779 PMC10298771 37368897

[164] Resmi S Sukumaran AB Pv B. Climacteric symptoms among women residing in a rural area of Kerala state – a cross-sectional study. Clin Epidemiol Glob Health 2020; 8: 1341–1344.

[165] Rizk DEE Hassan MY Shaheen H , et al. The prevalence and determinants of health care-seeking behavior for fecal incontinence in multiparous United Arab Emirates females. Dis Colon Rectum 2001; 44: 1850–1856. 11742174 10.1007/BF02234467

[166] Safaee A Moghimi-Dehkordi B Pourhoseingholi MA , et al. Bloating in irritable bowel syndrome. Gastroenterol Hepatol Bed Bench 2011; 4: 86–90. 24834162 PMC4017413

[167] Salmoirago-Blotcher E Crawford S Jackson E , et al. Constipation and risk of cardiovascular disease among postmenopausal women. Am J Med 2011; 124: 714–723. 21663887 10.1016/j.amjmed.2011.03.026 PMC3144272

[168] Samtani R Garg D Sharma N , et al. Sociodemographic pattern of postmenopausal women and health issues: a study in rural Bathinda, Punjab. J Midlife Health 2020; 11: 168–170. 33384541 10.4103/jmh.JMH_139_19 PMC7718931

[169] Senthilvel S Vasudevan S Anju PS , et al. Assessment of symptoms and quality of life among postmenopausal women in a tertiary care hospital in Kochi, South India: a hospital-based descriptive study. J Midlife Health 2018; 9: 185–190. 30692813 10.4103/jmh.JMH_98_18 PMC6332724

[170] Shamloufard P Kern M Hooshmand S. Bowel function of postmenopausal women: effects of daily consumption of dried plum. Int J Food Prop 2017; 20: 3006–3013.

[171] Sharma S Tandon VR Mahajan A. Menopausal symptoms in urban women. JK Sci 2007; 9: 13–17.

[172] Sharma VK Saxena MSL . Climacteric symptoms: a study in the Indian context. Maturitas 1981; 3: 11–20. 7253931 10.1016/0378-5122(81)90014-1

[173] Sheereen F Kadarkar KS. Community-based appraisal of menopause-specific health problems and quality of life among women of rural Western Maharashtra. J Fam Med Prim Care 2022; 11: 7328–7334. 10.4103/jfmpc.jfmpc_1377_22 PMC10041302 36993095

[174] Slopien R Pluchino N Warenik-Szymankiewicz A , et al. Correlation between allopregnanolone levels and depressive symptoms during late menopausal transition and early postmenopause. Gynecol Endocrinol 2018; 34: 144–147. 28857628 10.1080/09513590.2017.1371129

[175] Sood R Kuhle C Kapoor E , et al. A negative view of menopause: does the type of symptom matter? Climacteric 2016; 19: 581–587. 27763798 10.1080/13697137.2016.1241227

[176] Stewart DE. Menopause in highland Guatemala Mayan women. Maturitas 2003; 44: 293–297. 12697370 10.1016/s0378-5122(03)00036-7

[177] Terauchi M Hiramitsu S Akiyoshi M , et al. Associations among depression, anxiety and somatic symptoms in peri- and postmenopausal women. J Obstet Gynaecol Res 2013; 39: 1007–1013. 23379427 10.1111/j.1447-0756.2012.02064.x

[178] Terzic S Bapayeva G Kadroldinova N , et al. Menopausal status impact on the quality of life in Kazakhstani healthcare workers: a cross-sectional study. J Gen Intern Med 2024; 39: 969–977. 38315409 10.1007/s11606-024-08650-9 PMC11074092

[179] Tokunaga H Munakata K Katayama K , et al. Clinical data mining related to the Japanese kampo concept “hie” (oversensitivity to coldness) in men and pre- and postmenopausal women. Evid Based Complement Alternat Med 2014; 2014: 832824. 24707313 10.1155/2014/832824 PMC3953564

[180] Wisniewska-Jarosinska M Chojnacki J Konturek S , et al. Evaluation of urinary 6-hydroxymelatonin sulphate excretion in women at different age with irritable bowel syndrome. J Physiol Pharmacol 2010; 61: 295–300. 20610859

[181] Yerra AK Bala S Yalamanchili RK , et al. Menopause-related quality of life among urban women of Hyderabad, India. J Midlife Health 2021; 12: 161–167. 34526752 10.4103/jmh.jmh_272_20 PMC8409714

[182] Yim G Ahn Y Chang Y , et al. Prevalence and severity of menopause symptoms and associated factors across menopause status in Korean women. Menopause 2015; 22: 1108–1116. 25783469 10.1097/GME.0000000000000438

[183] Zhu M Wang S Zhu Y , et al. Behavioral and dietary risk factors of recurrent urinary tract infection in Chinese postmenopausal women: a case-control study. J Int Med Res 2020; 48: 1–15. 10.1177/0300060519889448 PMC7783248 31840544

[184] Soules MR Sherman S Parrott E , et al. Executive summary: stages of reproductive aging workshop (STRAW). Fertil Steril 2001; 76: 874–878. 11704104 10.1016/s0015-0282(01)02909-0

[185] World Health Organization. Research on the menopause in the 1990s. Report of a WHO Scientific Group. World Health Organ Tech Rep Ser 1996; 866: 1–107. 8942292

[186] Lewis JE Hilditch JR Wong CJ. Further psychometric property development of the Menopause-Specific Quality of Life questionnaire and development of a modified version, MENQOL-Intervention questionnaire. Maturitas 2005; 50: 209–221. 15734602 10.1016/j.maturitas.2004.06.015

[187] Jorge JM Wexner SD. Etiology and management of fecal incontinence. Dis Colon Rectum 1993; 36: 77–97. 8416784 10.1007/BF02050307

[188] Im EO. The Midlife Women’s Symptom Index (MSI). Health Care Women Int 2006; 27: 268–287. 16524856 10.1080/07399330500506600

[189] Hunter M. The Women’s Health Questionnaire (WHQ): the development, standardization and application of a measure of mid-aged women’s emotional and physical health. Qual Life Res 2000; 9: 733–738.

[190] Lund KS Siersma VD Christensen KB , et al. Measuring bothersome menopausal symptoms: development and validation of the MenoScores questionnaire. Health Qual Life Outcomes 2018; 16: 97. 29769073 10.1186/s12955-018-0927-6 PMC5956969

[191] Neugarten BL Kraines RJ. “Menopausal symptoms” in women of various ages. Psychosom Med 1965; 27: 266–273. 14327878 10.1097/00006842-196505000-00009

[192] Kaufert PA Gilbert P Hassard T. Researching the symptoms of menopause: an exercise in methodology. Maturitas 1988; 10: 117–131. 3419325 10.1016/0378-5122(88)90156-9

[193] Craig BM Mitchell SA. Examining the value of menopausal symptom relief among US women. Value Health 2016; 19: 158–166. 27021749 10.1016/j.jval.2015.11.002

[194] Khalil J Hill H Kaelber D , et al. The association between hormone replacement therapy and gastroparesis in post-menopausal women: a worldwide database analysis. J Pers Med 2024; 14(3): 275. 38541017 10.3390/jpm14030275 PMC10970917

[195] Avis NE Brockwell S Colvin A. A universal menopausal syndrome? Am J Med 2005; 118: 37–46. 16414325 10.1016/j.amjmed.2005.09.057

[196] Im EO. Ethnic differences in symptoms experienced during the menopausal transition. Health Care Women Int 2009; 30: 339–355. 19255887 10.1080/07399330802695002 PMC2670463

[197] James Lind Alliance Priority Setting Partnerships. Menopause Priority Setting Partnership. Top 10 priorities. The most important questions, https://www.jla.nihr.ac.uk/priority-setting-partnerships/menopause#tab-67281 (2024, accessed 29 November 2024).

[198] Kabir MR Chan K. Menopausal experiences of women of Chinese ethnicity: a meta-ethnography. PLoS One 2023; 18: e0289322. 10.1371/journal.pone.0289322 PMC10499211 37703245

[199] Hoga L Rodolpho J Gonçalves B , et al. Women’s experience of menopause: a systematic review of qualitative evidence. JBI Database System Rev Implement Rep 2015; 13(8): 250–337. 10.11124/jbisrir-2015-1948 26455946

[200] Lawson AK Marsh EE. Hearing the silenced voices of underserved women: the role of qualitative research in gynecologic and reproductive care. Obstet Gynecol Clin North Am 2017; 44: 109–120. 28160888 10.1016/j.ogc.2016.11.005 PMC5310813

[201] Harlow SD Sievert LL LaCroix AZ , et al. Women’s midlife health: the unfinished research agenda. Womens Midlife Health 2023; 9: 7. 37784201 10.1186/s40695-023-00090-5 PMC10546728

[202] Carpenter JS Sheng Y Elomba CD , et al. A systematic review of palpitations prevalence by menopausal status. Curr Obstet Gynecol Rep 2021; 10: 7–13.

[203] Min SH Yang Q Min SW , et al. Are there differences in symptoms experienced by midlife climacteric women with and without metabolic syndrome? A scoping review. Womens Health 2022; 18: 1–17. 10.1177/17455057221083817 PMC8918770 35266423

[204] Fang Y Liu F Zhang X , et al. Mapping global prevalence of menopausal symptoms among middle-aged women: a systematic review and meta-analysis. BMC Public Health 2024; 24: 1767. 38956480 10.1186/s12889-024-19280-5 PMC11220992

[205] Khani S Azizi M Elyasi F , et al. The prevalence of sexual dysfunction in the different menopausal stages: a systematic review and meta-analysis. Int J Sex Health 2021; 33: 439–472. 38595744 10.1080/19317611.2021.1926039 PMC10903585

[206] Gyllstrom ME Schreiner PJ Harlow BL. Perimenopause and depression: strength of association, causal mechanisms and treatment recommendations. Best Pract Res Clin Obstet Gynaecol 2007; 21: 275–292. 17166771 10.1016/j.bpobgyn.2006.11.002

[207] Kesmodel US. Cross-sectional studies - what are they good for? Acta Obstet Gynecol Scand 2018; 97: 388–393. 29453895 10.1111/aogs.13331

[208] Collins A Landgren B-M. Longitudinal research on the menopause-methodological challenges. Acta Obstet Gynecol Scand 2002; 81: 579–580. 12190830 10.1034/j.1600-0412.2002.810701.x

[209] Pavlović JM Derby CA. Pain in midlife women: a growing problem in need of further research. Womens Midlife Health 2022; 8: 4. 35509086 10.1186/s40695-022-00074-x PMC9068256

[210] Maki PM Weber MT. A research primer for studies of cognitive changes across the menopause transition. Climacteric 2021; 24: 382–388. 34240671 10.1080/13697137.2021.1905625

[211] Anaya C Culbert KM Klump KL. Binge eating risk during midlife and the menopausal transition: sensitivity to ovarian hormones as potential mechanisms of risk. Curr Psychiatry Rep 2023; 25: 45–52. 36565385 10.1007/s11920-022-01405-5 PMC9974637

[212] Avis NE Crawford SL Greendale G , et al. Duration of menopausal vasomotor symptoms over the menopause transition. JAMA Intern Med 2015; 175: 531–539. 25686030 10.1001/jamainternmed.2014.8063 PMC4433164

[213] Melby MK Lock M Kaufert P. Culture and symptom reporting at menopause. Hum Reprod Update 2005; 11: 495–512. 15919681 10.1093/humupd/dmi018

[214] Phipps AI Ichikawa L Bowles EJ , et al. Defining menopausal status in epidemiologic studies: a comparison of multiple approaches and their effects on breast cancer rates. Maturitas 2010; 67: 60–66. 20494530 10.1016/j.maturitas.2010.04.015 PMC2922404

[215] Palacios S Henderson VW Siseles N , et al. Age of menopause and impact of climacteric symptoms by geographical region. Climacteric 2010; 13: 419–428. 20690868 10.3109/13697137.2010.507886

[216] Sydora BC Fast H Campbell S , et al. Use of the Menopause-Specific Quality of Life (MENQOL) questionnaire in research and clinical practice: a comprehensive scoping review. Menopause 2016; 23: 1038–1051. 27300115 10.1097/GME.0000000000000636

[217] Gartoulla P Islam MR Bell RJ , et al. Prevalence of menopausal symptoms in Australian women at midlife: a systematic review. Climacteric 2014; 17: 529–539. 24245562 10.3109/13697137.2013.865721

[218] Iliodromiti S Wang W Lumsden MA , et al. Variation in menopausal vasomotor symptoms outcomes in clinical trials: a systematic review. BJOG 2020; 127: 320–333. 31621155 10.1111/1471-0528.15990 PMC6972542

[219] Graham EE Michala L Hachfeld A , et al. Collection of menopause data in studies of women living with HIV: a systematic literature review. HIV Med 2024; 25: 174–187. 37776176 10.1111/hiv.13552

[220] Islam MR Gartoulla P Bell RJ , et al. Prevalence of menopausal symptoms in Asian midlife women: a systematic review. Climacteric 2015; 18: 157–176. 24978151 10.3109/13697137.2014.937689

[221] Kingsberg SA Schulze-Rath R Mulligan C , et al. Global view of vasomotor symptoms and sleep disturbance in menopause: a systematic review. Climacteric 2023; 26: 537–549. 37751852 10.1080/13697137.2023.2256658

[222] Melby MK Sievert LL Anderson D , et al. Overview of methods used in cross-cultural comparisons of menopausal symptoms and their determinants: Guidelines for Strengthening the Reporting of Menopause and Aging (STROMA) studies. Maturitas 2011; 70: 99–109. 21840143 10.1016/j.maturitas.2011.07.011

[223] Stull DE Leidy NK Parasuraman B , et al. Optimal recall periods for patient-reported outcomes: challenges and potential solutions. Curr Med Res Opin 2009; 25: 929–942. 19257798 10.1185/03007990902774765

[224] von Elm E Altman DG Egger M , et al. The Strengthening the Reporting of Observational Studies in Epidemiology (STROBE) statement: guidelines for reporting observational studies. Ann Intern Med 2007; 147: 573–577. 17938396 10.7326/0003-4819-147-8-200710160-00010

[225] Carpenter JS Sheng Y Pike C , et al. Correlates of palpitations during menopause: a scoping review. Womens Health 2022; 18: 1–14. 10.1177/17455057221112267 PMC9289918 35833667

[226] Suares NC Ford AC. Prevalence of, and risk factors for, chronic idiopathic constipation in the community: systematic review and meta-analysis. Am J Gastroenterol 2011; 106: 1582–1591. 21606976 10.1038/ajg.2011.164

[227] Hirt J Nordhausen T Appenzeller-Herzog C , et al. Citation tracking for systematic literature searching: a scoping review. Res Synth Methods 2023; 14: 563–579. 37042216 10.1002/jrsm.1635

[228] Grant MJ Booth A. A typology of reviews: an analysis of 14 review types and associated methodologies. Health Info Libr J 2009; 26: 91–108. 19490148 10.1111/j.1471-1842.2009.00848.x

[229] Ruigómez A García Rodríguez LA Johansson S , et al. Is hormone replacement therapy associated with an increased risk of irritable bowel syndrome? Maturitas 2003; 44: 133–140. 12590009 10.1016/s0378-5122(02)00321-3

[230] Staller K Townsend MK Khalili H , et al. Menopausal hormone therapy is associated with increased risk of fecal incontinence in women after menopause. Gastroenterology 2017; 152: 1915–1921.e1911. 10.1053/j.gastro.2017.02.005 PMC5447480 28209529

[231] Khatibi EA Samsioe G Li C , et al. Does hormone therapy increase allergic reactions and upper gastrointestinal problems? Results from a population-based study of Swedish woman. The Women’s Health in the Lund Area (WHILA) study. Maturitas 2004; 48: 438–445. 15283937 10.1016/j.maturitas.2003.10.001

[232] Al Wattar BH Rogozinska E Vale C , et al. Effectiveness and safety of menopause treatments: pitfalls of available evidence and future research need. Climacteric 2024; 27: 154–158. 38275167 10.1080/13697137.2023.2297880

[233] Mendelson S Anbukkarasu P Cassisi JE , et al. Gastrointestinal functioning and menstrual cycle phase in emerging young adult women: a cross-sectional study. BMC Gastroenterol 2023; 23: 406. 37990300 10.1186/s12876-023-03036-3 PMC10664285

[234] BMJ Best Practice. Assessment of chronic abdominal pain, https://bestpractice.bmj.com/topics/en-gb/767/diagnosis-approach (2023, accessed 4 July 2025).

