# Supplementary material for: The volume and characteristics of research on gastrointestinal symptoms in ‘natural’ peri- and postmenopause: A scoping review
Source: Womens Health (Lond). 2025 Oct 27;21:17455057251387470. doi: 10.1177/17455057251387470 (PMC12575958; doi:10.1177/17455057251387470)
Supplement: sj-docx-1-whe-10.1177_17455057251387470 – Supplemental material for The volume and characteristics of research on gastrointestinal symptoms in ‘natural’ peri- and postmenopause: A scoping review [file sj-docx-1-whe-10.1177_17455057251387470.docx]

Supplemental Appendix 1: PRISMA checklist for a scoping review

| **SECTION** | **ITEM** | **PRISMA-ScR CHECKLIST ITEM** | **REPORTED on PAGE #** |
| --- | --- | --- | --- |
| **TITLE** | | | |
| **Title** | 1 | Identify the report as a scoping review | 1 |
| **ABSTRACT** | | | |
| **Structured summary** | 2 | Provide a structured summary that includes (as applicable): background, objectives, eligibility criteria, sources of evidence, charting methods, results and conclusions that relate to the review questions and objectives | 1-2 |
| **INTRODUCTION** | | | |
| **Rationale** | 3 | Describe the rationale for the review in the context of what is already known. Explain why the review questions/objectives lend themselves to a scoping review approach | 2-5 Justification for use of a scoping review approach is provided in Methods (7) |
| **Objectives** | 4 | Provide an explicit statement of the questions and objectives being addressed with reference to their key elements (e.g., population or participants, concepts, and context) or other relevant key elements used to conceptualize the review questions and/or objectives. | 5 |
| **METHODS** | | | |
| **Protocol and registration** | 5 | Indicate whether a review protocol exists, state if and where it can be accessed (e.g. a Web address); and if available, provide registration information, including the registration number | 6 |
| **Eligibility criteria** | 6 | Specific characteristics of the sources of evidence used as eligibility criteria (e.g., years considered, language and publication status) and provide a rationale | 6-9 |
| **Information Sources** | 7 | Describe all information sources in the search (e.g. databases with dates of coverage and contact with authors to identify additional sources) as well as the date the most recent search was executed | 10-12 |
| **Search** | 8 | Present the full electronic search strategy for at least 1 database, including any limits used, such that it could be repeated. | 11. Full strategies in Supplemental Appendix 3. |
| **Selection of sources of evidence** | 9 | State the process for selecting sources of evidence (i.e., screening and eligibility) included in the scoping review | 12 |
| **Data charting process** | 10 | Describe the methods of charting data from the included sources of evidence (e.g. calibrated forms or forms that have been tested by the team before their use, and whether data charting was done independently or in duplicate) and any processes for obtaining and confirming data from investigators | 12-13 |
| **Data items** | 11 | List and define all variables for which data were sought and any assumptions and simplifications made | 13-14 |
| **Critical appraisal of individual sources of evidence** | 12 | If done, provide a rationale for conducting a critical appraisal of included sources of evidence; describe the methods used and how this information was used in any data synthesis (if appropriate) | N/A |
| **Synthesis of results** | 13 | Describe the methods of handling and summarizing the data that were charted | 13-14 |
| **RESULTS** | | | |
| **Selection of sources of evidence** | 14 | Given numbers of sources of evidence screened, assessed for eligibility, and included in the review, with reasons for exclusion at each stage, ideally using a flow diagram | 14, PRISMA flow diagram (Figure 2) |
| **Characteristics of sources of evidence** | 15 | For each source of evidence, present characteristics for which data were charted and provide the citations | 15-16 |
| **Critical appraisal within sources of evidence** | 16 | If done, present data on critical appraisal of included sources of evidence (see item 12) | N/A |
| **Results of individual sources of evidence** | 17 | For each included source of evidence present the relevant data that were charted that relate to the review questions and objectives | [Appendix](#_Appendix_9._Table) 5 |
| **Synthesis of results** | 18 | Summarize and/or present the charting results as they relate to the review questions and objectives | 15-20 |
| **DISCUSSION** | | | |
| **Summary of evidence** | 19 | Summarize the main results (including an overview of concepts, themes and types of evidence available), link to the review questions and objectives, and consider the relevance to key groups | 20-24 |
| **Limitations** | 20 | Discussion the limitations of the scoping review process | 24 |
| **Conclusions** | 21 | Provide a general interpretation of the results with respect to the review questions and objectives, as well as potential implications and/or next steps | 25-26 |
| **FUNDING** | | | |
| **Funding** | 22 | Describe sources of funding for the included sources of evidence, as well as sources of funding for the scoping review. Describe the role of funders of the scoping review | Data was gathered on the sources of funding for included studies, however, there was insufficient space for inclusion in the final review. This information is available on request.  The authors did not receive funding for this scoping review. |
