# Supplementary material for: The volume and characteristics of research on gastrointestinal symptoms in ‘natural’ peri- and postmenopause: A scoping review
Source: Womens Health (Lond). 2025 Oct 27;21:17455057251387470. doi: 10.1177/17455057251387470 (PMC12575958; doi:10.1177/17455057251387470)
Supplement: sj-docx-2-whe-10.1177_17455057251387470 – Supplemental material for The volume and characteristics of research on gastrointestinal symptoms in ‘natural’ peri- and postmenopause: A scoping review [file sj-docx-2-whe-10.1177_17455057251387470.docx]

Supplemental Appendix 2: Protocol amendments with justifications

| **Section** | **Protocol amendment** | **Justification/rationale** |
| --- | --- | --- |
| Eligibility criteria | The eligibility criteria were amended to **exclude** studies focusing on GI symptoms as an adverse event of hormone replacement therapy (HRT), or HRT as an intervention to treat GI symptoms in perimenopausal or postmenopausal individuals. | While HRT appears to be a potential factor influencing women’s experiences of GI symptoms in the menopause, pilot screening identified large numbers of included studies, with impacts on the feasibility of this scoping review. In consequence, studies focusing on HRT were excluded. |
| Data charting template | **Timeframe of recall** over which GI symptom was measured (e.g. prior 12 months) | This was added as a data item for charting related to the conduct of research in this area. |
| Data analysis and presentation | Journal names, funding sources, and population characteristics reported (including age, ethnicity, education, socioeconomic status), sample sizes, and analyses broken down by GI symptom were not included in presentation of results. | It was not feasible to present and discuss details of all 82 journals publishing studies, funding sources, sample sizes or population characteristics beyond details of menopausal stages studied. |
