# Supplementary material for: The volume and characteristics of research on gastrointestinal symptoms in ‘natural’ peri- and postmenopause: A scoping review
Source: Womens Health (Lond). 2025 Oct 27;21:17455057251387470. doi: 10.1177/17455057251387470 (PMC12575958; doi:10.1177/17455057251387470)
Supplement: sj-docx-3-whe-10.1177_17455057251387470 – Supplemental material for The volume and characteristics of research on gastrointestinal symptoms in ‘natural’ peri- and postmenopause: A scoping review [file sj-docx-3-whe-10.1177_17455057251387470.docx]

Appendix 3: Search strategies

Search strategies for bibliographic databases

| **Bibliographic database/source** | **Platform/**  **Publisher** | **Dates of coverage** |
| --- | --- | --- |
| MEDLINE ® and Epub Ahead of Print, In-Process, In-Data-Review & Other Non-Indexed Citations, Daily and Versions | Ovid | 1946-present |
| Embase | Ovid | 1974- present |
| APA PsycINFO | Ovid | 1806- present |
| Cochrane Database of Systematic Reviews | Wiley | 1996- present |
| Cochrane Central Register of Controlled Trials (CENTRAL) | Wiley | 1908- present |
| CINAHL Plus with Full Text | EBSCO | 1937- present |
| AMED (Allied and Complementary Medicine Database) | EBSCO | 1995- present |
| Scopus | Elsevier | 1788- present |
| Web of Science Core Collection including:   - Science Citation Index Expanded - Social Sciences Citation Index - Arts & Humanities Citation Index - Conference Proceedings Citation Index – Science - Conference Proceedings Citation index – Social Science & Humanities - Emerging Sources Citation Index | Clarivate Analytics | 1970- present  1970- present  1975- present  1990- present  1990- present  2015- present |
| ProQuest Dissertations & Theses Global | ProQuest | 1743- present |
| ClinicalTrials.gov (<https://clinicaltrials.gov>) | National Library of Medicine | 2000- present |
| WHO International Clinical Trials Registry Platform (ICTRP) (<https://www.who.int/clinical-trials-registry-platform>) | World Health Organization | 2006- present |

MEDLINE

MEDLINE ® and Epub Ahead of Print, In-Process, In-Data-Review & Other Non-Indexed Citations, Daily and Versions <1946 to March 01, 2024>

Search completed: 4^th^ March 2024

1 exp Menopause/ 63887

2 (menopaus* or perimenopaus* or peri-menopaus* or postmenopaus* or post-menopaus* or postreproductive or post-reproductive or climacteric).ti,ab. 108088

3 1 or 2 122525

4 Irritable Bowel Syndrome/ 9731

5 exp Inflammatory Bowel Diseases/ 99756

6 exp "signs and symptoms, digestive"/ 171165

7 ((digestive or bowel or gut or gastro* or colonic) adj2 (symptom* or habit* or issue* or issue* or problem* or dysfunction* or complaint*)).ti,ab. 40016

8 ((digestive or gastro*) adj3 symptom*).ti,ab. 26697

9 (GI adj symptom*).ti,ab. 3197

10 IBS.ti,ab. 11431

11 (inflammatory adj bowel).ti,ab. 63103

12 (ulcerative adj colitis).ti,ab. 47845

13 crohn*.ti,ab. 55552

14 (irritable adj bowel).ti,ab. 16281

15 (diarrh* or constipat*).ti,ab. 152296

16 ((loose or watery) adj stool*).ti,ab. 1882

17 ((bowel* or defecat*) adj2 (frequen* or urgen* or infrequen*)).ti,ab. 2728

18 (incomplete adj evacuation).ti,ab. 466

19 (bloating or bloated or gassiness or gaseousness or flatulence or flatulent or flatus or (abdom* adj disten*) or (swollen adj abdom*) or (swelling adj2 abdom*) or (postprandial adj fullness) or (post-prandial adj fullness)).ti,ab. 18759

20 ((gurgling or rumbling) adj2 (abdom* or stomach or gastro*)).ti,ab. 32

21 ((abdom* or stomach or epigastric or rectal or rectum or belly) adj2 (pain* or cramp* or ache* or colic or discomfort)).ti,ab. 85637

22 (reflux or GERD or dyspepsia or indigestion or heartburn or regurgitat*).ti,ab. 119679

23 (belch* or burp* or eructation or hiccup*).ti,ab. 3608

24 (nausea* or vomit* or emesis or retching).ti,ab. 113848

25 ((faecal or fecal or anal or bowel) adj2 (incontinen* or leak* or soiling)).ti,ab. 10141

26 ((bowel* adj control*) or encopresis).ti,ab. 1053

27 ((gut or digestive or gastro* or bowel* or colon*) adj2 health*).ti. 1418

28 or/4-27 661814

29 3 and 28 2173

30 exp animals/ not humans/ 5197326

31 29 not 30 2151

Embase

Embase <1974 to 2024 March 01>

Search completed: 4^th^ March 2024

1 *"menopause and climacterium"/ or *climacterium/ or *menopause/ or *postmenopause/ 50605

2 (menopaus* or perimenopaus* or peri-menopaus* or postmenopaus* or post-menopaus* or postreproductive or post-reproductive or climacteric).ti,ab. 160058

3 1 or 2 164417

4 *irritable colon/ 17065

5 *inflammatory bowel disease/ or *crohn disease/ or *ulcerative colitis/ 121365

6 *gastrointestinal symptom/ 12549

7 *constipation/ or *chronic constipation/ 16206

8 *diarrhea/ or *acute diarrhea/ or *chronic diarrhea/ 40170

9 *bloating/ or *abdominal distension/ 1037

10 *abdominal pain/ or *lower abdominal pain/ or *upper abdominal pain/ 13871

11 *gastroesophageal reflux/ 26281

12 *heartburn/ 1884

13 *dyspepsia/ 9676

14 *eructation/ 229

15 *hiccup/ 1374

16 *vomiting/ 19008

17 *nausea/ 15785

18 *retching/ 55

19 *feces incontinence/ 8804

20 ((digestive or bowel or gut or gastro* or colonic) adj2 (symptom* or habit* or issue* or issue* or problem* or dysfunction* or complaint*)).ti,ab. 66314

21 ((digestive or gastro*) adj3 symptom*).ti,ab. 43866

22 (GI adj symptom*).ti,ab. 8390

23 IBS.ti,ab. 21012

24 (inflammatory adj bowel).ti,ab. 109321

25 (ulcerative adj colitis).ti,ab. 79938

26 crohn*.ti,ab. 100259

27 (irritable adj bowel).ti,ab. 26919

28 (diarrh* or constipat*).ti,ab. 238896

29 ((loose or watery) adj stool).ti,ab. 1016

30 ((bowel* or defecat*) adj2 (frequen* or urgen* or infrequen*)).ti,ab. 4627

31 (incomplete adj evacuation).ti,ab. 924

32 (bloating or bloated or gassiness or gaseousness or flatulence or flatulent or flatus or (abdom* adj disten*) or (swollen adj abdom*) or (swelling adj2 abdom*) or (postprandial adj fullness) or (post-prandial adj fullness)).ti,ab. 34114

33 ((gurgling or rumbling) adj2 (abdom* or stomach or gastro*)).ti,ab. 61

34 ((abdom* or stomach or epigastric or rectal or rectum or belly) adj2 (pain* or cramp* or ache* or colic or discomfort)).ti,ab. 156531

35 (reflux adj2 (gastro* or oesophag* or esophag*)).ti,ab. 50142

36 (GERD or dyspepsia or indigestion or heartburn or regurgitat*).ti,ab. 124266

37 (belch* or burp* or eructation or hiccup*).ti,ab. 5561

38 (nausea* or vomit* or emesis or retching).ti,ab. 206736

39 ((faecal or fecal or anal or bowel) adj2 (incontinen* or leak* or soiling)).ti,ab. 17307

40 ((bowel* adj control*) or encopresis).ti,ab. 1547

41 ((gut or digestive or gastro* or bowel* or colon*) adj2 health*).ti. 1687

42 or/4-41 946222

43 3 and 42 3960

44 (rat or rats or mouse or mice or swine or porcine or murine or sheep or lambs or pigs or piglets or rabbit or rabbits or cat or cats or dog or dogs or cattle or bovine or monkey or monkeys or trout or marmoset$1).ti. and animal experiment/ 1242115

45 animal experiment/ not (human experiment/ or human/) 2610438

46 44 or 45 2682312

47 43 not 46 3927

APA PsycINFO

APA PsycInfo <1806 to February Week 5 2024>

Search completed: 4^th^ March 2024

1 menopause/ 4228

2 (menopaus* or perimenopaus* or peri-menopaus* or postmenopaus* or post-menopaus* or postreproductive or post-reproductive or climacteric).ti,ab. 7408

3 1 or 2 7624

4 ulcerative colitis/ 339

5 exp colon disorders/ 5239

6 vomiting/ 1260

7 Nausea/ 1051

8 Dyspepsia/ 223

9 ((digestive or bowel or gut or gastro* or colonic) adj2 (symptom* or habit* or issue* or issue* or problem* or dysfunction* or complaint*)).ti,ab. 2446

10 ((digestive or gastro*) adj3 symptom*).ti,ab. 1355

11 (GI adj symptom*).ti,ab. 305

12 IBS.ti,ab. 1302

13 (inflammatory adj bowel).ti,ab. 1190

14 (ulcerative adj colitis).ti,ab. 650

15 crohn*.ti,ab. 703

16 (irritable adj bowel).ti,ab. 1766

17 (diarrh* or constipat*).ti,ab. 4267

18 ((loose or watery) adj stool).ti,ab. 7

19 ((bowel* or defecat*) adj2 (frequen* or urgen* or infrequen*)).ti,ab. 122

20 (incomplete adj evacuation).ti,ab. 15

21 (bloating or bloated or gassiness or gaseousness or flatulence or flatulent or flatus or (abdom* adj disten*) or (swollen adj abdom*) or (swelling adj2 abdom*) or (postprandial adj fullness) or (post-prandial adj fullness)).ti,ab. 407

22 ((gurgling or rumbling) adj2 (abdom* or stomach or gastro*)).ti,ab. 2

23 ((abdom* or stomach or epigastric or rectal or rectum or belly) adj2 (pain* or cramp* or ache* or colic or discomfort)).ti,ab. 2376

24 (reflux or GERD or dyspepsia or indigestion or heartburn or regurgitat*).ti,ab. 1777

25 (belch* or burp* or eructation or hiccup*).ti,ab. 329

26 (nausea* or vomit* or emesis or retching).ti,ab. 9298

27 ((faecal or fecal or anal or bowel) adj2 (incontinen* or leak* or soiling)).ti,ab. 416

28 ((bowel* adj control*) or encopresis).ti,ab. 650

29 ((gut or digestive or gastro* or bowel* or colon*) adj2 health*).ti. 28

30 or/4-29 23891

31 3 and 30 86

32 animals/ not humans/ 7491

33 31 not 32 86

Cochrane Database of Systematic Reviews and Cochrane Central Register of Controlled Trials (CENTRAL)

Search completed: 4^th^ March 2024

#1 MeSH descriptor: [Menopause] explode all trees 9139

#2 (menopaus* or peri-menopaus* or perimenopaus* or postmenopaus* or post-menopaus* or postreproductive or post-reproductive or climacteric):ti,ab,kw 31830

#3 #1 or #2 32487

#4 MeSH descriptor: [Irritable Bowel Syndrome] explode all trees 1797

#5 MeSH descriptor: [Signs and Symptoms, Digestive] explode all trees 19349

#6 ((digestive or bowel or gut or gastro* or colonic) NEAR/2 (symptom* or habit* or issue* or issue* or problem* or dysfunction* or complaint*)):ti,ab,kw 13894

#7 ((digestive or gastro*) NEAR/3 symptom*):ti,ab,kw 11049

#8 (GI NEAR/1 symptom*) 1162

#9 IBS:ti,ab,kw 3768

#10 (inflammatory near/1 bowel):ti,ab,kw 4382

#11 (irritable near/1 bowel):ti,ab,kw 4834

#12 (diarrh* or constipat*):ti,ab,kw 44566

#13 ((loose or water) near/1 stool*):ti,ab,kw 576

#14 ((bowel* or defecat*) near/2 (frequen* or urgen* or infrequen*)):ti,ab,kw 1591

#15 (incomplete adj evacuation):ti,ab,kw 0

#16 (bloating or bloated or gassiness or wind or flatulence or flatulent or flatus or (abdom* near/1 disten*) or (swollen near/1 abdom*) or (swelling near/1 abdom*) or (postprandial near/1 fullness) or (post-prandial near/1 fullness)):ti,ab,kw 9794

#17 ((gurgling or rumbling) near/2 (abdom* or stomach or gastro*)):ti,ab,kw 32

#18 ((abdom* or stomach or epigastric or rectal or rectum or belly) near/2 (pain* or cramp* or ache* or colic discomfort)):ti,ab,kw 20278

#19 (reflux or GERD or dyspepsia or indigestion or heartburn or regurgitat*):ti,ab,kw 18264

#20 (belch* or burp* or eructation or hiccup*):ti,ab,kw 1252

#21 (nausea* or vomit* or emesis or retching):ti,ab,kw 68314

#22 ((faecal or fecal or anal or bowel) near/2 (incontinen* or leak* or soiling)):ti,ab,kw 1980

#23 ((bowel* near/1 control) or encopresis):ti,ab,kw 143

#24 ((gut or digestive or gastro* or bowel* or colon*) near/2 health*):ti 326

#25 #4 or #5 or #6 or #7 or #8 or #9 or #10 or #11 or #12 or #13 or #14 or #15 or #16 or #17 or #18 or #19 or #20 or #21 or #22 or #23 or #24 133886

#26 #3 and #25 1572 (18 Cochrane reviews; 1554 in CENTRAL)

CINAHL Plus with Full Text

Search completed: 4^th^ March 2024

S30 S5 AND S29 656

S29 S6 OR S7 OR S8 OR S9 OR S10 OR S11 OR S12 OR S13 OR S14 OR S15 OR S16 OR S17 OR S18 OR S19 OR S20 OR S21 OR S22 OR S23 OR S24 OR S25 OR S26 OR S27 OR S28 143,559

S28 TI ((gut or digestive or gastro* or bowel* or colon*) N2 health*) 884

S27 TI ( ((bowel* N2 control*) or encopresis) ) OR AB ( ((bowel* N2 control*) or encopresis) ) 554

S26 TI ( ((faecal or fecal or anal or bowel) N2 (incontinen* or leak* or soiling)) ) OR AB ( ((faecal or fecal or anal or bowel) N2 (incontinen* or leak* or soiling)) ) 2,887

S25 TI ( (nausea* or vomit* or emesis or retching) ) OR AB ( (nausea* or vomit* or emesis or retching) ) 28,084

S24 TI ( (belch* or burp* or eructation or hiccup*) ) NOT AB ( (belch* or burp* or eructation or hiccup*) ) 317

S23 TI ( (reflux or GERD or dyspepsia or indigestion or heartburn or regurgitat*) ) OR AB ( (reflux or GERD or dyspepsia or indigestion or heartburn or regurgitat*) ) 22,783

S22 TI ( ((abdom* or stomach or epigastric or rectal or rectum or belly) N2 (pain* or cramp* or ache* or colic or discomfort)) ) OR AB ( ((abdom* or stomach or epigastric or rectal or rectum or belly) N2 (pain* or cramp* or ache* or colic or discomfort)) ) 17,826

S21 TI ( ((gurgling or rumbling) N2 (abdom* or stomach or gastro*)) ) OR AB ( ((gurgling or rumbling) N2 (abdom* or stomach or gastro*)) ) 10

S20 TI ( (bloating or bloated or gassiness or gaseousness or flatulence or flatulent or flatus or (abdom* N2 disten*) or (swollen N2 abdom*) or (swelling N2 abdom*) or (postprandial N1 fullness) or (post-prandial N1 fullness)) ) OR AB ( (bloating or bloated or gassiness or gaseousness or flatulence or flatulent or flatus or (abdom* N2 disten*) or (swollen N2 abdom*) or (swelling N2 abdom*) or (postprandial N1 fullness) or (post-prandial N1 fullness)) ) 4,215

S19 TI incomplete evacuation OR AB incomplete evacuation 117

S18 TI ( ((bowel* or defecat*) N2 (frequen* or urgen* or infrequen*)) ) OR AB ( ((bowel* or defecat*) N2 (frequen* or urgen* or infrequen*)) ) 806

S17 TI ( ((loose or watery) N1 stool*) ) OR AB ( ((loose or watery) N1 stool*) ) 404

S16 TI ( diarrh* or constipat* ) OR AB ( diarrh* or constipat* ) 28,934

S15 TI (irritable N1 bowel) OR AB (irritable N1 bowel) 4,377

S14 TI crohn* OR AB crohn* 9,551

S13 TI ulcerative colitis OR AB ulcerative colitis 7,189

S12 TI inflammatory N1 bowel OR AB inflammatory N1 bowel 12,089

S11 TI IBS OR AB IBS 2,765

S10 TI ( ((digestive or gastro*) N3 symptom*) ) OR AB ( ((digestive or gastro*) N3 symptom*) ) OR TI ( ((GI) N1 symptom*) ) OR AB ( ((GI) N1 symptom*) ) 6,800

S9 TI ( ((digestive or bowel or gut or gastro* or colonic) N2 (symptom* or habit* or issue* or issue* or problem* or dysfunction* or complaint*)) ) OR AB ( ((digestive or bowel or gut or gastro* or colonic) N2 (symptom* or habit* or issue* or issue* or problem* or dysfunction* or complaint*)) ) 11,198

S8 (MH "Signs and Symptoms, Digestive+") 42,124

S7 (MH "Inflammatory Bowel Diseases+") 19,846

S6 (MH "Irritable Bowel Syndrome") 4,939

S5 S1 OR S2 OR S3 OR S4 36,998

S4 (MH "Postmenopausal Disorders") 675

S3 (MH "Perimenopausal Symptoms") 2,691

S2 TI ( (menopaus* or perimenopaus* or peri-menopaus* or postmenopaus* or post-menopaus* or postreproductive or post-reproductive or climacteric) ) OR AB ( (menopaus* or perimenopaus* or peri-menopaus* or postmenopaus* or post-menopaus* or postreproductive or post-reproductive or climacteric) ) 33,325

S1 (MH "Menopause") 10,170

AMED

Search completed: 4^th^ March 2024

S38 S5 AND S37 23

S37 S6 OR S7 OR S8 OR S9 OR S10 OR S11 OR S12 OR S13 OR S14 OR S15 OR S16 OR S17 OR S18 OR S19 OR S20 OR S21 OR S22 OR S23 OR S24 OR S25 OR S26 OR S27 OR S28 OR S29 OR S30 OR S31 OR S32 OR S33 OR S34 OR S35 OR S36 4,839

S36 TI ((gut or digestive or gastro* or bowel* or colon*) N2 health*) 23

S35 TI ( ((bowel* N2 control*) or encopresis) ) OR AB ( ((bowel* N2 control*) or encopresis) ) 44

S34 TI ( ((faecal or fecal or anal or bowel) N2 (incontinen* or leak* or soiling)) ) OR AB ( ((faecal or fecal or anal or bowel) N2 (incontinen* or leak* or soiling)) ) 129

S33 TI ( (nausea* or vomit* or emesis or retching) ) OR AB ( (nausea* or vomit* or emesis or retching) ) 1,166

S32 TI ( (belch* or burp* or eructation or hiccup*) ) OR AB ( (belch* or burp* or eructation or hiccup*) ) 91

S31 TI ( (reflux or GERD or dyspepsia or indigestion or heartburn or regurgitat*) ) OR AB ( (reflux or GERD or dyspepsia or indigestion or heartburn or regurgitat*) ) 476

S30 TI ( ((abdom* or stomach or epigastric or rectal or rectum or belly) N2 (pain* or cramp* or ache* or colic or discomfort)) ) OR AB ( ((abdom* or stomach or epigastric or rectal or rectum or belly) N2 (pain* or cramp* or ache* or colic or discomfort)) ) 544

S29 TI ( ((gurgling or rumbling) N2 (abdom* or stomach or gastro*)) ) OR AB ( ((gurgling or rumbling) N2 (abdom* or stomach or gastro*)) ) 0

S28 TI ( (bloating or bloated or gassiness or gaseousness or flatulence or flatulent or flatus or (abdom* N2 disten*) or (swollen N2 abdom*) or (swelling N2 abdom*) or (postprandial fullness) or (post-prandial fullness)) ) OR AB ( (bloating or bloated or gassiness or gaseousness or flatulence or flatulent or flatus or (abdom* N2 disten*) or (swollen N2 abdom*) or (swelling N2 abdom*) or (postprandial fullness) or (post-prandial fullness)) ) 207

S27 TI (incomplete evacuation) OR AB (incomplete evacuation) 6

S26 TI ( ((bowel* or defecat*) N2 (frequen* or urgen* or infrequen*)) ) OR AB ( ((bowel* or defecat*) N2 (frequen* or urgen* or infrequen*)) ) 50

S25 TI ( ((loose or watery) N1 stool*) ) OR AB ( ((loose or watery) N1 stool*) ) 17

S24 TI ( (diarrh* or constipat*) ) OR AB ( (diarrh* or constipat*) ) 1,463

S23 TI (irritable bowel) OR AB (irritable bowel) 355

S22 TI crohn* OR AB crohn* 120

S21 TI (ulcerative colitis) OR AB (ulcerative colitis) 291

S20 TI (inflammatory bowel) OR AB (inflammatory bowel) 195

S19 TI IBS OR AB IBS 185

S18 TI ( ((digestive or gastro*) N3 symptom*) ) OR AB ( ((digestive or gastro*) N3 symptom*) ) 194

S17 TI ( ((digestive or bowel or gut or gastro* or colonic) N2 (symptom* or habit* or issue* or issue* or problem* or dysfunction*)) ) OR AB ( ((digestive or bowel or gut or gastro* or colonic) N2 (symptom* or habit* or issue* or issue* or problem* or dysfunction*)) ) 486

S16 (ZU "fecal incontinence") 122

S15 (ZU "gastroesophageal reflux") 92

S14 (ZU "heartburn") 3

S13 (ZU "abdominal pain") 102

S12 (ZU "vomiting") 334

S11 (ZU "nausea") 352

S10 (ZU "diarrhea") 407

S9 (ZU "constipation") 348

S8 (ZU "digestive symptoms") 38

S7 (ZU "inflammatory bowel disease") 130

S6 (ZU "irritable bowel syndrome") 218

S5 S1 OR S2 OR S3 OR S4 1,295

S4 TI ( (menopaus* or perimenopaus* or peri-menopaus* or postmenopaus* or post-menopaus* or postreproductive or post-reproductive or climacteric) ) OR AB ( (menopaus* or perimenopaus* or peri-menopaus* or postmenopaus* or post-menopaus* or postreproductive or post-reproductive or climacteric) ) 1,175

S3 (ZU "postmenopause") 200

S2 (ZU "perimenopause") 6

S1 (ZU "menopause") 640

Scopus

Search completed: 4^th^ March 2024

( ( TITLE ( ( ( gut OR digestive OR gastro* OR bowel* OR colon* ) W/2 health* ) ) ) OR ( ( TITLE ( ( ( bowel* W/1 control* ) OR encopresis ) ) OR ABS ( ( ( bowel* W/1 control* ) OR encopresis ) ) ) ) OR ( ( TITLE ( ( ( faecal OR fecal OR anal OR bowel ) W/2 ( incontinen* OR leak* OR soiling ) ) ) OR ABS ( ( ( faecal OR fecal OR anal OR bowel ) W/2 ( incontinen* OR leak* OR soiling ) ) ) ) ) OR ( ( TITLE ( ( belch* OR burp* OR eructation OR hiccup* ) ) OR ABS ( ( belch* OR burp* OR eructation OR hiccup* ) ) ) ) OR ( ( TITLE ( ( nausea* OR vomit* OR emesis OR retching ) ) OR ABS ( ( nausea* OR vomit* OR emesis OR retching ) ) ) ) OR ( ( TITLE ( ( reflux OR gerd OR dyspepsia OR indigestion OR heartburn OR regurgitat* ) ) OR ABS ( ( reflux OR gerd OR dyspepsia OR indigestion OR heartburn OR regurgitat* ) ) ) ) OR ( ( TITLE ( ( ( abdom* OR stomach OR epigastric OR rectal OR rectum OR belly ) W/2 ( pain* OR cramp* OR ache* OR colic OR discomfort ) ) ) OR ABS ( ( ( abdom* OR stomach OR epigastric OR rectal OR rectum OR belly ) W/2 ( pain* OR cramp* OR ache* OR colic OR discomfort ) ) ) ) ) OR ( ( TITLE ( ( ( gurgling OR rumbling ) W/2 ( abdom* OR stomach OR gastro* ) ) ) OR ABS ( ( ( gurgling OR rumbling ) W/2 ( abdom* OR stomach OR gastro* ) ) ) ) ) OR ( ( TITLE ( ( bloating OR bloated OR gassiness OR gaseousness OR flatulence OR flatulent OR flatus OR ( abdom* W/1 disten* ) OR ( swollen W/1 abdom* ) OR ( swelling W/2 abdom* ) OR ( postprandial W/1 fullness ) OR ( post-prandial W/1 fullness ) ) ) OR ABS ( ( bloating OR bloated OR gassiness OR gaseousness OR flatulence OR flatulent OR flatus OR ( abdom* W/1 disten* ) OR ( swollen W/1 abdom* ) OR ( swelling W/2 abdom* ) OR ( postprandial W/1 fullness ) OR ( post-prandial W/1 fullness ) ) ) ) ) OR ( ( TITLE ( ( incomplete W/1 evacuation ) ) OR ABS ( ( incomplete W/1 evacuation ) ) ) ) OR ( ( TITLE ( ( ( bowel* OR defecat* ) W/2 ( frequen* OR urgen* OR infrequen* ) ) ) OR ABS ( ( ( bowel* OR defecat* ) W/2 ( frequen* OR urgen* OR infrequen* ) ) ) ) ) OR ( ( TITLE ( ( ( loose OR watery ) W/1 stool* ) ) OR ABS ( ( ( loose OR watery ) W/1 stool* ) ) ) ) OR ( ( TITLE ( ( diarrh* OR constipat* ) ) OR ABS ( ( diarrh* OR constipat* ) ) ) ) OR ( ( TITLE ( ( irritable W/1 bowel ) ) OR ABS ( ( irritable W/1 bowel ) ) ) ) OR ( ( TITLE ( crohn* ) OR ABS ( crohn* ) ) ) OR ( ( TITLE ( ( ulcerative W/1 colitis ) ) OR ABS ( ( ulcerative W/1 colitis ) ) ) ) OR ( ( TITLE ( inflammatory W/1 bowel ) OR ABS ( inflammatory W/1 bowel ) ) ) OR ( ( TITLE ( ibs ) OR ABS ( ibs ) ) ) OR ( ( TITLE ( ( ( digestive OR gastro* ) W/3 symptom* ) ) OR ABS ( ( ( digestive OR gastro* ) W/3 symptom* ) ) ) ) OR ( ( TITLE ( ( ( digestive OR bowel OR gut OR gastro* OR colonic ) W/2 ( symptom* OR habit* OR issue* OR issue* OR problem* OR dysfunction* OR complaint* ) ) ) OR ABS ( ( ( digestive OR bowel OR gut OR gastro* OR colonic ) W/2 ( symptom* OR habit* OR issue* OR issue* OR problem* OR dysfunction* OR complaint* ) ) ) ) ) ) AND ( ( TITLE ( menopaus* OR perimenopaus* OR peri-menopaus* OR postmenopaus* OR post-menopaus* OR postreproductive OR post-reproductive OR climacteric ) OR ABS ( menopaus* OR perimenopaus* OR peri-menopaus* OR postmenopaus* OR post-menopaus* OR postreproductive OR post-reproductive OR climacteric ) ) )

Web of Science Core Collection

Search completed: 4^th^ March 2024

#1: TS=(((digestive or bowel or gut or gastro* or colonic) NEAR/2 (symptom* or habit* or issue* or issue* or problem* or dysfunction* OR complaint*))) 47544

#2: ((digestive or gastro*) NEAR/3 symptom*) (Topic) 28975

#3: IBS (Topic) 16390

#4: (inflammatory bowel) (Topic) 116400

#5: (ulcerative colitis) (Topic) 73709

#6: crohn* (Topic) 91202

#7: (irritable bowel) (Topic) 28092

#8: (diarrh* or constipat*) (Topic) 160025

#9: ((loose or watery) NEAR/1 stool*) (Topic) 1607

#10: ((bowel* or defecat*) NEAR/2 (frequen* or urgen* or infrequen*)) (Topic) 3416

#11: (incomplete NEAR/1 evacuation) (Topic) 476

#12: (bloating or bloated or gassiness or gaseousness or flatulence or flatulent or flatus or (abdom* NEAR/1 disten*) or (swollen NEAR/1 abdom*) or (swelling NEAR/2 abdom*) or (postprandial NEAR/1 fullness) or (post-prandial NEAR/1 fullness)). (Topic) 19942

#13: ((gurgling or rumbling) NEAR/2 (abdom* or stomach or gastro*)) (Topic) 46

#14: ((abdom* or stomach or epigastric or rectal or rectum or belly) NEAR/2 (pain* or cramp* or ache* or colic or discomfort)) (Topic) 75395

#15: (reflux or GERD or dyspepsia or indigestion or heartburn or regurgitat*) (Topic) 163632

#16 (belch* or burp* or eructation or hiccup*) (Topic) 4771

#17: (nausea* or vomit* or emesis or retching) (Topic) 99555

#18: ((faecal or fecal or anal or bowel) NEAR/2 (incontinen* or leak* or soiling)) (Topic) 13539

#19: ((bowel* NEAR/1 control*) or encopresis) (Topic) 1627

#20: ((gut or digestive or gastro* or bowel* or colon*) NEAR/2 health*) (Title) 3680

#21: #1 OR #2 OR #3 OR #4 OR #5 OR #6 OR #7 OR #8 OR #9 OR #10 OR #11 OR #12 OR #13 OR #14 OR #15 OR #16 OR #17 OR #18 OR #19 OR #20 697257

#22: TS=((menopaus* or perimenopaus* or peri-menopaus* or postmenopaus* or post-menopaus* or postreproductive or post-reproductive or climacteric)) 150503

#23: #21 AND #22 2224

ProQuest Dissertations & Theses

Search completed: 4^th^ March 2024

(title((menopaus* OR perimenopaus* OR peri-menopaus* OR postmenopaus* OR post-menopaus* OR postreproductive OR post-reproductive OR climacteric)) OR abstract((menopaus* OR perimenopaus* OR peri-menopaus* OR postmenopaus* OR post-menopaus* OR postreproductive OR post-reproductive OR climacteric))) AND ((title(((digestive OR bowel OR gut OR gastro* OR colonic) NEAR/2 (symptom* OR habit* OR issue* OR issue* OR problem* OR dysfunction* OR complaint*))) OR abstract(((digestive OR bowel OR gut OR gastro* OR colonic) NEAR/2 (symptom* OR habit* OR issue* OR issue* OR problem* OR dysfunction* OR complaint*)))) OR (title(((digestive OR gastro*) NEAR/3 symptom*)) OR abstract(((digestive OR gastro*) NEAR/3 symptom*))) OR (title(IBS) OR abstract(IBS)) OR (title((inflammatory bowel)) OR abstract((inflammatory bowel))) OR (title((ulcerative colitis)) OR abstract((ulcerative colitis))) OR (title(crohn*) OR abstract(crohn*)) OR (title((irritable bowel)) OR abstract((irritable bowel))) OR (title((diarrh* OR constipat*)) OR abstract((diarrh* OR constipat*))) OR (title(((loose OR watery) NEAR/1 stool*)) OR abstract(((loose OR watery) NEAR/1 stool*))) OR (title(((bowel* OR defecat*) NEAR/2 (frequen* OR urgen* OR infrequen*))) OR abstract(((bowel* OR defecat*) NEAR/2 (frequen* OR urgen* OR infrequen*)))) OR (title((incomplete evacuation)) OR abstract((incomplete evacuation))) OR (title((bloating OR bloated OR gassiness OR gaseousness OR flatulence OR flatulent OR flatus OR (abdom* NEAR/1 disten*) OR (swollen NEAR/1 abdom*) OR (swelling NEAR/2 abdom*) OR (postprandial NEAR/1 fullness) OR (post-prandial NEAR/1 fullness))) OR abstract((bloating OR bloated OR gassiness OR gaseousness OR flatulence OR flatulent OR flatus OR (abdom* NEAR/1 disten*) OR (swollen NEAR/1 abdom*) OR (swelling NEAR/2 abdom*) OR (postprandial NEAR/1 fullness) OR (post-prandial NEAR/1 fullness)))) OR (title(((gurgling OR rumbling) NEAR/2 (abdom* OR stomach OR gastro*))) OR abstract(((gurgling OR rumbling) NEAR/2 (abdom* OR stomach OR gastro*)))) OR (title(((abdom* OR stomach OR epigastric OR rectal OR rectum OR belly) NEAR/2 (pain* OR cramp* OR ache* OR colic OR discomfort))) OR abstract(((abdom* OR stomach OR epigastric OR rectal OR rectum OR belly) NEAR/2 (pain* OR cramp* OR ache* OR colic OR discomfort)))) OR (title((reflux OR GERD OR dyspepsia OR indigestion OR heartburn OR regurgitat*)) OR abstract((reflux OR GERD OR dyspepsia OR indigestion OR heartburn OR regurgitat*))) OR (title((belch* OR burp* OR eructation OR hiccup*)) OR abstract((belch* OR burp* OR eructation OR hiccup*))) OR (title((nausea* OR vomit* OR emesis OR retching)) OR abstract((nausea* OR vomit* OR emesis OR retching))) OR (title(((faecal OR fecal OR anal OR bowel) NEAR/2 (incontinen* OR leak* OR soiling))) OR abstract(((faecal OR fecal OR anal OR bowel) NEAR/2 (incontinen* OR leak* OR soiling)))) OR (title(((bowel* NEAR/1 control*) OR encopresis)) OR abstract(((bowel* NEAR/1 control*) OR encopresis))) OR title(((gut OR digestive OR gastro* OR bowel* OR colon*) NEAR/2 health*)))

Supplementary search methods

ClinicalTrials.gov (available at <https://clinicaltrials.gov>)

| **Search terms** | **Date searched** | **Number retrieved by search** | **Number of records exported** |
| --- | --- | --- | --- |
| Perimenopause \| Other terms: Gastrointestinal Symptoms | 17/04/2024 | 2 | 0 |
| Perimenopause \| Other terms: Constipation | 17/04/2024 | 0 | 0 |
| Perimenopause \| Other terms: Diarrhoea | 17/04/2024 | 1 | 0 |
| Perimenopause \| Other terms: Diarrhea | 17/04/2024 | 1 | 0 |
| Perimenopause \| Other terms: Nausea and Vomiting | 17/04/2024 | 2 | 0 |
| Perimenopause \| Other terms: Nausea | 17/04/2024 | 2 | 0 |
| Perimenopause \| Other terms: Vomiting | 17/04/2024 | 2 | 0 |
| Perimenopause \| Other terms: Fecal Incontinence | 17/04/2024 | 0 | 0 |
| Perimenopause \| Other terms: Gastroesophageal Reflux | 17/04/2024 | 0 | 0 |
| Perimenopause \| Other terms: Indigestion | 17/04/2024 | 0 | 0 |
| Perimenopause \| Other terms: Heartburn | 17/04/2024 | 1 | 0 |
| Perimenopause \| Other terms: Abdominal Pain | 17/04/2024 | 0 | 0 |
| Perimenopause \| Other terms: Abdominal Discomfort | 17/04/2024 | 0 | 0 |
| Perimenopause \| Other terms: Abdominal Distention | 17/04/2024 | 0 | 0 |
| Perimenopause \| Other terms: Bloating | 17/04/2024 | 0 | 0 |
| Perimenopause \| Other terms: Flatulence | 17/04/2024 | 1 | 0 |
| Perimenopausal Disorder \| Other terms: Gastrointestinal Symptoms | 17/04/2024 | 1 | 0 |
| Perimenopausal Disorder \| Other terms: Constipation | 17/04/2024 | 0 | 0 |
| Perimenopausal Disorder \| Other terms: Diarrhoea | 17/04/2024 | 0 | 0 |
| Perimenopausal Disorder \| Other terms: Diarrhea | 17/04/2024 | 0 | 0 |
| Perimenopausal Disorder \| Other terms: Nausea and Vomiting | 17/04/2024 | 1 | 0 |
| Perimenopausal Disorder \| Other terms: Nausea | 17/04/2024 | 1 | 0 |
| Perimenopausal Disorder \| Other terms: Vomiting | 17/04/2024 | 1 | 0 |
| Perimenopausal Disorder \| Other terms: Fecal Incontinence | 17/04/2024 | 1 | 0 |
| Perimenopausal Disorder \| Other terms: Gastroesophageal Reflux | 17/04/2024 | 0 | 0 |
| Perimenopausal Disorder \| Other terms: Indigestion | 17/04/2024 | 0 | 0 |
| Perimenopausal Disorder \| Other terms: Heartburn | 17/04/2024 | 0 | 0 |
| Perimenopausal Disorder \| Other terms: Abdominal Pain | 17/04/2024 | 0 | 0 |
| Perimenopausal Disorder \| Other terms: Abdominal Discomfort | 17/04/2024 | 0 | 0 |
| Perimenopausal Disorder \| Other terms: Abdominal Distention | 17/04/2024 | 0 | 0 |
| Perimenopausal Disorder \| Other terms: Bloating | 17/04/2024 | 0 | 0 |
| Perimenopausal Disorder \| Other terms: Flatulence | 17/04/2024 | 0 | 0 |
| Menopause \| Other terms: Gastrointestinal Symptoms | 17/04/2024 | 21 | 2 |
| Menopause \| Other terms: Constipation | 17/04/2024 | 6 | 2 |
| Menopause \| Other terms: Diarrhoea | 17/04/2024 | 7 | 1 |
| Menopause \| Other terms: Diarrhea | 17/04/2024 | 7 | 1 |
| Menopause \| Other terms: Nausea and Vomiting | 17/04/2024 | 5 | 0 |
| Menopause \| Other terms: Nausea | 17/04/2024 | 11 | 0 |
| Menopause \| Other terms: vomiting | 17/04/2024 | 6 | 0 |
| Menopause \| Other terms: Fecal Incontinence | 17/04/2024 | 3 | 1 |
| Menopause \| Other terms: Gastroesophageal Reflux | 17/04/2024 | 3 | 0 |
| Menopause \| Other terms: Indigestion | 17/04/2024 | 4 | 0 |
| Menopause \| Other terms: Heartburn | 17/04/2024 | 3 | 0 |
| Menopause \| Other terms: Abdominal Pain | 17/04/2024 | 11 | 0 |
| Menopause \| Other terms: Abdominal Discomfort | 17/04/2024 | 5 | 0 |
| Menopause \| Other terms: Abdominal Distention | 17/04/2024 | 7 | 1 |
| Menopause \| Other terms: Bloating | 17/04/2024 | 2 | 1 |
| Menopause \| Other terms: Flatulence | 17/04/2024 | 0 | 0 |
| Menopausal Women \| Other terms: Gastrointestinal Symptoms | 17/04/2024 | 16 | 2 |
| Menopausal Women \| Other terms: Constipation | 17/04/2024 | 5 | 1 |
| Menopausal Women \| Other terms: Diarrhea | 17/04/2024 | 6 | 0 |
| Menopausal Women \| Other terms: Diarrhoea | 17/04/2024 | 6 | 0 |
| Menopausal Women \| Other terms: Nausea and Vomiting | 17/04/2024 | 5 | 0 |
| Menopausal Women \| Other terms: Nausea | 13/06/2024 | 8 | 0 |
| Menopausal Women \| Other terms: Vomiting | 13/06/2024 | 5 | 0 |
| Menopausal Women \| Other terms: Fecal incontinence | 13/06/2024 | 3 | 1 |
| Menopausal Women \| Other terms: Gastroesophageal Reflux | 13/06/2024 | 3 | 0 |
| Menopausal Women \| Other terms: Indigestion | 13/06/2024 | 2 | 0 |
| Menopausal Women \| Other terms: Heartburn | 14/06/2024 | 2 | 0 |
| Menopausal Women \| Other terms: Abdominal Pain | 14/06/2024 | 9 | 0 |
| Menopausal Women \| Other terms: Abdominal Discomfort | 14/06/2024 | 3 | 0 |
| Menopausal Women \| Other terms: Abdominal Distention | 14/06/2024 | 6 | 0 |
| Menopausal Women \| Other terms: Bloating | 14/06/2024 | 1 | 0 |
| Menopausal Women \| Other terms: Flatulence | 14/06/2024 | 1 | 1 |
| Postmenopause \| Other terms: Gastrointestinal Symptoms | 14/06/2024 | 1 | 0 |
| Postmenopause \| Other terms: Constipation | 14/06/2024 | 0 | 0 |
| Postmenopause \| Other terms: Diarrhea | 14/06/2024 | 0 | 0 |
| Postmenopause \| Other terms: Diarrhoea | 14/06/2024 | 0 | 0 |
| Postmenopause \| Other terms: Nausea and Vomiting | 14/06/2024 | 0 | 0 |
| Postmenopause \| Other terms: Nausea | 14/06/2024 | 0 | 0 |
| Postmenopause \| Other terms: Vomiting | 14/06/2024 | 0 | 0 |
| Postmenopause \| Other terms: Fecal Incontinence | 14/06/2024 | 1 | 1 |
| Postmenopause \| Other terms: Gastroesophageal Reflux | 14/06/2024 | 0 | 0 |
| Postmenopause \| Other terms: Indigestion | 14/06/2024 | 0 | 0 |
| Postmenopause \| Other terms: Heartburn | 14/06/2024 | 2 | 0 |
| Postmenopause \| Other terms: Abdominal Pain | 14/06/2024 | 2 | 0 |
| Postmenopause \| Other terms: Abdominal Discomfort | 14/06/2024 | 1 | 0 |
| Postmenopause \| Other terms: Abdominal Distention | 14/06/2024 | 2 | 0 |
| Postmenopause \| Other terms: Bloating | 14/06/2024 | 0 | 0 |
| Postmenopause \| Other terms: Flatulence | 14/06/2024 | 1 | 1 |
| Postmenopausal Women \| Other terms: Gastrointestinal Symptoms | 14/06/2024 | 20 | 1 |
| Postmenopausal Women \| Other terms: Constipation | 14/06/2024 | 8 | 0 |
| Postmenopausal Women \| Other terms: Diarrhea | 14/06/2024 | 12 | 0 |
| Postmenopausal Women \| Other terms: Diarrhoea | 14/06/2024 | 12 | 0 |
| Postmenopausal Women \| Other terms: Nausea and Vomiting | 14/06/2024 | 10 | 0 |
| Postmenopausal Women \| Other terms: Nausea | 14/06/2024 | 12 | 0 |
| Postmenopausal Women \| Other terms: vomiting | 14/06/2024 | 10 | 0 |
| Postmenopausal Women \| Other terms: Fecal Incontinence | 14/06/2024 | 2 | 1 |
| Postmenopausal Women \| Other terms: Gastroesophageal Reflux | 14/06/2024 | 1 | 0 |
| Postmenopausal Women \| Other terms: Indigestion | 14/06/2024 | 4 | 1 |
| Postmenopausal Women \| Other terms: Heartburn | 14/06/2024 | 7 | 0 |
| Postmenopausal Women \| Other terms: Abdominal Pain | 14/06/2024 | 13 | 0 |
| Postmenopausal Women \| Other terms: Abdominal Discomfort | 14/06/2024 | 6 | 0 |
| Postmenopausal Women \| Other terms: Abdominal Distention | 14/06/2024 | 3 | 0 |
| Postmenopausal Women \| Other terms: Bloating | 14/06/2024 | 2 | 0 |
| Postmenopausal Women \| Other terms: Flatulence | 14/06/2024 | 2 | 0 |
| **Total** |  |  | **19** |

WHO International Clinical Trials Registry Platform (ICTRP) (available at: https://www.who.int/clinical-trials-registry-platform)

Searches completed: 14^th^ June 2024

**Title:** perimenopause or perimenopausal or menopause or menopausal or postmenopause or postmenopausal or climacteric AND

**Condition:** gastrointestinal or digestive or constipation or diarrhoea or diarrhea or nausea or vomiting or "faecal incontinence" or "fecal incontinence" or "anal incontinence" or "abdominal pain" or "abdominal discomfort" or "abdominal distention" or bloating or flatulence or "irritable bowel" or reflux or heartburn or indigestion or bowel. Identified 8 records. 0 downloaded.

Google (www.google.co.uk)

| **Search term(s)** | **Date searched** | **Number of records retrieved; number of records exported** |
| --- | --- | --- |
| Perimenopause “gastrointestinal symptoms” filetype:pdf | 13/05/2024 | 1170 results; more results x 10; exported 2 records |
| Perimenopause constipation filetype:pdf | 13/05/2024 | 5960; more results x 10; exported 5 records |
| Perimenopause diarrhoea filetype:pdf | 13/05/2024 | 1830; more results x 10; exported 1 record |
| Perimenopause diarrhea filetype:pdf | 13/05/2024 | 4420; more results x 10; exported 0 records |
| Perimenopause nausea filetype:pdf | 13/05/2024 | 9710; more results x 10; exported 0 records |
| Perimenopause vomiting filetype:pdf | 13/05/2024 | 6410; Google would not display more than first page; exported 0 records |
| Perimenopause “abdominal pain” filetype:pdf | 13/05/2024 | 6250; Google would not display 10 pages; exported 1 record |
| Perimenopause “fecal incontinence” filetype:pdf | 13/05/2024 | 649; more results x 10; exported 0 records |
| Perimenopause faecal incontinence filetype:pdf | 13/05/2024 | 3850; Google would not display 10 pages; exported 0 records |
| Perimenopause bloating filetype:pdf | 13/05/2024 | 32,800; Google would not display 10 pages; exported 0 records |
| Perimenopause reflux filetype:pdf | 13/05/2024 | 3460; more results x 10; exported 0 records |
| Perimenopause indigestion filetype:pdf | 13/05/2024 | 1280; more results x 10; exported 0 records |
| Perimenopause heartburn filetype:pdf | 13/05/2024 | 1980; more results x 10; exported 0 records |
| Menopause “gastrointestinal symptoms” filetype:pdf | 13/05/2024 | 17,200; more results x 10; exported 2 records |
| Menopause constipation filetype:pdf | 13/05/2024 | 92,800; more results x 10; exported 0 records |
| Menopause diarrhoea filetype:pdf | 13/05/2024 | 36,300; more results x 10; exported 0 records |
| Menopause diarrhea filetype:pdf | 13/05/2024 | 88,600; more results x 10; exported 0 records |
| Menopause nausea filetype:pdf | 13/05/2024 | 129,000; more results x 10; exported 0 records |
| Menopause vomiting filetype:pdf | 13/05/2024 | 135,000; more results x 10; exported 0 records |
| Menopause “abdominal pain” filetype:pdf | 13/05/2024 | 82,800; more results x 10; exported 0 records |
| Menopause “fecal incontinence” filetype:pdf | 13/05/2024 | 11,100; Google would not display 10 pages; exported 0 records |
| Menopause “faecal incontinence” filetype:pdf | 13/05/2024 | 6380; Google would not display 10 pages; exported 0 records |
| Menopause bloating filetype:pdf | 13/05/2024 | 62,100; more results x 10; exported 0 records |
| Menopause reflux filetype:pdf | 13/05/2024 | 60,000; more results x 10; exported 0 records |
| Menopause indigestion filetype:pdf | 14/05/2024 | 43,400; more results x 10; exported 0 records |
| Menopause heartburn filetype:pdf | 14/05/2024 | 30,300; more results x 10; exported 0 records |
| Postmenopausal “gastrointestinal symptoms” filetype:pdf | 14/05/2024 | 28,700; more results x 10; exported 0 records |
| Postmenopausal constipation filetype:pdf | 14/05/2024 | 79,500; more results x 10; exported 0 records |
| Postmenopausal diarrhoea filetype:pdf | 14/05/2024 | 35,600; Google would not display 10 pages; exported 0 records |
| Postmenopausal diarrhea filetype:pdf | 14/05/2024 | 82,800; more results x 10; exported 0 records |
| Postmenopausal nausea filetype:pdf | 14/05/2024 | 128,000; Google would not display 10 pages; exported 0 records |
| Postmenopausal vomiting filetype:pdf | 14/05/2024 | 102,000; Google would not display 10 pages; exported 0 records |
| Postmenopausal “abdominal pain” filetype:pdf | 14/05/2024 | 75,000; Google would not display 10 pages; exported 0 records |
| Postmenopausal “fecal incontinence” filetype:pdf | 14/05/2024 | 11,200; Google would not display 10 pages; exported 0 records |
| Postmenopausal “faecal incontinence” filetype:pdf | 14/05/2024 | 7440; Google would not display 10 pages; exported 0 records |
| Postmenopausal bloating filetype:pdf | 14/05/2024 | 27,700; Google would not display 10 pages; exported 0 records |
| Postmenopausal reflux filetype:pdf | 14/05/2024 | 48,200; Google would not display 10 pages; exported 0 records |
| Postmenopausal indigestion | 14/05/2024 | 13,900; Google would not display 10 pages; exported 0 records |
| Postmenopausal heartburn | 14/05/2024 | 13,500; Google would not display 10 pages; exported 0 records |
| Total |  | 11 |

Google Scholar (<https://scholar.google.com>)

| **Search terms** | **Date searched** | **Number of records retrieved; number of records exported** |
| --- | --- | --- |
| Perimenopause gastrointestinal symptoms | 8 May 2024 | 14700; screened 10 pages; exported 8 records |
| Perimenopause constipation | 8 May 2024 | 6310; screened 10 pages; exported 11 records |
| Perimenopause diarrhoea | 8 May 2024 | 1650; screened 10 pages; exported 4 records |
| Perimenopause diarrhea | 8 May 2024 | 5580; screened 10 pages; exported 2 records |
| Perimenopause nausea | 8 May 2024 | 10,800; screened 10 pages; exported 1 record |
| Perimenopause vomiting | 8 May 2024 | 7050; screened 10 pages; exported 0 records |
| Perimenopause abdominal pain | 8 May 2024 | 5960; screened 10 pages; exported 1 record |
| Perimenopause fecal incontinence | 8 May 2024 | 4600; screened 10 pages; exported 9 records |
| Perimenopause faecal incontinence | 9 May 2024 | 2070; screened 10 pages; exported 0 records |
| Perimenopause bloating | 9 May 2024 | 4610; screened 10 pages; exported 10 records |
| Perimenopause reflux | 9 May 2024 | 3300; screened 10 pages; exported 5 records |
| Perimenopause indigestion | 9 May 2024 | 977; screened 10 pages; exported 3 records |
| Perimenopause heartburn | 9 May 2024 | 1580; screened 10 pages; exported 0 records |
| Menopause gastrointestinal symptoms | 9 May 2024 | 79,800; screened 10 pages; exported 2 records |
| Menopause constipation | 9 May 2024 | 33,900; screened 10 pages; exported 7 records |
| Menopause diarrhoea | 9 May 2024 | 19,800; screened 10 pages; exported 4 records |
| Menopause diarrhea | 9 May 2024 | 30,200; screened 10 pages; exported 1 record |
| Menopause nausea | 9 May 2024 | 68,300; screened 10 pages; exported 3 records |
| Menopause vomiting | 9 May 2024 | 42,300; screened 10 pages; exported 0 records |
| Menopause “abdominal pain” | 9 May 2024 | 37300; screened 10 pages; exported 1 |
| Menopause “fecal incontinence” | 9 May 2024 | 8570; screened 10 pages; exported 7 records |
| Menopause “faecal incontinence” | 9 May 2024 | 4110; screened 10 pages; exported 4 records |
| Menopause bloating | 9 May 2024 | 21,000; screened 10 pages; exported 3 records 2 + Bairy |
| Menopause reflux | 9 May 2024 | 25,700; screened 10 pages; exported 1 record |
| Menopause indigestion | 9 May 2024 | 12,000; screened 10 pages; exported 2 records |
| Menopause heartburn | 9 May 2024 | 8140; screened 10 pages; exported 3 records |
| Postmenopausal “gastrointestinal symptoms” | 9 May 2024 | 16,300; screened 10 pages; exported 2 records |
| Postmenopausal constipation | 9 May 2024 | 30,200; screened 10 pages; exported 2 records |
| Postmenopausal diarrhoea | 9 May 2024 | 11,400; screened 10 pages; exported 0 records |
| Postmenopausal diarrhea | 9 May 2024 | 35,600; screened 10 pages; exported 0 records |
| Postmenopausal nausea | 9 May 2024` | 54,600; screened 10 pages; exported 0 records |
| Postmenopausal vomiting | 9 May 2024 | 37,800; screened 10 pages; exported 0 records |
| Postmenopausal “abdominal pain” | 9 May 2024 | 31,000; screened 10 pages; exported 0 records |
| Postmenopausal “fecal incontinence” | 9 May 2024 | 8,580 screened 10 pages; exported 2 records |
| Postmenopausal “faecal incontinence” | 11 May 2024 | 4130; screened 10 pages; exported 2 records |
| Postmenopausal bloating | 11 May 2024 | 13,000; screened 10 pages; exported 4 records |
| Postmenopausal reflux | 11 May 2024 | 21,700; screened 10 pages; exported 0 records |
| Postmenopausal indigestion | 11 May 2024 | 4700; screened 10 pages; exported 0 records |
| Postmenopausal heartburn | 11 May 2024 | 8600; screened 10 pages; exported 2 records |
| **Total** |  | **103** |

British Menopause Society (available from <https://thebms.org.uk>)

Searches completed: 11th May 2024

| **Search term** | **Number of records identified/exported** |
| --- | --- |
| Gastrointestinal | 0 |
| Constipation | 0 |
| Diarrhoea | 0 |
| Nausea | 1; 0 records exported |
| Vomiting | 0 |
| Abdominal pain | 1; 0 records exported |
| Faecal incontinence | 0; |
| Bloating | 1; 0 records exported |
| Reflux | 0 records |
| Indigestion | 0 |
| Heartburn | 0 records |

North American Menopause Society (available from <https://www.menopause.org/>)

Searches completed: 11th May 2024

| **Search term** | **Number of records identified/exported** |
| --- | --- |
| Gastrointestinal | 3; 0 records kept |
| Constipation | 1; 0 records kept |
| Diarrhea | 2; 0 records kept |
| Nausea | 7; 0 records kept |
| Vomiting | 2; 0 records kept |
| Abdominal pain | 5; 0 records |
| Fecal incontinence | 2; 0 |
| Bloating | 4; 0 |
| Reflux | 2; 0 |
| Indigestion | 1; 0 kept |
| Heartburn | 4; 0 kept |

International Menopause Society (available from: <https://www.imsociety.org>)
Searches completed: 14^th^ May 2024

| **Search terms** | **Number of records identified/exported** |
| --- | --- |
| Gastrointestinal | 10; 0 kept |
| Constipation | 2; 0 kept |
| Diarrhea | 1; 0 kept |
| Nausea | 2; 0 kept |
| Vomiting | 0 |
| Abdominal pain | 2; 0 kept |
| Fecal incontinence | 1; 0 kept |
| Bloating | 2; 0 kept |
| Reflux | 1; 0 kept |
| Indigestion | 0 |
| Heartburn | 0 |

Australasian Menopause Society (available from <https://www.menopause.org.au>)
Searches completed: 14^th^ May 2024

| **Search terms – keywords; all words** | **Number of records identified/exported** |
| --- | --- |
| Gastrointestinal | 13; 0 kept |
| Constipation | 6; 0 kept |
| Diarrhoea | 2; 0 kept |
| Nausea | 8; 0 kept |
| Vomiting | 4; 0 kept |
| Abdominal pain | 8; 0 kept |
| Faecal incontinence | 1; 0 kept |
| Bloating | 4; 0 kept |
| Reflux | 2; 0 kept |
| Indigestion | 0 |
| Heartburn | 1; 0 kept |

European Menopause and Andropause Society (available from: https://emas-online.org/)

Searches completed 14^th^ May 2024

| **Search term(s)** | **Number of records identified/exported** |
| --- | --- |
| Gastrointestinal | 1; 0 kept |
| Constipation | 0 |
| Diarrhea | 1; 0 kept |
| Nausea | 1; 0 kept |
| Vomiting | 0 |
| “Abdominal pain” | 2; 0 kept |
| Faecal incontinence | 7; 0 kept |
| Fecal incontinence | 2; 0 kept |
| Bloating | 0 |
| Reflux | 0 |
| Indigestion | 0 |
| Heartburn | 0 |
