# Supplementary material for: The volume and characteristics of research on gastrointestinal symptoms in ‘natural’ peri- and postmenopause: A scoping review
Source: Womens Health (Lond). 2025 Oct 27;21:17455057251387470. doi: 10.1177/17455057251387470 (PMC12575958; doi:10.1177/17455057251387470)
Supplement: sj-docx-4-whe-10.1177_17455057251387470 – Supplemental material for The volume and characteristics of research on gastrointestinal symptoms in ‘natural’ peri- and postmenopause: A scoping review [file sj-docx-4-whe-10.1177_17455057251387470.docx]

Supplemental Appendix 4. Excluded studies with reasons for exclusion

N=189 [total excluded studies from database searches, web searches, citation chasing and trial registers]

Foreign language (n=26)

1. Zhu MX, Huang Q, Wang F, et al. Changes in serum melatonin and estrogen levels in women with functional dyspepsia. *World Chin J Digestol* 2015; 23: 3038-3044.

2. Walecka E, Klupińska G, Chojnacki J, et al. Clinical aspects of chronic dyspepsia in postmenopausal women. *Prz Menopauzalny* 2003; 2: 53-58.

3. Qi DJ, He YN, Yu XS, et al. Clinical characteristics of perimenopausal inpatients in department of general practice in a tertiary hospital. *Chin Gen Pract* 2017; 20: 2373-2377.

4. Carranza-Lira S, Bn QG, Hc AG, et al. Comparison of climacteric symptoms among women in Mexico City and women of a Mayan community of Yucatan. *Ginecol Obstet Mex* 2012; 80: 644-649.

5. De Oliveira SCM, Pinto-Neto AM, Conde DM, et al. Constipation in postmenopausal women. *Rev Assoc Med Bras* 2005; 51: 334-341.

6. Stute P. Decreased hearing ability and constipation as symptoms of menopause?. [German]. *J Gynakol Endokrinol* 2018; 28: 105-106.

7. Wisniewska-Jarosinska M, Chojnacki J, Pawlowicz M, et al. Diagnostic value of measuring the amount of 6-hydroxymelatonin sulfate in urine in postmenopausal women with functional abdominal pain [Polish]. *Prz Menopauzalny* 2009; 8: 251-256.

8. Wu J, Chen BH and Zhuang JS. Differences of physical and mental symptoms, depressive emotion and sex hormone level in serum of women with climacteric syndrome of different traditional Chinese medicine syndromes. [Chinese]. *Chin J Clin Rehab* 2006; 10: 22-24.

9. Stute P. Does menopause promote constipation?. [German]. *Gynakol Endokrinol* 2018; 16: 130-131.

10. Wachowska-Kelly P, Walecka-Kapica E, Wojtkiewicz P, et al. [Efficacy of sulpiride and itopride in the treatment of functional dyspepsia in women with emotional and eating disorders]. *Pol Merkur Lekarski* 2014; 37: 39-42.

11. Roche B, Chautems R, Rakotoarimanana R, et al. Epidemiology of anal incontinence. [German]. *Chir Gastroenterol Interdis* 2002; 18: 282-285.

12. Mik M, Narbutt P, Tchorzewski M, et al. Faecal incontinence in postmenopausal women with vaginal delivery history. [Polish]. *Prz Menopauzalny* 2009; 13: 149-154.

13. De Oliveira SCM, Pinto-Neto AM, Conde DM, et al. Fecal incontinence in postmenopausal women: Prevalence, severity and associated factors. [Portuguese]. *Arq Gastroenterol* 2006; 43: 102-106.

14. Zutshi M, Hull TL, Bast J, et al. Female bowel function: The real story. [German]. *Coloproctology* 2007; 29: 195-204.

15. Adjoussou SA, Bohoussou E, Bastide S, et al. [Functional symptoms and associations of women with genital prolapse]. *Prog Urol* 2014; 24: 511-517.

16. Eskicioğlu M and Özdemir A. *Menopoz döneminde karşılaşılan sikâyetlerde alternatif tedavi kullanma durumlarının tespit edilmesi*. Bursa Uludag University, Turkey, Turkey, 2020.

17. Curi LA and Genoud MT. [Most frequent causes of fecal incontinence in our environment]. *Acta Gastroenterol Latinoam* 2000; 30: 165-168.

18. Cingi ME and Kömürcü N. *Postmenopozal kadınlarda anal inkontinans görülme sıklığı, etiyolojisi ve tedavisinde pelvik taban egzersizlerinin uygulanması*. Marmara Universitesi, Turkey, 2013.

19. Aldana Calva E and Mota Gonzalez C. Presence, severity and frequency of symptoms in post-menopause women patients of the National Institute of Perinatology. *Psicologia y Salud* 2020; 30: 207-216.

20. De Oliveira SCM, Pinto-Neto AM, Goes JRN, et al. Prevalence and factors associated with intestinal constipation in postmenopausal women. [Portuguese]. *Arq Gastroenterol* 2005; 42: 24-29.

21. Laudański P, Eljasiewicz E and Knapp P. Przewlek³y ból miednicy u kobiet w wieku menopauzalnym. *Prz Menopauzalny* 2012; 3: 187-191.

22. Ji AD and Guo YY. Revised Tongxie Yaofang for regulating IBS automatic nerve system function of women in menopause. *Shenzhen J Integ Trad Chin West Med* 2008; 18: 380‐383.

23. Wisniewska-Jarosinska M, Klupinska G, Kulig G, et al. Serotonin secretion and metabolism in postmenopausal women with gastrointestinal functional disorders. [Polish]. *Prz Menopauzalny* 2009; 13: 239-243.

24. Kamali K, Tehrani SG, Banaei M, et al. A survey of prevalence of constipation and its associated factors in postmenopausal Women referred to health centers of roudan -2015. [Persian]. *Iran J Obstet Gynecol Infertil* 2017; 20: 25-35.

25. Zhang ZL. Treatment of menopausal women with functional dyspepsia using Shugan Yangxin Decoction: An analysis of 57 cases. [Chinese]. *World Chin J Digestol* 2009; 17: 1795-1798.

26. Souza SSD, Santos RLD, Santos ADFD, et al. Woman and climaterio: conceptions of users of a basic health unit. *Reprod Climaterio* 2017; 32: 85-89. DOI:

Duplicate references (n=3)

1. Irct2016043010324N. The effect of herbal mixture capsule on constipation and sexual function of postmenopausal women with chronic constipation, https://trialsearch.who.int/Trial2.aspx?TrialID=IRCT2016043010324N32 (2016, accessed 3 July 204).

2. NCT05378009. Functional dyspepsia response to relaxation therapy and physical activity, https://clinicaltrials.gov/show/NCT05378009 (2022, accessed 3 July 2024).

3. NCT04352062. Melatonin supplementation in postmenopausal women with H. pylori-associated dyspepsia, https://clinicaltrials.gov/show/NCT04352062 (2020, accessed 3 July 2024).

Population (not individuals experiencing ‘natural’ perimenopause, menopause or postmenopause) (n=115)

1. Beutel ME, Weidner K, Schwarz R, et al. Age-related complaints in women and their determinants based on a representative community study. *Eur J Obstet Gynecol Reprod Biol* 2004; 117: 204-212.

2. Bardel A, Wallander MA, Wedel H, et al. Age-specific symptom prevalence in women 35-64 years old: A population-based study. *BMC Public Health* 2009; 9: 8.

3. Okeahialam NA, Thakar R, Ilczyszyn A, et al. Anal and urinary incontinence in nulliparous women–Prevalence and associated risk factors. *Post Reprod Health* 2021; 27: 89-97.

4. Rojas RG, Salvesen KÅ and Volløyhaug I. Anal sphincter defects and fecal incontinence 15-24 years after first delivery: a cross-sectional study. *Ultrasound Obstet Gynecol* 2018; 51: 677-683.

5. Pinedo G, Garcia E, Zarate AJ, et al. Are topical oestrogens useful in faecal incontinence? Double-blind randomized trial. *Colorectal Dis* 2009; 11: 390-393.

6. Oberwalder M, Dinnewitzer A, Baig MK, et al. The association between late-onset fecal incontinence and obstetric anal sphincter defects. *Arch Surg* 2004; 139: 429-432.

7. Cardenas-Trowers O, Meyer I, Richter HE, et al. Association of urinary phytoestrogens with pelvic organ prolapse and fecal incontinence symptoms in postmenopausal women. *Female Pelvic Med Reconstr Surg* 2019; 25: 161-166.

8. Li C, Borgfeldt C, Samsioe G, et al. Background factors influencing somatic and psychological symptoms in middle-age women with different hormonal status. A population-based study of Swedish women. *Maturitas* 2005; 52: 306-318.

9. Bharucha AE, Zinsmeister AR, Schleck CD, et al. Bowel disturbances are the most important risk factors for late onset fecal incontinence: A population-based case-control study in women. *Gastroenterol* 2010; 139: 1559-1566.

10. Triadafilopoulos G, Finlayson MA and Grellet C. Bowel dysfunction in postmenopausal women. *Women Health* 1998; 27: 55-66.

11. Harari D, Gurwitz JH, Avorn J, et al. Bowel habit in relation to age and gender: Findings from the National Health Interview Survey and clinical implications. *Arch Intern Med* 1996; 156: 315-320.

12. Stake-Nilsson K, Hultcrantz R, Unge P, et al. Changes in symptoms and lifestyle factors in patients seeking healthcare for gastrointestinal symptoms: an 18-year follow-up study. *Eur J Gastroenterol Hepatol* 2013; 25: 1470-1477.

13. Ziller V, Oppermann TS, Cassel W, et al. Chronic cough in postmenopausal women and its associations to climacteric symptoms. *BMC Womens Health* 2023; 23: 9.

14. Lacima G, Espuña M, Pera M, et al. Clinical, urodynamic, and manometric findings in women with combined fecal and urinary incontinence. *Neurourol Urodyn* 2002; 21: 464-469.

15. Nelson RL, Norton N, Cautley E, et al. Community-based prevalence of anal incontinence. *JAMA* 1995; 274: 559-561.

16. van Gerwen M, Schellevis F and Lagro-Janssen T. Comorbidities associated with urinary incontinence: A case-control study from the second dutch national survey of general practice. *J Am Board Fam Med* 2007; 20: 608-610.

17. Husain S and Singh M. A comparative study of perimenopausal and postmenopausal profile of working and non-working women. *Asian J Home Sci* 2013; 8: 631-635.

18. Soerensen MM, Buntzen S, Bek KM, et al. Complete obstetric anal sphincter tear and risk of long-term fecal incontinence: A cohort study. *Dis Colon Rectum* 2013; 56: 992-1001.

19. Chiarelli P, Brown W and McElduff P. Constipation in Australian women: prevalence and associated factors. *Int Urogynecol J* 2000; 11: 71-78.

20. Whalley B, Jacobs PA and Hyland ME. Correlation of psychological and physical symptoms with chronically elevated cytokine levels associated with a common immune dysregulation. *Ann Allergy Asthma Immunol* 2007; 99: 348-351.

21. Sze EH, Barker CD and Hobbs G. A cross-sectional survey of the relationship between fecal incontinence and constipation. *Int Urogynecol J* 2013; 24: 61-65.

22. Ohsawa M, Takayama S, Kikuchi A, et al. Daisaikoto for shoulder stiffness and related changes in stool condition: Retrospective study. *Trad Kampo Med* 2019; 6: 105-108.

23. Ai F, Deng M, Mao M, et al. Depressive symptoms screening in postmenopausal women with symptomatic pelvic organ prolapse. *Menopause* 2018; 25: 314-319.

24. Im E-O and Chee W. A descriptive Internet survey on menopausal symptoms: five ethnic groups of Asian American university faculty and staff. *J Transcult Nurs* 2005; 16: 126-135.

25. Ryva BA, Haggerty DK, Pacyga DC, et al. Determinants of urinary phthalate biomarker concentrations in pre- and perimenopausal women with consideration of race. *Environ Res* 2022; 214: 114056.

26. Georges JM. *Distressing gastrointestinal symptoms in postmenopausal women*. University of Washington, USA, 1992.

27. Georges JM. Distressing gastrointestinal symptoms in postmenopausal women. *Commun Nurs Res* 1992; 25: 241-246.

28. O'Connor VM, Del Mar CB, Sheehan M, et al. Do psycho-social factors contribute more to symptom reporting by middle-aged women than hormonal status? *Maturitas* 1994; 20: 63-69.

29. Eogan M, O'Brien C, Daly L, et al. The dual influences of age and obstetric history on fecal continence in parous women. *Int J Gynecol Obstet* 2011; 112: 93-97.

30. Kuutti MA, Hyvärinen M, Kauppinen M, et al. Early adulthood and current physical activity and their association with symptoms of pelvic floor disorders in middle-aged women: An observational study with retrospective physical activity assessment. *BJOG* 2023; 130: 664-673.

31. Irct20190220042774N. Effect of Descurainia sophia (L.) seed Webb ex Prantl on the severity of functional constipation in women 50-70 years old, https://trialsearch.who.int/Trial2.aspx?TrialID=IRCT20190220042774N1 (2019, accessed 3 July 2024).

32. Bach FL, Sairally BZF and Latthe P. Effect of oestrogen therapy on faecal incontinence in postmenopausal women: a systematic review. *Int Urogynecol J* 2020; 31: 1289-1297.

33. Botros SM, Abramov Y, Miller JJ, et al. Effect of parity on sexual function: an identical twin study. *Obstet Gynecol* 2006; 107: 765-770.

34. Ryhammer AM, Laurberg S and Sorensen FH. Effects of age on anal function in normal women. *Int J Colorectal Dis* 1997; 12: 225-229.

35. Zheng Z, Margolis KL, Liu S, et al. Effects of estrogen with and without progestin and obesity on symptomatic gastroesophageal reflux. *Gastroenterol* 2008; 135: 72-81.

36. Azimi M, Niayesh H, Raeiszadeh M, et al. Efficacy of the herbal formula of Foeniculum vulgare and Rosa damascena on elderly patients with functional constipation: A double-blind randomized controlled trial. *J Integr Med-JIM* 2022; 20: 230-236.

37. Lacima G, Pera M, Valls-Sole J, et al. Electrophysiologic studies and clinical findings in females with combined fecal and urinary incontinence: a prospective study. *Dis Colon Rectum* 2006; 49: 353-359.

38. Li Z, Xu T, Li Z, et al. An epidemiologic study of pelvic organ prolapse in postmenopausal women: a population-based sample in China. *Climacteric* 2019; 22: 79-84.

39. Chinzon D, Dias-Bastos TRP, da Silva AM, et al. Epidemiology of constipation in Sao Paulo, Brazil: a population-based study. *Curr Med Res Opin* 2015; 31: 57-64.

40. do Rosario Dias de Oliveira Latorre M, Medeiros da Silva A, Chinzon D, et al. Epidemiology of upper gastrointestinal symptoms in Brazil (EpiGastro): a population-based study according to sex and age group. *World J Gastroenterol* 2014; 20: 17388-17398.

41. Im E-O. Ethnic differences in symptoms experienced during the menopausal transition. *Health Care Women Int* 2009; 30: 339-355.

42. Huerta-Franco M-R, Banderas JW and Allsworth JE. Ethnic/racial differences in gastrointestinal symptoms and diagnosis associated with the risk of Helicobacter pylori infection in the US. *Clin Exp Gastroenterol* 2018; 11: 39-49.

43. Camtosun A, Sen I, Onaran M, et al. An evaluation of fecal incontinence in women with urinary incontinence. *Eur Rev Med Pharmacol Sci* 2016; 20: 1918-1922.

44. Craig BM and Mitchell SA. Examining the value of menopausal symptom relief among us women. *Value Health* 2016; 19: 158-166.

45. Cooney MC. *The experience and knowledge of menopause among low-income Cleveland women.* Case Western Reserve University, USA, 2002.

46. Moore B, Gustafson R and Studd J. Experience of a national health service menopause clinic. *Curr Med Res Opin* 1975; 3: 42-55.

47. Bartlett L, Nowak M and Ho Y-H. Faecal incontinence in rural and regional northern Queensland community-dwelling adults. *Rural Remote Health* 2013; 13: 2563-2563.

48. Horng SS, Chou YJ, Huang N, et al. Fecal incontinence epidemiology and help seeking among older people in Taiwan. *Neurourol Urodyn* 2013; 33: 1153-1158.

49. Melville JL, Fan MY, Newton KM, et al. Fecal incontinence in US women: A population-based study. *Am J Obstet Gynecol* 2005; 193: 2071-2076.

50. Jackson SL, Weber AM, Hull TL, et al. Fecal incontinence in women with urinary incontinence and pelvic organ prolapse. *Obstet Gynecol* 1997; 89: 423-427.

51. Wang JY, Patterson TR, Hart SL, et al. Fecal incontinence: Does age matter? Characteristics of older vs. younger women presenting for treatment of fecal incontinence. *Dis Colon Rectum* 2008; 51: 426-431.

52. Zutshi M, Hull TL, Bast J, et al. Female bowel function: the real story. *Dis Colon Rectum* 2007; 50: 351-358.

53. Williams RE, Kalilani L, DiBenedetti DB, et al. Frequency and severity of vasomotor symptoms among peri- and postmenopausal women in the United States. *Climacteric* 2008; 11: 32-43.

54. Fialkow MF, Melville JL, Lentz GM, et al. The functional and psychosocial impact of fecal incontinence on women with urinary incontinence. *Am J Obstet Gynecol* 2003; 189: 127-129.

55. Cain KC, Jarrett ME, Burr RL, et al. Gender differences in gastrointestinal, psychological, and somatic symptoms in irritable bowel syndrome. *Dig Dis Sci* 2009; 54: 1542-1549.

56. Kim SY, Jung HK, Lim J, et al. Gender specific differences in prevalence and risk factors for gastro-esophageal reflux disease. *J Korean Med Sci* 2019; 34: e158.

57. Li CR, Wilawan K, Samsioe G, et al. Health profile of middle-aged women: The Women's Health in the Lund Area (WHILA) study. *Hum Reprod* 2002; 17: 1379-1385.

58. Williams RE, Kalilani L, DiBenedetti DB, et al. Healthcare seeking and treatment for menopausal symptoms in the United States. *Maturitas* 2007; 58: 348-358.

59. Berecki-Gisolf J, Spallek M, Hockey R, et al. Height loss in elderly women is preceded by osteoporosis and is associated with digestive problems and urinary incontinence. *Osteoporos Int* 2010; 21: 479-485.

60. Kane SV and Reddy D. Hormonal replacement therapy after menopause is protective of disease activity in women with inflammatory bowel disease. *Am J Gastroenterol* 2008; 103: 1193-1196.

61. Duffy O, Iversen L and Hannaford PC. The impact and management of symptoms experienced at midlife: a community-based study of women in northeast Scotland. *BJOG* 2012; 119: 554-564.

62. Rolston VS, Boroujerdi L, Long MD, et al. The influence of hormonal fluctuation on inflammatory bowel disease symptom severity-a cross-sectional cohort study. *Inflamm Bowel Dis* 2018; 24: 387-393.

63. Kremsjka A. The influence of menopausal symptoms on women’s sex life. *GinPolMedProject* 2020; 3.

64. Donnelly V, Oconnell PR and Oherlihy C. The influence of oestrogen replacement on faecal incontinence in postmenopausal women. *Br J Obstet Gynaecol* 1997; 104: 311-315.

65. Sherburn M, Guthrie JR, Dudley EC, et al. Is incontinence associated with menopause? *Obstet Gynecol* 2001; 98: 628-633.

66. Brown WJ, Mishra G, Lee C, et al. Leisure time physical activity in Australian women: Relationship with well being and symptoms. *Res Q Exerc Sport* 2000; 71: 206-216.

67. Everist R, Burrell M, Parkin K, et al. The long-term prevalence of anal incontinence in women with and without obstetric anal sphincter injuries*. Continence* 2023; 5: 100571.

68. Ford K, Sowers M, Crutchfield M, et al. A longitudinal study of the predictors of prevalence and severity of symptoms commonly associated with menopause. *Menopause* 2005; 12: 308-317.

69. Everhart JE, Go VLW, Johannes RS, et al. A longitudinal survey of self-reported bowel habits in the United States. *Dig Dis Sci* 1989; 34: 1153-1162.

70. Mundhra R, Dhiman N, Chaturvedi J, et al. Measurement of menopausal symptoms using greene climacteric scale in a tertiary care centre in Uttarakhand, India. *J Clin Diagn Res* 2018; 12.

71. Chang C and Chang CH. Menopause and hormone using experiences of Chinese women in Taiwan. *Health Care Women Int* 1996; 17: 307-318.

72. Pimenta F. *Menopause and midlife: menopausal symptoms, body weight and well-being.* ISPA-Instituto Universitário, Lisbon, Portugal, 2011.

73. Bohle B, Belvis F, Vial M, et al. Menopause and obstetric history as risk factors for fecal incontinence in women. Dis Colon Rectum 2011; 54: 975-981.

74. Lindemann E. Modifications in the course of ulcerative colitis in relationship to changes in life situations and reaction patterns. *Res Publ Assoc Res Nerv Ment Dis* 1949; 29: 706-723.

75. Nilsson M, Johnsen R, Ye W, et al. Obesity and estrogen as risk factors for gastroesophageal reflux symptoms. *J Am Med Assoc* 2003; 290: 66-72.

76. ISRCTN75311338. An oestrogen cream for the treatment of faecal incontinence, https://trialsearch.who.int/Trial2.aspx?TrialID=ISRCTN75311338 (2008, accessed 3 July 2024).

77. Harper JC, Phillips S, Biswakarma R, et al. An online survey of perimenopausal women to determine their attitudes and knowledge of the menopause. *Womens Health* 2022; 18: 17455057221106890.

78. Richter HE, Morgan SL, Gleason JL, et al. Pelvic floor symptoms and bone mineral density in women undergoing osteoporosis evaluation. *Int Urogynecol J* 2013; 24: 1663-1669.

79. Azzeddine S, Jahida C, Abdelkader D, et al. Perimenopausal symptoms, quality of life and eating behavior in west Algerian women. *IMJ Health* 2017; 3: 70-76.

80. MacLellan J, Dixon S, Bi S, et al. Perimenopause and/or menopause help-seeking among women from ethnic minorities: a qualitative study of primary care practitioners’ experiences. *Br J Gen Pract* 2023; 73: e511-e518.

81. Sievert LL and Bertone-Johnson E. Perimenstrual symptoms and symptoms at midlife in Puebla, Mexico. *Climacteric* 2013; 16: 169-178.

82. Lenhart A, Naliboff B, Shih W, et al. Postmenopausal women with irritable bowel syndrome (IBS) have more severe symptoms than premenopausal women with IBS. *Neurogastroenterol Motil* 2020; 32: e13913.

83. Murad-Regadas SM, Regadas FSP, Regadas Filho FSP, et al. Predictors of unsuccessful of treatment for fecal incontinence biofeedback for fecal incontinence in female. *Arq Gastroenterol* 2019; 56: 61-65.

84. Hage-Fransen MAH, Wiezer M, Otto A, et al. Pregnancy- and obstetric-related risk factors for urinary incontinence, fecal incontinence, or pelvic organ prolapse later in life: A systematic review and meta-analysis. *Acta Obstet Gynecol Scand* 2020; 100: 373-382.

85. Andrews E, Eaton SC, Hollis K, et al. Prevalence and demographics of irritable bowel syndrome: results from a large web-based survey. *Aliment Pharmacol Ther* 2005; 22: 935-942.

86. Tougas G, Chen Y, Hwang P, et al. Prevalence and impact of upper gastrointestinal symptoms in the Canadian population: Findings from the DIGEST study. *Am J Gastroenterol* 1999; 94: 2845-2854.

87. Infantino M. The prevalence and pattern of gastroesophageal reflux symptoms in perimenopausal and menopausal women. *J Am Acad Nurse Pract* 2008; 20: 266-272.

88. Gallas S, Frioui S, Rabeh H, et al. Prevalence and risk factors for urinary and anal incontinence in Tunisian middle aged women. Afr J Urol 2018; 24: 368-373.

89. Rommen K, Schei B, Rydning A, et al. Prevalence of anal incontinence among Norwegian women: a cross-sectional study. *BMJ Open* 2012; 2: 1-9.

90. Rømmen K, Schei B, Rydning A, et al. Prevalence of anal incontinence among Norwegian women: a cross-sectional study. *BMJ Open* 2012; 2: 1-9.

91. Aslan E, Beji NK, Erkan HA, et al. The prevalence of and the related factors for urinary and fecal incontinence among older residing in nursing homes. *J Clin Nurs* 2009; 18: 3290-3298.

92. Botlero R, Bell RJ, Urquhart DM, et al. Prevalence of fecal incontinence and its relationship with urinary incontinence in women living in the community. *Menopause* 2011; 18: 685-689.

93. Ho KY, Kang JY and Seow A. Prevalence of gastrointestinal symptoms in a multiracial Asian population, with particular reference to reflux-type symptoms. *Am J Gastroenterol* 1998; 93: 1816-1822.

94. Batur AF, Onaran M, Sen I, et al. Prevalence of urinary tract infections in women with urinary incontinence and other risk factors. *J Clin Anal Med* 2017; 8: 145-149.

95. Yu Q, Chae H-D, Hsiao S-M, et al. Prevalence, severity, and associated factors in women in East Asia with moderate-to-severe vasomotor symptoms associated with menopause. *Menopause* 2022; 29: 553-563.

96. Yang K, Tabung FK, Whitehead WE, et al. Proinflammatory diet is associated with increased risk of fecal incontinence among older women: Prospective results from the Nurses' Health Study. *Clin Gastroenterol Hepatol* 2023; 21: 1657-1659.e1653.

97. Turnbull GK, Thompson DG, Day S, et al. Relationships between symptoms, menstrual cycle and orocaecal transit in normal and constipated women. *Gut* 1989; 30: 30-34.

98. Abramov Y, Sand PK, Botros SM, et al. Risk factors for female anal incontinence: New insight through the Evanston-Northwestern Twin Sisters Study. *Obstet Gynecol* 2005; 106: 726-732.

99. Bunyavejchevin S. Risk factors of female urinary incontinence and overactive bladder in Thai postmenopausal women. *J Med Assoc Thai* 2005; 88: S119-123.

100. Chu X and Stern MC. *Risk factors of pelvic floor disorders in the multiethnic cohort study.* MSc, University of Southern California, USA, 2018.

101. Kaur H, Bala R and Nagpal M. Role of Vitamin D in urogenital health of geriatric participants. *J Midlife Health* 2017; 8: 28-35.

102. Cameron A, Fenner DE, DeLancey JO, et al. Self-report of difficult defecation is associated with overactive bladder symptoms. *Neurourol Urodyn* 2010; 29: 1290-1294.

103. Oskay UY, Beji NK and Yalcin O. A study on urogenital complaints of postmenopausal women aged 50 and over. *Acta Obstet Gynecol Scand* 2005; 84: 72-78.

104. Nath A, Ahmed SJ, Saikia H, et al. A study to assess the psychosomatic problems of postmenopausal women in slums of Dibrugarh town, Assam. *Int J Contemp Med Res* 2017; 4: 407-410.

105. Park Y-J, Kim HS, Ku P-S, et al. A survey on the climacteric symptoms in Korean women. *Women Health* 2001; 34: 17-28.

106. Sievert LL and Obermeyer CM. Symptom clusters at midlife: a four-country comparison of checklist and qualitative responses. *Menopause* 2012; 19: 133-144.

107. Beketie ED, Tafese WT, Assefa ZM, et al. Symptomatic pelvic floor disorders and its associated factors in South-Central Ethiopia. *PLoS One* 2021; 16: e0254050.

108. Im E-O. Symptoms experienced during menopausal transition: Korean Women in South Korea and the United States. *J Transcult Nurs* 2003; 14: 321-328.

109. Nilsson I, Åkervall S, Molin M, et al. Symptoms of fecal incontinence two decades after no, one, or two obstetrical anal sphincter injuries. *Am J Obstet Gynecol* 2020; 224: e271-276.

110. Nagórska M, Sowa A, Wardak K, et al. Symptoms of menopause and health of women during perimenopause. *Eur J Clin Exp Med* 2018; 16: 259-266.

111. Huber G. Treatment of gastro-intestinal complaints accompanying menopause with Motilium film-coated tablet. *Ther Hung* 1987; 35: 227-231.

112. Versi E, Harvey MA, Cardozo L, et al. Urogenital prolapse and atrophy at menopause: A prevalence study. *Int Urogynecol J* 2001; 12: 107-110.

113. Bradley CS, Zimmerman MB, Wang Q, et al. Vaginal descent and pelvic floor symptoms in postmenopausal women: a longitudinal study. *Obstet Gynecol* 2008; 111: 1148-1153.

114. Bradley CS and Nygaard I. Vaginal wall descensus and pelvic floor symptoms in older women. *Obstet Gynecol* 2005; 106: 759-766.

115. Brigstocke S, Nee J, Ballou S, et al. Women's health factors and altered bowel habits: results of a National Health and Nutrition Examination Survey. *GastroHep* 2021; 3: 141-150.

Not a self-reported GI symptom (n=36)

1. Huang S, Wang Z, Zheng D, et al. Anxiety disorder in menopausal women and the intervention efficacy of mindfulness-based stress reduction. *Am J Translat Res* 2023; 15: 2016‐2024.

2. Kubota Y, Iso H and Tamakoshi A. Bowel movement frequency, laxative use, and mortality from coronary heart disease and stroke among Japanese men and women: The Japan Collaborative Cohort (JACC) study. *J Epidemiol* 2015; 26: 242-248.

3. Wang P-H, Li Y-C, Wu Y-H, et al. Clinical evaluation of Guilu Erxian Jiao in treating perimenopausal syndrome. *J Chin Med* 2012; 23: 165-181.

4. Irct201102215878N. Compareing effects of kinds of drugs on menopausal symptoms, https://trialsearch.who.int/Trial2.aspx?TrialID=IRCT201102215878N1 (2011, accessed 3 July 2024).

5. Irct2013072714174N. Comparison of the efficacy of citalopram and compound of Asperugo procumbens and foeniculum vulgare in treatment of menopausal disorders, https://trialsearch.who.int/Trial2.aspx?TrialID=IRCT2013072714174N1 (2014, accessed 3 July 2024).

6. Menon S, Prew S, Parkes G, et al. Do differences in female sex hormone levels contribute to gastro-oesophageal reflux disease? *Eur J Gastroenterol Hepatol* 2013; 25: 772-777.

7. Irct2012070210166N. Effect of Cornus mas fruit on menopausal symptoms in menopausal women refers to the Clinics of Rasool Akram Hospital, https://trialsearch.who.int/Trial2.aspx?TrialID=IRCT2012070210166N1 (2012, accessed 3 July 2024).

8. Vestergaard P, Hermann AP, Stilgren L, et al. Effects of 5 years of hormonal replacement therapy on menopausal symptoms and blood pressure-a randomised controlled study. *Maturitas* 2003; 46: 123‐132.

9. Van Die MD, Bone KM, Burger HG, et al. Effects of a combination of hypericum perforatum and vitex agnus-castus on PMS-like symptoms in late-perimenopausal women: Findings from a subpopulation analysis. *J Altern Complement Med* 2009; 15: 1045-1048.

10. Nie XF, Rong L, Yue SW, et al. Efficacy of community-based pelvic floor muscle training to improve pelvic floor dysfunction in chinese perimenopausal women: A randomized controlled trial. *J Community Health Nurs* 2021; 38: 48-58.

11. Slavin MN, Farmer S and Earleywine M. Expectancy mediated effects of marijuana on menopause symptoms. *Addict Res Theory* 2016; 24: 322-329.

12. Othman JA-M, Åkervall S, Nilsson IEK, et al. Fecal incontinence in nonpregnant nulliparous women aged 25 to 64 years-a randomly selected national cohort prevalence study. *Am J Obstet Gynecol* 2022; 226: 706. e701-706. e723.

13. Nakano M, Nakamura Y, Suzuki T, et al. Implications of historical height loss for prevalent vertebral fracture, spinal osteoarthritis, and gastroesophageal reflux disease. *Sci Rep* 2020; 10: 19036.

14. Menon S, Jayasena H, Nightingale P, et al. Influence of age and sex on endoscopic findings of gastrooesophageal reflux disease: an endoscopy database study. *Eur J Gastroenterol Hepatol* 2011; 23: 389-395.

15. Menon SS. *The influence of gender on the aetiology of gastro-oesophageal reflux, barrett’s oesophagus and oesophageal adenocarcinoma*. University of Birmingham, UK, 2011.

16. Martínez-Galiano JM, Peinado-Molina RA, Martínez-Vazquez S, et al. Influence of pelvic floor disorders on sexuality in women. *Int J Gynecol Obstet* 2024; 164: 1141-1150.

17. Hambisa HD, Birku Z and Gedamu S. Magnitude of symptomatic pelvic floor dysfunction and associated factors amongst women in western Ethiopia: A cross-sectional study. *Inquiry* 2023; 60: 8.

18. Punyahotra S, Dennerstein L and Lehert P. Menopausal experiences of Thai women. Part 1: Symptoms and their correlates. *Maturitas* 1997; 26: 1-7.

19. Nand SL, Webster MA, Baber R, et al. Menopausal symptom control and side-effects on continuous estrone sulfate and three doses of medroxyprogesterone acetate. *Climacteric* 1998; 1: 211-218.

20. NCT05677698. Menopausal symptoms and aromatherapy, https://clinicaltrials.gov/show/NCT05677698 (2022, accessed 3 July 2024).

21. Ho SC, Chan SG, Yip YB, et al. Menopausal symptoms and symptom clustering in Chinese women. *Maturitas* 1999; 33: 219-227.

22. Schwarz S, Völzke H, Alte D, et al. Menopause and determinants of quality of life in women at midlife and beyond: the Study of Health in Pomerania (SHIP). *Menopause* 2007; 14: 123-134.

23. Irct2015062122854N. Nigella sativa effects on the menopausal complications, https://trialsearch.who.int/Trial2.aspx?TrialID=IRCT2015062122854N1 (2016, accessed 3 July 2024).

24. Dumitrascu MC, Nenciu AE, Neacsu A, et al. Obesity impact on symptomatology associated with pelvic floor dysfunction. *Rom J Mil Med* 2021; 124: 339-343.

25. Lee YC, Karlamangla AS, Yu Z, et al. Pain severity in relation to the final menstrual period in a prospective multiethnic observational cohort: Results from the Study of Women's Health Across the Nation. *J Pain* 2017; 18: 178-187.

26. Hess R, Olshansky E, Ness RB, et al. Pregnancy and birth history influence women's experience of menopause. *Menopause* 2008; 15: 435-441.

27. Sueblinvong T, Taechakraichana N and Phupong V. Prevalence of climacteric symptoms according to years after menopause. *J Med Assoc Thai* 2001; 84: 1681-1691.

28. Karaçam Z, Özsoy S and Yurdal N. The prevalence of urinary incontinence and risk factors in menopausal women in Turkey: A systematic review and meta-analysis. *Int J Urol Nurs* 2023: 12.

29. Tabatabaeichehr M, Mortazavi H, Abadi MH, et al. Sexual desire and related factors in middle-aged and elderly married women: A cross-sectional study in Iran. *Open Access Maced J Med Sci* 2018; 6: 1906-1911.

30. Polit DF and Sa L. Social and psychological correlates of menopausal symptoms. *Psychosom Med* 1980; 42: 335-345.

31. Irct20170227032795N. Study of the effects of Elaeagnus angustifolia L. fruit on the profile of hormones and lipids and genital infectious in menopausal women, https://trialsearch.who.int/Trial2.aspx?TrialID=IRCT20170227032795N4 (2018, accessed 3 July 2024).

32. Leidy LE. Symptoms of menopause in relation to the timing of reproductive events and past menstrual experience. *Am J Hum Biol* 1996; 8: 761-769.

33. Berecki-Gisolf J, Begum N and Dobson AJ. Symptoms reported by women in midlife: menopausal transition or aging? *Menopause* 2009; 16: 1021-1029.

34. Wang HL, Tai MK, Hung HM, et al. Unique symptoms at midlife of women with osteoporosis and cardiovascular disease in Taiwan. *Menopause* 2013; 20: 315-321.

35. An J and Li L. Urban-rural differences in epidemiology and risk factors of menopause syndrome in middle-aged Chinese women. *Menopause* 2023; 30: 306-316.

36. Santoro N, Teal S, Gavito C, et al. Use of a levonorgestrel-containing intrauterine system with supplemental estrogen improves symptoms in perimenopausal women: a pilot study. *Menopause* 2015; 22: 1301-1307.

Publication type (n=9)

1. Nelson-Porter B. African women experiencing perimenopause: Bloating. A Paper Presented in Partial Fulfillment of the Requirements for the International PhD, http://www.brigettes.com/pdfs/Nelson-Porter_Africa_Paper_15(Bloating)_InternationalPhD_2014.pdf (2014, accessed 3 July 2024).

2. Haadem K. Anal sphincter competence in women; as related to delivery, tears, menopause and urinary incontinence. *Acta Obstet Gynecol Scand* 1992; 71: 251-252.

3. Kronemyer B. Are race, ethnicity, menopausal status linked to GI symptoms? *Contemp Ob Gyn* 2022; 67: 15-15.

4. Hlavinka E. How periods change with age. *New Sci* 2024; 261: 18-18.

5. Pensabene T. *Impact of a walking intervention on perimenopausal symptoms*. Texas Woman's University, USA, 1997.

6. Pines A. Menopause and irritable bowel syndrome. *Climacteric* 2010; 13: 606-607.

7. Bidmead J and Cardozo LD. Pelvic floor changes in the older woman. *Br J Urol* 1998; 82: 18-25.

8. Salamon JN, Mazurek J and Zolty R. Postmenopausal women with constipation and cardiovascular disease. *Am J Med* 2012; 125: e5; author reply e7-8.

9. Salmoirago-Blotcher E, Crawford S and Ockene I. Postmenopausal women with constipation and cardiovascular disease. Reply. *Am J Med* 2012; 125: E7-E8.

Articles not retrieved – unavailable from University Library services (n=3)

1. Errichi S, Bottari A, Belcaro G, et al. Supplementation with Pycnogenol® improves signs and symptoms of menopausal transition*. Panminerva Med* 2011; 53: 65‐70.

2. Praveena M*. A comparative study of premenopausal and postmenopausal women with GERD in relation to serum estrogen levels*. PSG Institute of Medical Sciences and Research, Coimbatore, India, 2020.

3. Wattanayingcharoenchai R, Manonai J, Vallibhakara SAO, et al. Prevalence and correlates of anal incontinence among urogynecologic patients. *J Med Assoc Thai* 2018; 101: 1031-1035.
