# Supplementary material for: The volume and characteristics of research on gastrointestinal symptoms in ‘natural’ peri- and postmenopause: A scoping review
Source: Womens Health (Lond). 2025 Oct 27;21:17455057251387470. doi: 10.1177/17455057251387470 (PMC12575958; doi:10.1177/17455057251387470)
Supplement: sj-docx-5-whe-10.1177_17455057251387470 – Supplemental material for The volume and characteristics of research on gastrointestinal symptoms in ‘natural’ peri- and postmenopause: A scoping review [file sj-docx-5-whe-10.1177_17455057251387470.docx]

Supplemental Appendix 5. Table of individual study characteristics

| **First author (Year)** | **Study design** | **Country (Region)** | **Menopausal stages studied** | **Criteria used to assess stage** | **GI symptoms studied** | **Tool used to assess GI symptoms** | **Timeframe**  **of symptom recall** | **Variables/interventions studied** |
| --- | --- | --- | --- | --- | --- | --- | --- | --- |
| *Adeyemo (2010)^25^ | Systematic review | N/A | PRE; POST | Not stated | Abdominal pain  Bloating  Constipation  Diarrhoea  Nausea  (IBS symptoms) | Not stated | Not stated | Menopausal stage (PRE vs POST) |
| *Ali Ismail (2022)^86^ | RCT | Egypt  (Africa) | PERI | Not stated | Dyspepsia (heartburn) | Glasgow Dyspepsia Severity Score;  Visual Analogue Scale to assess severity of abdominal symptoms | Not stated | Benson's relaxation therapy (BRT) + aerobic exercise vs Benson's relaxation therapy (BRT) only |
| Belcaro (2010)^93^ | RCT | Italy  (Europe) | PRE; PERI; POST | Age: recruitment >40yrs and <50yrs | Abdominal pain  Bloating | Menopause Symptoms Questionnaire (34 items) | Not stated | Afragil supplement (calcium, vitamin D, antioxidants) |
| *Chojnacki (2013)^102^ | Non-randomised trial | Poland  (Europe) | POST | Within 2-14 yrs of final menstrual period | Abdominal pain  Bloating  Constipation  Diarrhoea  (IBS symptoms) | Participant diary noting severity of symptoms (10-pt visual analogue scale) | Not stated | Melatonin vs placebo |
| *Chojnacki (2020)^101^ | Non-randomised trial | Poland  (Europe) | POST | Not stated | Dyspepsia  (heartburn) | Visual analogue scale | Not stated | Melatonin vs placebo |
| *Eliasvandi (2019)^110^ | RCT | Iran  (Asia) | POST | Last menstruation at least 12 mths prior | Constipation | Constipation questionnaire based on Rome III criteria;  Patient assessment constipation questionnaire (PAC-SYM) | Previous 2 wks | Herbal capsule (cloves; anise; anison; violet flowers; Terminalia chebula; green raisin; senna leaves; Alhagi maurorum; golgand) vs placebo |
| *Kumar (2017)^135^ | Non-randomised | India  (Asia) | PERI; POST | 5 menopausal symptoms; age 40-65yrs; irregularity in menstrual cycles | Constipation Diarrhoea | Physical Symptoms Checklist (2015) | Not stated | Dietary interventions: High protein; High Frequency; Traditional Weight loss vs no dietary intervention |
| *Lucas (2004)^143^ | RCT | USA  (North  America) | POST | Not stated | Constipation  Diarrhoea | Validated bowel movement questionnaire (7-pt scale assessing stool consistency; straining; pain during bowel movements; feelings of constipation) | 7-day diary | Dried plums (100g) vs dried apple (75g) |
| *Shamloufard (2017)^174^ | RCT | USA  (North America) | POST | Not stated | Constipation  Diarrhoea | Bowel movement questionnaire (7-pt scale assessing stool consistency; straining; pain during bowel movements; feelings of constipation) | 7-day diary | Dried plums (50 or 100g) vs no dried plums |
| Manuever Marketing  (2024)^80^ | Registration for RCT | USA  (North  America) | POST | Has not had a period in the last 12 consecutive mths | Bloating  Constipation  Diarrhoea | Bristol Stool Chart. Bloating measured with a study-specific scale (5-pt Likert scale) | Previous 3 mths | Provitalize Natural Menopause Probiotic (*Bifidobacterium breve, Lactobacillus Gasseri*, and *Bifidobacterium animalis subsp. Lactis)* vs placebo |
| Suman (2023)^81^ | Registration for RCT | India  (Asia) | PERI | Not stated | Bloating | Not stated | Not stated | Ayurvedic treatment (Guduchi,Yashtimadhu and Shankhpushpi) compound and Shatapushpa Basti. Comparator not stated. |
| Yoneda (2022)^82^ | Registration for RCT | Japan  (Asia) | PERI; POST | Not stated | Constipation  Diarrhoea | Izumo Scale for evaluating abdominal symptoms | Not stated | Lactic acid bacteria probiotic vs placebo |
| Abraham (1994)^83^ | Longitudinal | Australia  (Oceania) | PRE; POST | Not stated | Abdominal pain  Bloating  Constipation  Nausea | Study-specific tool | Not stated | Menopausal stage (PRE vs POST); Premenstrual symptoms |
| Adeniji (2023)^84^ | Cross-sectional | Nigeria  (Africa) | PRE; POST | Within the menopause age range (45-65yrs). Menopausal status self- reported | Bloating  Constipation | Not stated | Not stated | Body mass index |
| Agreus (1994)^85^ | Cross-sectional | Sweden  (Europe) | PRE; POST | Not stated | Abdominal symptoms (not specified) | Study-specific tool | Previous 3 mths | Menopausal stage (PRE vs POST) |
| Al-Badr (2022)^38^ | Cross-sectional | Saudi Arabia  (Asia) | PRE; POST | Not stated | Faecal incontinence | Pelvic Floor Distress Index (PFDI20) | Not stated | Menopausal stage (PRE vs POST) |
| Alshiek (2019)^87^ | Cross-sectional | USA  (North America) | PRE; POST | Age: recruited PRE: 18-40yrs; POST: 52-85yrs | Constipation  Diarrhoea  Faecal incontinence | Colorectal Anal Distress Inventory (CRADI-8); Pelvic Floor Distress Index (PFDI20) | Not stated | Menopausal stage (PRE vs POST) |
| Alwi (2009)^88^ | Cross-sectional | Malaysia  (Asia) | PRE; PERI; POST | STRAW. POST: no menstrual bleeding in previous 12 mths; Late PERI: menstruation in previous 2–12 mths but not last 2 mths; Early PERI: increasing irregularity of menses without skipped periods (7 days difference from the beginning of a given cycle to next); PRE: minor changes in cycle length. Early + late PERI transition stages grouped together. | Bloating | MENQOL | Previous 30 days | Menopausal stage (PRE vs PERI vs POST) |
| Aras (2023)^79^ | Cross-sectional; cluster analysis | Not stated; app-based study | PRE; PERI; POST | Self-reported. POST: ≥12 mths since last period; PERI: <12 mths since last period + hot flushes/night sweats; PRE: <12 mths since last period + no hot flushes/night sweats. | Abdominal pain  Bloating  Constipation  Diarrhoea  Nausea  Vomiting | Study-specific tool. Symptom logs from a list of 45 available symptoms on an app | Symptoms recorded as occurred | Menopausal stage (PRE vs PERI vs POST)  Associated menopausal symptoms |
| Ayranci (2010)^89^ | Cross-sectional | Turkey  (Asia) | PRE; PERI; POST-natural; POST-surgical | Adapted WHO classification. PRE: regular menstrual  bleeding within last 12 mths. PERl: irregular menses within last 12  mths or absence of menstrual bleeding for >3 mths but <12 mths. POST-natural: stopped menstrual bleeding spontaneously at least 1 yr. POST-surgical women: stopped periods due to medical/surgical intervention. | Bloating  Constipation  Diarrhoea  Heartburn | Study-specific tool based on Greene Climacteric Scale and other literature | Previous 6 mths or since age 40 | Menopausal stage (PRE vs PERI vs POST-natural vs POST-surgical) |
| Bapayeva (2023)^90^ | Cross-sectional | Kazakhstan (Asia) | PRE; PERI; POST | Self-reported.  PERI: ‘transitional phase’; POST: 12 mths amenorrhea in women older than  45 yrs | Bloating | MENQOL | Not stated | Age  Occupation (nurses vs doctors) |
| Barati (2021)^91^ | Cross-sectional | Iran  (Asia) | POST | Cessation of menstruation without medication for at least 12 mths | Bloating | MENQOL | Not stated | - |
| Barbosa (2019)^92^ | Longitudinal | Denmark  (Europe) | PRE; POST | Not stated | Faecal incontinence | Wexner Incontinence Scale; St Marks Score | Not stated | Menopausal stage (PRE vs POST); Years since menopause (PRE/<5yrs POST vs >5yrs POST) |
| *Bener (2008)^94^ | Cross-sectional | Qatar  (Asia) | ‘PRE’ | Not stated | Faecal incontinence | Study-specific tool | Previous 12 mths | Menopausal stage (PRE vs POST) |
| Bezerra (2014)^32^ | Cross-sectional | Brazil  (South America) | PRE; POST | Not stated | Constipation  Faecal incontinence | Study-specific tool. Questioned about presence of anal incontinence and defecatory issues | Not stated | Menopausal stage (PRE vs POST) |
| *Blonska (2023)^95^ | Case-control | Poland  (Europe) | POST | Last menstruation at least 2 yrs previous | Constipation | Study-specific tool. Severity of abdominal symptoms scored (7-pt scale) | Not stated | Age  Assay of sex hormones;  Other assays (urinary tryptophan; kynurenine, 3-indoxyl sulfate, 5-hydroxyindoleacetic acid; glomulerular filtration rate; alanine aminotransferase; aspartate aminotransferase; C-reaction protein; fecal calprotectin; hydrogen/methane concentration in exhaled air); Tryptophan intake  Body mass index |
| *Brimiene (2023)^96^ | Cross-sectional | Lithuania  (Europe) | PRE; PERI; POST | STRAW+10 (stages -3 to +2) | Constipation  Diarrhoea | Bristol Stool Chart (1-2=constipation; 6-7 diarrhoea) | Not stated | Associated menopausal symptoms |
| Brown (2002)^98^ | Longitudinal | Australia  (Oceania) | PRE; PERI; POST; POST-HRT | Self-report. PRE: menstrual bleeding in last 3 mths, and in last 12 mths with same frequency as in yr prior to that; PERI: menstrual bleeding in last 12 mths, but not in last 3 mths or with different menstrual frequency compared with previous yr; POST: no menstrual bleeding in last 12mths. | Constipation | Study-specific tool. Survey asked women about their experiences of 10 symptoms | Previous 12 mths | Menopausal stage (PRE vs PERI vs POST vs POST-HRT) |
| Brown (2009)^97^ | Cross-sectional | USA  (North America) | PRE; PERI | Between 45–54 yrs old, had at least 3 but <9 menstrual periods within past 12 mths. Not stated how it was determined from these participants who was PRE and PERI | Nausea | Study-specific tool | Not stated | Comorbidities (Depression as measured by Center for Epidemiologic Studies-  Depression Scale (CES-D)) |
| *Callan (2018)^31^ | Longitudinal | USA  (North America) | LRS (PRE); early MT; late MT (PERI); early POST | Using menstrual calendar data. Stages using Mitchell, Woods and Mariella; validated from ReSTAGE; names for stages from STRAW: LRS: cycles still regular; early MT: persistent irregularity of >6 days’ absolute difference between any 2 consecutive menstrual cycles in calendar yr, no skipped periods; Late MT: persistent skipping of ≥1 menstrual periods; Early POST: 5yrs after the final menstrual period (FMP). FMP retrospectively identified after 1yr unexplained amenorrhea. | Constipation  Diarrhoea | Study-specific tool. Health diary: rating of severity of constipation and diarrhoea (0-4 scale) | Previous 24 hrs | Age  Assays of sex hormones; Assays of stress hormones (cortisol, epinephrine, norepinephrine)  Menopausal stage (LRS vs early MT vs late MT vs early POST)  Comorbidities (stress-related perceptions: anxiety, tension, perceived stress) |
| *Callan (2019)^34^ | Longitudinal | USA  (North America) | LRS (PRE); early MT; late MT (PERI); early POST | Using menstrual calendar data. Stages using Mitchell, Woods and Mariella; validated from ReSTAGE; names for stages from STRAW: LRS: cycles still regular; early MT: persistent irregularity of >6 days’ absolute difference between any 2 consecutive menstrual cycles in calendar yr, no skipped periods; Late MT: persistent skipping of ≥1 menstrual periods; Early POST: 5yrs after the final menstrual period (FMP). FMP retrospectively identified after 1yr unexplained amenorrhea. | Abdominal pain | Study-specific tool. Health diary: abdominal pain severity (0-4 scale) | Previous 24 hrs | Age  Assays of sex hormones; Assays of stress hormones (cortisol, epinephrine, norepinephrine)  Menopausal stage (LRS vs early MT vs late MT vs early POST)  Comorbidities (stress-related perceptions: anxiety, tension, perceived stress) |
| Chang (2001)^99^ | Cross-sectional | USA  (North America) | PRE; POST | Not stated | Bloating  (IBS symptoms) | Bowel Symptom Questionnaire | Previous 4 wks | Menopausal stage (PRE vs POST) |
| Chen (2003)^100^ | Cross-sectional | Taiwan  (Asia) | PRE; POST | Not stated | Constipation  Faecal incontinence | Study-specific tool asking about presence of anal incontinence or constipation | Not stated | Menopausal stage (PRE vs POST) |
| Chen (2024)^29^ | Cross-sectional | USA  (North America) | PRE; POST | Not stated | Abdominal pain  Constipation  Diarrhoea  Heartburn  Nausea | Study-specific tool. Daily symptom diaries with items for GI symptoms rated on a 5-pt Likert scale | Diary kept for 28 consecutive days | Menopausal stage (PRE vs POST) |
| *Choghakhori (2017)^33^ | Cross-sectional | Iran  (Asia) | PRE; POST | Not stated | Abdominal pain  Bloating  Constipation  Diarrhoea  (IBS symptoms) | Visual Analog Scale (VAS) for abdominal pain/distention, dissatisfaction with bowel habits, and overall GI symptoms. IBS severity-scoring system (IBS-SSS) | Over a 10-day period | Menopausal stage (PRE vs POST) |
| Chung (2014)^103^ | Cross-sectional | South Korea  (Asia) | PERI | Not stated | Bloating | Study-specific tool including premenstrual symptoms | Not stated | Comorbidities (premenstrual symptoms) |
| Coslov (2021)^104^ | Cross-sectional | New Zealand; Canada; Mexico; UK; Australia; USA  (Multiple regions) | LRS (PRE); MT (PERI) | STRAW+10. LRS: Stage 3a:  ≥3 menses in past 3 mths experiencing changes in cycle length, flow, number of days. MT+: Stages -1 or -2: <3 periods in 3 mths. (Some women may have recently entered early POST). | Abdominal pain  Bloating  Constipation  Diarrhoea  Heartburn  Nausea | Study-specific tool. Developed from review of symptom scales. | Previous 6 mths | Menopausal stage (LRS vs MT+) |
| Coslov (2023)^105^ | Cross-sectional | Canada; USA  (North America) | LRS (PRE); MT (PERI) | STRAW+10 | Abdominal pain  Bloating  Constipation  Diarrhoea  Heartburn  Nausea | Study specific tool. 61 symptoms rated on degree of bother (1-8). | Not stated | “Not feeling like myself” |
| Deeks (2008)^106^ | Cross-sectional | Australia  (Oceania) | PRE; PERI; POST | Self-reported. PERI: 5-10yrs before menopause, during which women often but not always experience symptoms. Menopause: menstrual cycles cease. Normal during ages 45-55yrs. | Bloating  Constipation | Study-specific tool of 38-symptoms. | Not stated | Menopausal stage (PERI vs POST) |
| Dennerstein (1993)^108^ | Cross-sectional | Australia  (Oceania) | PRE; PERI; POST (natural); POST (surgical) | PRE: no changes in menstrual frequency/flow in prior 12 mths; PERI: changes in menstrual frequency/flow in prior 12 mths;  POST-natural: no menses in prior 12 mths; POST-surgical: hysterectomy and/or bilateral oophorectomy | Constipation  Diarrhoea | Study-specific tool. 22 symptom list. | Previous 2 wks | Menopausal stage (PRE vs PERI vs POST) |
| Dennerstein (2003)^107^ | Longitudinal | Australia  (Oceania) | PRE; early MT; late MT (PERI); POST | STRAW. PRE: no change in menstrual frequency; early MT: change in menstrual frequency but menses in past 3 mths; late MT: 3-11mths of amenorrhoea; POST: amenorrhoea for at least 12mths | Digestive symptoms (not specified) | Questionnaires developed by Kaufert & McKinlay | Not stated | Menopausal stage (MT vs no change in menopausal status) |
| Duffy (2013)^109^ | Cross-sectional | UK  (Europe) | PRE; PERI; POST | STRAW. PRE: menstrual periods not changed in the last few yrs; PERI: menstrual cycle irregularities but not  gone 12 mths without a period; POST: not had a period in last 12 mths; | Bloating | Study-specific tool. Checklist of 23 symptoms commonly associated with the menopause | Previous mth | Associated menopausal symptoms (hot flushes) |
| Fisher (2016)^111^ | Cross-sectional | USA  (North America) | PERI; POST | Not stated | Nausea | Study-specific tool. Digital diary assessing sensations accompanying hot flashes | 3-days of monitoring with digital diary | Associated menopausal symptoms (hot flushes) |
| Fukuda (2005)^112^ | Cross-sectional | Japan  (Asia) | PRE; PERI | Not stated | Constipation  Diarrhoea | Study-specific tool assessing self-reported defecation state | Not stated | Bowel habits before/during menstruation. |
| Geeta (2011)^27^ | Cross-sectional | India  (Asia) | Younger PRE; Older PRE; POST | Younger PRE: regular menstrual cycles and <40; older PRE: regular menstrual cycles and ≥40; POST: menstrual cycle ceased for at least 1 yr | Constipation  Diarrhoea  Heartburn | Study-specific tool | Not stated | Menopausal stage (younger PRE vs older PRE vs POST) |
| Hajdini (2017)^113^ | Cross-sectional | Albania  (Europe) | PERI; POST | Recruited by age (45-64yrs) | Bloating  Constipation | Study-specific tool | Not stated | - |
| *Hakimi (2020)^114^ | Cross-sectional | Iran  (Asia) | POST | Not stated | Faecal incontinence | Pelvic Floor Distress Inventory-20 (PFDI-20) | Not stated | Age |
| High (1994)^115^ | Cross-sectional | USA  (North America) | POST | "Women who had completed the menstrual cycle" | Bloating | Study-specific tool | Not stated | Occupation (managers vs non-managers) |
| Hilditch (1999)^116^ | Cross-sectional | Canada; China  (Multiple regions) | POST | Included women 2-7yrs after natural menopause. | Bloating | MENQOL | Not stated | Country/nationality (Chinese vs Canadian) |
| Huang (2017)^35^ | Cross-sectional | China | PRE; POST | Not stated | Constipation | Rome III criteria for chronic constipation | Previous 6 mths | Menopausal stage (PRE vs POST) |
| Huerta (1995) Symptoms at perimenopausal^117^ | Cross-sectional | Mexico  (North America) | PRE; early POST; late POST | PRE: last menses <12mths earlier; early POST: last menses >12 but < 24mths earlier; late POST: last menses >24 mths earlier | Digestive problems (not specified) | Study-specific tool; digestive problems | Not stated | - |
| Huerta (1995) Symptoms at the menopausal^118^ | Cross-sectional | Mexico  (North America) | PRE; early POST; late POST | PRE: last menses <12mths earlier; early POST: last menses >12 but < 24mths earlier; late POST: last menses >24 mths earlier | Digestive problems (not specified) | Study-specific tool; digestive problems included as a non-specific symptom of depression | Not stated | Menopausal stage/yrs since menopause (PRE vs early POST (12-23mths) vs late POST (2+yrs)) |
| Huerta-Franco (2019)^30^ | Cross-sectional | Mexico  (North America) | PRE; PERI; POST | “Based on  the criteria of previous studies”. Stages were  also determined by evaluating serum E2 and FSH levels. Cut-off points for menopausal women: FSH >40 IU/L and E2< 40 pg/mL | Abdominal pain  Constipation | Study-specific tool | Previous 3 mths | Menopausal stage (PRE vs PERI vs POST) |
| Im (2010) Menopausal^119^ | Cross-sectional | USA  (North America) | PRE; early PERI; late PERI; POST | Categories used from SWAN study. PRE: menses in previous 3 mths, no increase in irregularity; early PERI: menstrual bleeding in previous 3 mths, increasing irregularity in cycle length over past yr; late PERI: menses in previous 12 mths but not in previous 3 mths; POST: no menstrual bleeding in previous 12 mths (not due to medication, pregnancy, or severe weight loss) | Abdominal pain  Bloating  Diarrhoea | Midlife Women’s Symptom Index (MSI) 73-items | Not stated | Race/ethnicity |
| Im (2010) Subethnic ^120^ | Cross-sectional | USA  (North America) | PRE; early PERI; late PERI; POST | Determined using 7 items asking about last menstrual cycle, regularity, and flow. | Abdominal pain | Midlife Women’s Symptom Index (MSI) | Not stated | Subethnic group |
| Im (2017)^124^ | Cross-sectional; cluster analysis | USA  (North America) | PRE; PERI; POST | PRE: menstruation in previous 3 mths, with no change in irregularity; PERI: menstruation in previous 12 mths, + irregularity in cycle length; POST: no menstruation in past yr (not due to medication, pregnancy, or severe weight loss) | Bloating  Nausea  Heartburn | Cardiovascular Symptom Index for Midlife Women (CSIMW) | Not stated | Comorbidities (clusters with cardiovascular symptoms) Race/ethnicity |
| *Im (2021)^123^ | Cross-sectional | USA  (North America) | PRE; PERI; POST | PRE: menstruation in previous 3 mths, with no change in irregularity; PERI: menstruation in previous 12 mths, + irregularity in cycle length; POST: no menstruation in past yr (not due to medication, pregnancy, or severe weight loss) | Abdominal pain  Bloating  Constipation  Diarrhoea  Heartburn  Nausea  Vomiting | GI Symptom Index for Midlife Women (GIMW) | Not stated | Menopausal stage (PRE vs PERI vs POST)  Race/ethnicity |
| *Im (2022)^122^ | Cross-sectional | USA  (North America) | PRE; PERI; POST | PRE: menstruation in previous 3 mths, with no change in irregularity; PERI: menstruation in previous 12 mths, + irregularity in cycle length; POST: no menstruation in past yr (not due to medication, pregnancy, or severe weight loss) | Abdominal pain  Bloating  Constipation  Diarrhoea  Heartburn  Nausea  Vomiting | GI Symptom Index for Midlife Women (GIMW) | Not stated | Immigration status |
| *Im (2023)^121^ | Cross-sectional; cluster analysis | USA  (North America) | PRE; PERI; POST | PRE: menstruation during past 3 mths, with no change in regularity. PERI: menstruation during past 3 mths with increasing irregularity in cycle length in past 1 yr and those with menstruation during  past 12 mths, but not during previous 3 mths. POST: without menstruation in past yr | Abdominal pain  Bloating  Constipation  Diarrhoea  Heartburn  Nausea  Vomiting | GI Symptom Index for Midlife Women (GIMW) | Not stated | Menopausal stage (PRE+PERI vs POST)  Associated GI symptoms Race/ethnicity |
| Jia (2015)^125^ | Longitudinal | China  (Asia) | POST | Not stated | Constipation | Chinese Medical Symptom Scale (CMSS) at 4 time points | Not stated | Herbal supplement |
| Joh (2009)^126^ | Cross-sectional | South Korea  (Asia) | POST (<25yrs since menopause; >25yrs since menopause) | Not stated | Faecal incontinence | Rothenberger Scale | Previous 3 mths | Years since menopause (<25yrs vs >25yrs since menopause) |
| Joshi (2015)^127^ | Cross-sectional | India  (Asia) | PRE; PERI; POST | STRAW | Constipation | Menopause Rating Scale (MRS) | Not stated | - |
| Kang (2021)^128^ | Cross-sectional | India  (Asia) | POST | Not stated | Bloating | MENQOL | Not stated | - |
| *Kargin (2017)^129^ | Cross-sectional | Turkey  (Asia) | PRE; POST | PRE: 18-49yrs menstruating at least once in last 12 mths; POST: 49-70yrs with no history of menstruation in last 12 mths | Faecal incontinence | Wexner Incontinence Scale | Not stated | Menopausal stage (PRE vs POST)  Obstetric history (vaginal vs c-section delivery) |
| Karmarkar (2017)^130^ | Cross-sectional | India  (Asia) | PERI; POST | Recruited based on age (40-60yrs) | Bloating | MENQOL | Not stated | - |
| Kaur (2021)^131^ | Cross-sectional | India  (Asia) | POST | At least 1 yr of amenorrhea | Bloating  Nausea | Women’s Health Questionnaire | Not stated | - |
| *Kirka (2021)^132^ | Cross-sectional | Turkey  (Asia) | POST | Not having period in prior 12 mths | Constipation | Constipation Severity Instrument | Not stated | Quality of life (assessed with the Chronic Constipation Patient Assessment  Quality of Life Questionnaire (PAC-QOL)) |
| Koirala (2020)^133^ | Cross-sectional | Nepal  (Asia) | POST | Not stated | Bloating | MENQOL | Previous 30 days | - |
| Koo (2017)^134^ | Cross-sectional | South Korea | PERI; POST | STRAW. PERI (early transition): gap in normal menstrual period of >7 days). PERI (late transition): ≥2 skipped menstrual cycles or no menses for ≥60 days; POST: no menses for at least 12 mths. | Bloating | MENQOL | Not stated | Menopausal stage (PERI vs POST)  Body mass index (normal; overweight; obese according to WHO Asian BMI criteria) |
| Lee (2001)^136^ | Cross-sectional | USA  (North America) | PRE; POST | Not stated | Abdominal pain  Bloating  Constipation  Diarrhoea  Heartburn  Nausea  (IBS symptoms) | Bowel Symptom Questionnaire | Previous 4 wks | Menopausal stage (PRE vs POST; POST vs males) |
| Li (2000)^138^ | Cross-sectional | USA  (North America) | PERI | PERI: changes in menstrual pattern (irregularity or no menstruation for 3-11 mths) | Bloating | Women's Health Assessment Scale (WHAS) | Previous 12 mths | Distress associated with symptoms |
| Li (2016)^137^ | Cross-sectional | China  (Asia) | PERI | STRAW. Excluded women with amenorrhoea >1 yr | Constipation | Not stated | Not stated | Comorbidities (perimenopausal syndrome: depression/anxiety) |
| Li (2019)^139^ | Cross-sectional | China  (Asia) | PRE; PERI; POST | PRE: regular periods; PERI: irregular periods in last 12 mths;  POST: 12mths since last period | Constipation  Faecal incontinence | Adapted from International Consultation Incontinence Questionnaire-short form (ICIQ-SF) | Not stated | Comorbidities (stress urinary incontinence) |
| Lock (1988)^140^ | Cross-sectional | Japan  (Asia) | PRE; PERI; POST | PRE: regularly menstruating; PERI: menstrual cycle irregular in past yr, or menstruated in past 12mths, but not past 3mths; POST: not menstruated for 12mths | Abdominal pain  Constipation  Diarrhoea  Nausea | Modified version of General Symptom List (from Greenlick and Pope) to include additional menopausal symptoms | Previous 2 wks | - |
| Loutfy (2006)^141^ | Cross-sectional | Egypt  (Africa) | POST | Cessation of menstruation for ≥ 1 year | Bloating  Nausea | Study-specific tool | Not stated | Onset of menopause (slow vs rapid) |
| Lu (2016)^142^ | Cross-sectional | China  (Asia) | PRE; PERI; POST | Not stated | Constipation  Faecal incontinence | Not stated | Not stated | Comorbidities (stress urinary incontinence) |
| Luciano (2020)^144^ | Retrospective | France  (Europe) | PRE; POST; POST-HRT | Not stated | Faecal incontinence | Jorge and Wexner Incontinence Scale | Not stated | Menopausal stage (PRE vs POST vs POST-HRT) |
| Luptakova (2012) Menopausal^145^ | Cross-sectional | Slovakia  (Europe) | PRE; PERI; POST | PRE: regular menstruation during last 12mths. PERI: menstrual cycle length more irregular in preceding 12mths or stopped menstruating for between 3-12 mths; POST: 12 consecutive mths of amenorrhoea | Abdominal pain  Bloating  Nausea | Menopause-specific questionnaire, designed by Kaczmarek | Not stated | Menopausal stage (PRE vs PERI + POST)  Genetic markers (CYP1B1 Leu432Val polymorphism) |
| Luptakova (2012) The association^146^ | Cross-sectional | Slovakia  (Europe) | PRE; PERI; POST | PRE: regular menstruation during last 12 mths. PERI: menstrual cycle  length more irregular in preceding 12 mths, or stopped menstruating for between 3-12  mths. POST: 12 consecutive mths of amenorrhoea | Abdominal pain  Bloating | Menopause-specific questionnaire, designed by Kaczmarek | Not stated | Menopausal stage (PRE vs PERI + POST)  Genetic markers (CYP1B1 Leu432Val polymorphism) |
| Luque (2012)^77^ | Cross-sectional | USA  (North America) | PERI | Experiencing irregular or infrequent menstrual cycles in past 12 mths | Bloating | MENQOL | Not stated | Physical activity |
| Lyndaker (2004)^147^ | Cross-sectional | USA  (North America) | PERI | Recruited by age (30-50yrs) | Constipation | Menopause Symptom List (Perz, 1997) | Not stated | - |
| Ma (2016)^149^ | Longitudinal | USA  (North America) | PRE; POST (never used hormone); POST (current hormone user); POST (past hormone user) | Not stated | Constipation | Not stated | Not stated | Menopausal stage (PRE vs POST-never used vs POST-current user vs POST past hormone user) |
| Ma (2017)^148^ | Cross-sectional | China  (Asia) | PERI | Recruited by age 40-60yrs; amenorrhoea >1yr excluded | Constipation | Study-specific tool | Not stated | Perimenopausal syndrome (as assessed by Kupperman Index) |
| Mahajan (2012)^150^ | Cross-sectional | India  (Asia) | POST | More than 1 yr of amenorrhoea | Constipation  Diarrhoea | General Health Questionnaire | Not stated | Age |
| Maiello (2022)^151^ | Cross-sectional | Italy  (Europe) | PRE; POST | Not stated | Heartburn | Diagnostic criteria from American College of Gastroenterology | Not stated | Menopausal stage (PRE vs POST) |
| Manonai (2010)^152^ | Cross-sectional | Thailand  (Asia) | PRE; POST | Not stated | Constipation  Faecal incontinence | Urogenital Distress Inventory Short Form (UDI-6) plus 6-item anorectal symptom questionnaire | Previous 3-mths | Menopausal stage (PRE vs POST) |
| Markland (2017)^153^ | Longitudinal | USA  (North America) | PRE; POST | Not stated | Constipation  Diarrhoea  Faecal incontinence | Fecal Incontinence Severity Index (FISI); Colorectal Anal Distress Inventory (CRADI) | Not stated | Menopausal stage (PRE vs POST) |
| Martinez-Vazquez (2023)^154^ | Cross-sectional | Spain  (Europe) | POST | Not menstruated for >12 mths who self-reported being postmenopausal | Faecal incontinence  Gastrointestinal problems (not specified) | Study-specific tool. 12-item short-form survey (SF-12) questionnaire | Not stated | Body Mass Index (normal weight; overweight; obese) |
| Meriggiola (2012)^155^ | Cross-sectional | Italy  (Europe) | POST | Based on follicle-stimulating hormone and estradiol values and spontaneously occurring amenorrhea for at least 1 yr | Abdominal pain | Pain intensity: Visual Analogue Scale (0-10); Patient Global Impression of Change (PGIC) to assess change in pain that started PRE and persisted into POST | Not stated | Menopausal stage (PRE vs POST) |
| Methot (2004)^156^ | Cross-sectional | Canada  (North America) | PRE; POST: POST-HRT | Not stated | Heartburn  Nausea | Study-specific tool | Not stated | Menopausal stage (PRE vs POST vs POST-HRT) |
| Mikhail (1996)^157^ | Cross-sectional | Egypt  (Africa) | PERI | Recruited based on age (40-60yrs) | Heartburn | Study-specific tool | Previous 6-mths | - |
| Mishra (2002)^158^ | Longitudinal | Australia  (Oceania) | PRE; PERI; POST; POST-HRT | Self-report. PRE: menstrual bleeding in past 3 mths, and in past 12 mths with the same frequency as in yr prior;  PERI: menstrual bleeding in past 12 mths, but not in past 3 mths or with different menstrual frequency  compared with previous yr; POST: no menstrual bleeding in past 12 mths. | Constipation | Study-specific tool | Previous 12-mths | Menopausal stage (PRE vs PERI vs POST vs POST-HRT) |
| Mishra (2012)^159^ | Longitudinal | Australia (Oceania) | PRE; PERI; POST | PRE: menstruated last 3 mths/no change in menstrual frequency in last 12 mths; PERI: changes in menstrual frequency OR 3-11 mths of amenorrhoea. POST: amenorrhoea for ≥12 mths | Heartburn | Study-specific tool | Previous 12-months | Associated clusters of menopausal symptoms |
| Morse (1998)^160^ | Longitudinal | Australia  (Oceania) | Early PERI; late PERI; POST | PERI: change to flow/ frequency or reported amenorrhea occurring between 3-12 mths over last 12mths; POST: absence of periods for at least past 12 consecutive mths. No definition provided for late vs early PERI | Digestive symptoms (not specified) | 22-item symptom questionnaire from McKinley | Previous 2-wks | Premenstrual complaints |
| Mous (2008)^161^ | Case-control | Neth-erlands  (Europe) | PRE; POST | Not stated | Faecal incontinence | Park’s Classification | Not stated | Menopausal stage (PRE vs POST)  Comorbidities (obstetric anal sphincter rupture) |
| Oğurlu (2011)^162^ | Cross-sectional | Turkey  (Asia) | POST | No menstruation for at least 1 yr. | Bloating  Constipation | Climacteric Complaint Tool (CCT) | Not stated | Employment status (working vs non-working) |
| Ojha (2022)^163^ | Cross-sectional | India  (Asia) | POST | Not stated | GI problems (not specified) | Questionnaire – details not provided | Not stated | - |
| Olafsdottir (2012)^164^ | Longitudinal | Iceland  (Europe) | PRE; POST | Menopause was identified in women who were menstruating in 1996 but reported that they did not  menstruate in 2006. | Abdominal pain | Bowel Disease Questionnaire (47 GI symptoms) - Icelandic version; Manning criteria to identify IBS | Previous 12 mths | Menopausal stage (PRE vs POST)  Dysmenorrhoea during menstruation |
| Özcan (2019)^165^ | Cross-sectional | Turkey  (Asia) | POST | Not stated | Constipation | Not stated | Not stated | Comorbidities (lower urinary tract symptoms) |
| Parandavar (2014)^166^ | Mixed methods | Iran  (Asia) | POST | At least 6 mths from last menstrual cycle | Constipation  Diarrhoea | Not stated | Not stated | - |
| Parsons (2023)^167^ | Qualitative; semi-structured interviews | UK  (Europe) | PRE; POST | Not stated | Faecal incontinence | Semi-structured interviews | Not stated | Menopausal stage (PRE vs POST); Obstetric history (experiences of FI following vaginal birth) |
| Resmi (2020)^168^ | Cross-sectional | India  (Asia) | PRE; PERI; POST | STRAW. PRE: minor changes in cycle length particularly, decreasing  length of cycle; early PERI: increasing irregularity of menses without skipping periods; Late PERI: menstruation in last 2–12 mths but  not last 2 mths. POST: no menstrual bleeding in last 12 mths. | Abdominal pain | Study-specific tool | Not stated | - |
| Rizk (2001)^169^ | Cross-sectional | UAE  (Asia) | PRE; POST | Not stated | Faecal incontinence | Study-specific tool | Previous 12 mths | Menopausal stage (PRE vs POST) |
| Safaee (2011)^170^ | Cross-sectional | Iran  (Asia) | PRE; PERI | Not stated | Bloating  (IBS symptoms) | Self-report of bloating | Not stated | Menopausal stage (PRE vs PERI)  Comorbidities (menstrual symptoms) |
| *Salmoirago-Blotcher (2011) | Longitudinal | USA  (North America) | POST | Recruited by age (50-79 yrs) | Constipation | Study-specific tool | Previous 4 wks | Comorbidities (death from coronary heart disease, non-fatal myocardial infarction, angina, coronary revascularization, stroke and transient ischemic  attack) |
| Samtani (2020)^172^ | Cross-sectional | India  (Asia) | POST | Not stated | Abdominal pain  Heartburn | Study-specific tool | Not stated | Age |
| Selcuk (2012)^28^ | Cross-sectional | Turkey  (Asia) | PRE; POST | Not stated | Faecal incontinence | Wexner Incontinence Scale | Not stated | Menopausal stage (PRE vs POST)  Comorbidities (urinary incontinence) |
| Senthilvel (2018)^173^ | Cross-sectional | India  (Asia) | POST | >1 yr of amenorrhoea | Bloating | MENQOL | Not stated | - |
| Sharma (1981)^176^ | Cross-sectional | India  (Asia) | PRE; PERI: POST | PRE: normal menses during yr preceding survey; PERI: menstrual pattern different from the former pattern; POST: did not menstruate in yr preceding survey | Constipation  Diarrhoea | Checklist based on Neugarten (4-pt scale) | Previous 12 mths | Menopausal stage (PRE vs PERI vs POST) |
| Sharma (2007)^175^ | Cross-sectional | India  (Asia) | POST | Not stated | Constipation  Diarrhoea | 30-item checklist derived from Greene and Neugarten/Kraines | Not stated | Age |
| Sheereen (2022)^177^ | Cross-sectional | India  (Asia) | POST | >1 yr of amenorrhoea | Bloating | MENQOL | Not stated | - |
| Slopien (2018)^178^ | Cross-sectional | Poland  (Europe) | Late MT; early POST | Late MT: still menstruating with period of amenorrhoea of ≥60 days; early POST: at least 1 yr since last menses. | Digestive tract symptoms (not specified) | Hamilton Depression Scale | Not stated | Menopausal stage (late MT vs early POST)  Other assays (serum allopregnalone) |
| Sood (2016)^179^ | Cross-sectional | USA  (North America) | PERI; POST | PERI: changes in periods but not yet experienced 12 consecutive mths without a period; POST: after 12 consecutive mths without a period | Bloating | Menopause Health Questionnaire (MHQ) | Not stated | Association between symptoms and negative view of menopause |
| Stewart (2003)^180^ | Qualitative | Guatemala  (North America) | PERI; POST | Recruited by age (38-55yrs) who had noticed periods changing or whose periods stopped during the last 3 yrs | Abdominal pain | Interviews | Not stated | - |
| Terauchi (2013)^181^ | Cross-sectional | Japan  (Asia) | MT; POST | MT: a menstrual period within past 12 mths but missed a period/irregular cycles in past 3 mths; POST: not had period in past 12 mths. | Nausea | Menopausal Health-Related Quality of Life (MHR-QOL) | Previous 1 mth | Comorbidities (anxiety/depression as assessed by the Hospital Anxiety and Depression Scale (HADS)) |
| Terzic (2024)^182^ | Cross-sectional | Kazakhstan  (Asia) | PRE; PERI; POST | Self-reported; PRE: regular periods; PERI; changes in periods for women >45 yrs but not having complete  12 mths without a period; POST: after 12 mths of complete amenorrhoea. | Bloating | MENQOL | Not stated | Menopausal stage (PRE vs PERI vs POST) |
| Tokunaga (2014)^183^ | Cross-sectional | Japan  (Asia) | PRE; POST | PRE: ‘Menstruating’; POST: not stated | Abdominal pain  Constipation | Not stated | Not stated | Menopausal stage (PRE vs POST)  Comorbidities (hie – sensitivity to coldness) |
| Wang (2024)^78^ | Cross-sectional | China  (Asia) | POST | >1 yr of menopause | Abdominal pain  Constipation  Diarrhoea  Heartburn | Gastrointestinal Symptom Rating Scale (GSRS): 15 items | Not stated | Comorbidities (osteoporosis) |
| *Wisniewska-Jarosinska (2010)^184^ | Cross-sectional | Poland  (Europe) | PRE; POST | Recruited women 2-18 yrs since menstruation with mean FSH concentration 93.4 IU/l. | Constipation  Diarrhoea  (IBS symptoms) | Study-specific tool (10-pt scale) | Not stated | Other assays (Urinary 6-hydroxymelatonin sulphate (6-HMS) |
| Yerra (2021)^185^ | Cross-sectional | India  (Asia) | POST | Natural cessation of menstrual period ≥12 mths | Bloating | MENQOL | Previous mth | - |
| Yim (2015)^186^ | Cross-sectional | South Korea  (Asia) | PRE; early MT; late MT; POST | STRAW. PRE: regular menstrual periods; early PERI transition: ≥2 cycles with a difference in cycle length of ≥7 days; late PERI transition: 2 skipped cycles and amenorrhea  interval of ≥60 days; POST: period after 12 consecutive mths of amenorrhoea | Bloating | MENQOL | Not stated | Menopausal stage (early MT vs late MT vs POST) |
| Zhu (2020)^187^ | Case-control | China  (Asia) | POST | Without menstruation for at least 12 mths | Constipation | Study-specific tool | Previous 12 mths | Comorbidities (recurrent urinary tract infections) |
| LRS: late reproductive stage; PRE: premenopausal; PERI: perimenopausal; MT: menopausal transition; POST: postmenopausal; IBS: irritable bowel syndrome; *denotes study with primary objective relating to GI symptoms and peri- or postmenopause; hr(s): hours; mth(s): month(s); wk(s): week(s); yr(s): year(s)  Countries classified into regions using OurWorldInData categories^76^ | | | | | | | | |
